# Chemoselectively Functionalized Ketoesters by Halogenative C–C Bond Cleavage of Cyclic Diketones

**DOI:** 10.3390/molecules31010199

**Published:** 2026-01-05

**Authors:** Hideyasu China, Nami Kageyama, Hodaka Yatabe, Mihoyo Fujitake, Yusei Matsumoto, Zhihan Jing, Toshifumi Dohi

**Affiliations:** 1 Faculty of Pharmaceutical Sciences, Doshisha Women’s College of Liberal Arts, 97-1, Minamihokotate, Kodo, Kyotanabe 610-0395, Kyoto, Japan; 2 College of Pharmaceutical Sciences, Ritsumeikan University, 1-1-1 Nojihigashi, Kusatsu 525-8577, Shiga, Japan; ph0093ei@ed.ritsumei.ac.jp (N.K.); ph0090ir@ed.ritsumei.ac.jp (H.Y.); ph0196hh@ed.ritsumei.ac.jp (Y.M.); gr0761hr@ed.ritsumei.ac.jp (Z.J.); 3 Center for Pharmaceutical Research and Development, Osaka Medical and Pharmaceutical University, 4-20-1 Nasahara, Takatsuki 569-1094, Osaka, Japan; fujitake@gly.oups.ac.jp

**Keywords:** ketoesters, functionalization, halogenation, C–C bond cleavage, cyclic diketone, ring-opening reaction

## Abstract

Haloketoesters are synthetic intermediates in various cyclization reactions that facilitate the production of biologically active compounds. Nonetheless, the selective synthesis of dihaloketoesters and trihaloketoesters, which are expected to be highly versatile, presents significant challenges. In this study, we designed a new synthetic approach that selectively and efficiently produces haloketoesters through the halogenative C–C bond cleavage and ring-opening reactions of cyclic 1,3-diketones. This convenient method enables the direct synthesis of di- and trichloro-functionalized ketoesters from 1,3-cyclohexadiones under mild conditions. Na_2_HPO_4_, employed as a buffer salt, proved to be effective in facilitating the alcoholytic ring-opening reaction of 2,2-dichloro-1,3-cyclohexadiones, which were generated as synthetic intermediates.

## 1. Introduction

Acyclic keto acids and ketoesters are amenable to a range of cyclization reactions, resulting in diverse ring structures including disubstituted lactams [1,2,3,4], acyllactams [5], disubstituted cyclic amines [6,7], monosubstituted lactones [8,9,10,11], *gem*-disubstituted lactones [12,13], monosubstituted enol lactones [14], acyllactones [15,16], other heterocycles [17,18], and carbocyclic compounds [19]. These acyclic molecules can be synthesized through various strategies such as the retro-Claisen reaction [20,21,22,23], Michael reaction [24,25,26], directed oxidation reaction [27], oxidative ring-opening reaction [28,29,30,31], cross-coupling reaction [32,33], and insertion reaction [34]. These methodologies facilitate the introduction of diverse substituents and enable a variety of synthetic approaches from the appropriate starting materials.

**Figure 1:**
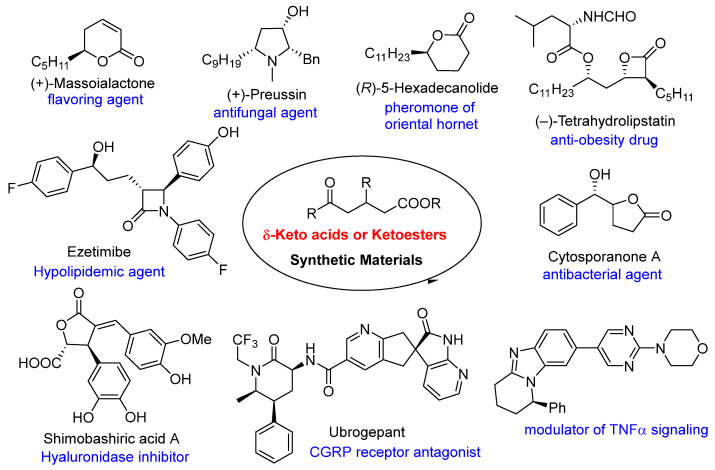
Bioactive compounds synthesized using δ-keto acids and ketoesters as intermediates.

Keto acids and ketoesters are recognized for their inclusion in various bioactive compounds, such as duazomycin (*N*-acetyl DON) [35], esonarimod (KE-298) [36], (*R*)-flobufen [37], kynurenine [38], tanomastat (BAY 12-9566) [39], and 3F-5-AL [40], rendering them significant synthetic targets. Furthermore, keto acids and ketoesters serve as excellent synthetic building blocks, possessing at least two reactive sites with distinct chemical properties. Indeed, numerous synthetic methodologies have been developed utilizing these compounds as key molecules for the synthesis of bioactive compounds, including cytosporanone A, massoialactone, preussin, hexadecanolide, tetrahydrolipstatin, ezetimibe, shimobashiric acid A, ubrogepant, and several unnamed substances (Figure 1) [1,15,41,42,43,44,45].

In particular, functionalized haloketoesters are highly versatile in their derivatization. Dihaloacyl groups are applicable to asymmetric reduction [46,47,48], aldol reactions [49,50,51,52], *Z*-alkenoate formation [53,54,55], [4+3] cycloaddition [53,55], and various heterocycle formations [53,56,57,58,59]. Furthermore, trihaloacyl groups have been employed in asymmetric Mannich-type reactions [60,61,62], asymmetric reduction followed by Jocic-type reactions [63], radical addition reactions [64], and Grignard reactions [65]. Trihaloacyl groups also serve as leaving groups during alcoholysis [66] and aminolysis [67]. Our previous research demonstrated that haloketoesters, which possess both a haloacyl moiety and an ester moiety, provide additional versatility in derivatizations [68,69,70].

However, the selective synthesis of di- and trihaloketoesters via ketoester halogenation presents significant challenges. This difficulty arises because the introduction of halogen atoms at the ketone α-position competes with halogenation at the alternative α’-position. Additionally, overreactions such as trihalogenation (e.g., haloform reactions) complicate the synthesis of dihalogenated compounds, rendering it exceedingly difficult to control both the regioselectivity and the number of halogen atoms introduced.

**Scheme 1:**
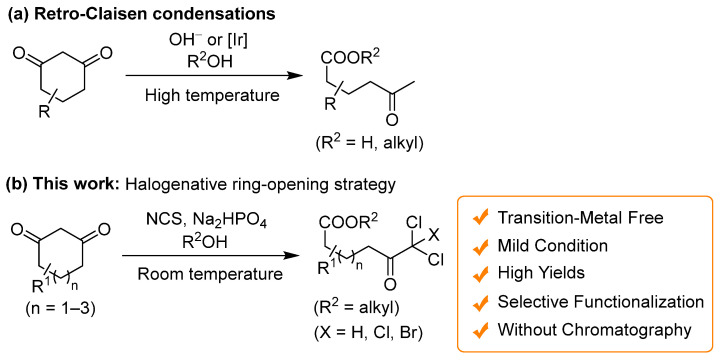
Ring-cleavage reactions of cyclic 1,3-diketones: (**a**) Retro-Claisen condensations; (**b**) This work: Halogenative ring-opening strategy.

This study focuses on selective halogenation via ring-opening reactions of cyclic 1,3-diketones, which are readily synthesized using various methodologies [71,72,73,74,75,76,77]. Traditional approaches to reagent-controlled ring-opening reactions employ strong bases, as well as metal-catalyst-controlled retro-Claisen condensations [20], albeit requiring elevated temperatures. These methods facilitate the formation of non-functionalized ring-opening products from cyclic 1,3-diketones (Scheme 1a). Integrating halogenation under harsh conditions is not conducive to the selective synthesis of haloketoesters. In this study, we successfully developed a direct and selective synthesis method for di- and trihaloketoesters from cyclic 1,3-diketones, eliminating the need for transition metals, through convenient halogenative ring-opening esterification under mild conditions (Scheme 1b). Notably, the synthesis of dichloroketo acids was accomplished without using chromatography. Our approach for synthesizing functionalized ketoesters has the potential to advance the molecular transformation of 1,3-cyclohexadiones into diverse heterocyclic compounds.

## 2. Results and Discussion

### 2.1. Optimization of Buffer Salt for Halogenative Ring-Opening Reaction

The *gem*-dihalomethylketone moiety is a potent site for C–C bond cleavage. Initial halogenative C–C bond cleavage reactions of acyclic molecules are primarily of interest for their degradative fragmentation properties [78]. In recent years, however, synthetically valuable C–C bond cleavage reactions have been developed, including *gem*-difluorination [79,80], dichlorination [81,82,83,84,85], and dibromination [81,83], through the use of halogenating agents and nucleophiles. Analogous reactions have been implicated in the biosynthesis of biologically active compounds [86,87]. However, halogenative C–C bond cleavage reactions involving the ring opening of cyclic molecules have not been extensively explored for synthetic applications. Although such reactions have been conducted using alcohols [88,89,90], water [91], and amines [92,93,94] as nucleophiles, they are generally restricted to ring-strained substrates and often require harsh conditions such as high temperatures and strong bases. The dichloroacetylation of amines, employing cyclic *gem*-dichlorodiester as the sacrificial agent, utilizes a comparable C–C bond cleavage process [95].

Building upon the product analysis of multiple reactions observed during the monochlorodimedone assay [69], we previously demonstrated the stepwise cleavage of the C–C bond in a cyclic substrate devoid of ring strain [70]. Nevertheless, C–C bond cleavage accompanied by the dichlorination of cyclic diketones under one-pot conditions has not been demonstrated.

This study focuses on the direct synthesis of di- or trichloroketo acid methyl esters from cyclic 1,3-diketones under considerably mild conditions in an alcoholic medium containing *N*-chlorosuccinimide (NCS) and buffer salts at ambient temperature. Initially, the influence of buffer salts on the conversion of cyclohexadione **1a** to dichloroketoester **2a** in the presence of MeOH was assessed, and the results are presented in Table 1. The sodium salt exhibited a higher product yield than the potassium salt. Moreover, weakly basic salts such as Na_2_HPO_4_, NaHCO_3_, and AcONa (entries 1, 5, and 9, respectively) were determined to be more effective than NaH_2_PO_4_, a weakly acidic salt (entry 3), and Na_2_CO_3_, a relatively strong base (entry 7). Consequently, Na_2_HPO_4_ was identified as the optimal salt for the halogenative ring cleavage. When a catalytic amount of the base was used, the reaction was stopped after 30 min. Therefore, a minimum of 0.5 equivalents of Na_2_HPO_4_ was required for the reaction to proceed completely.

### 2.2. Substrate Scope

The substrate scope was evaluated under the optimized conditions using Na_2_HPO_4_ (Table 2). For the synthesis of 5-substituted dichloroketo acid methyl esters **2b**–**g** from the corresponding 1,3-cyclohexadiones **1b**–**g**, 2.0 equivalents of NCS were used in the presence of 0.5 equivalents of Na_2_HPO_4_. This direct synthesis yielded monosubstituted dichloroketoesters **2b**–**d** and *gem*-disubstituted dichloroketoesters **2e**–**g** with high efficiency (Table 2). The comparable yields of methyl-substituted products **2b**,**e** and phenyl-substituted products **2c**,**f** indicate that the 5-position of the corresponding substrates **1b**,**c**,**e**,**f** was not significantly influenced by steric hindrance in this reaction. Furthermore, *p*-anisyl-substituted product **2d** and *p*-tolyl-substituted product **2g** demonstrated the potential to introduce additional substituents at the aryl moiety. However, this method is unsuitable for the synthesis of dibromoketoesters because the buffer salt induces debromination of the synthetic intermediate 2,2-dibromo-1,3-cyclohexadiones [69,96].

By contrast, the utilization of 3.6 equivalents of NCS facilitated the synthesis of trichloroketo acid methyl esters **3a**–**g**. This reaction requires excess NCS beyond the stoichiometric quantities. When only 3.0 equivalents of NCS were employed, small amounts of the corresponding dichloroketoesters **2a**–**g** persisted within the reaction system as synthetic intermediates, complicating the purification of the desired corresponding trichloroketoesters **3a**–**g**. This approach successfully yielded trichloroketoesters without substituent **3**, with monosubstituents **3b**–**d** and *gem*-disubstituents **3e**–**g** in high yields (Table 3). The yields of trichloroketoesters **3a**–**g** were comparable to those of the corresponding dichloroketoesters **2a**–**g** (see Table 2).

Additionally, alcohols that functioned as co-solvents were screened. The ester moiety of the product in this reaction depends on the alcohol used. For the synthesis of dichloroketo acid alkyl ester, 2.0 equivalents of NCS and Na_2_HPO_4_ were used in a dehydrated alcohol with 3 Å molecular sieves, as detailed in Table 4. Ring-cleavage reactions with EtOH, *^n^*PrOH, and 2-methoxyethanol yielded the corresponding ethyl ester **4**, *n*-propyl ester **5**, and 2-methoxyethyl ester **6**, respectively, with high efficiency (entries 1–3). Conversely, ring cleavage using *^n^*BuOH and *^i^*BuOH afforded the corresponding *n*-butyl ester **7** and *i*-butyl ester **8**, respectively, in low yields (entries 4 and 5). As mentioned above, these esters are produced in the presence of primary alcohols. However, when the secondary alcohol *^i^*PrOH or the tertiary alcohol *^t^*BuOH was employed, the corresponding esters were not formed; instead, the reactions resulted in the formation of dichloroketo acids. This indicates that the steric hindrance of the alcohol side chain significantly influences the nucleophilic reaction of alcohols with *gem*-dichlorinated synthetic intermediates.

**Scheme 2:**
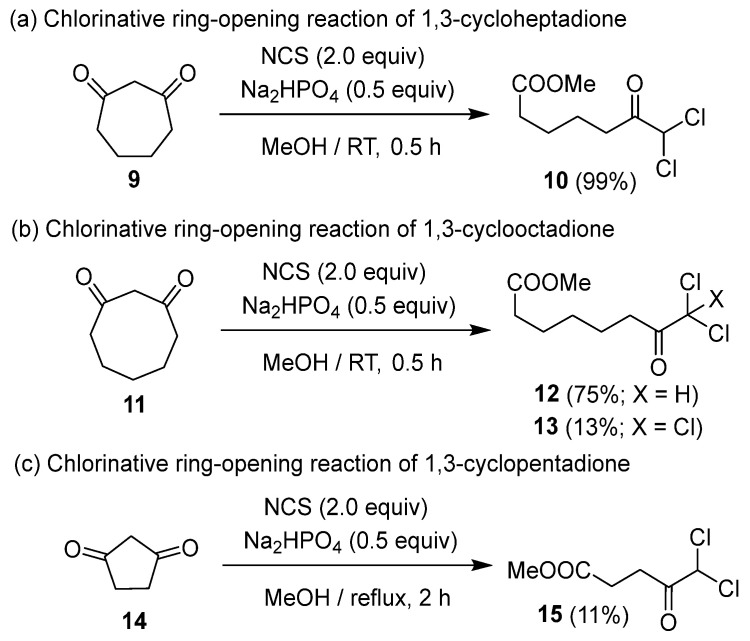
Ring-size scope for halogenative ring cleavage, of (**a**) 1,3-cycloheptadione, (**b**) 1,3-cyclooctadione, and (**c**) 1,3-cyclopentadione.

The dichlorinative ring cleavage of 1,3-cycloheptadione **9** afforded the corresponding product **10** in good yield, whereas 1,3-cyclooctadione **11** produced the corresponding dichloroketoester **12** along with trichloroketoester **13** as a byproduct (Scheme 2a,b). This implies that the chlorination of 1,3-cyclooctadione **11** is the rate-determining step of the dichlorinative ring-cleavage reaction. On the other hand, 1,3-cyclopentanone **14** did not undergo chlorination at ambient temperature; however, the corresponding dichloroketoester **15** was obtained in low yield under reflux conditions (Scheme 2c). The inability to synthesize **15** using Cs_2_CO_3_, a stronger base, indicates that Na_2_HPO_4_, a weaker base, is more suitable for this reaction.

### 2.3. Mechanism

**Scheme 3:**
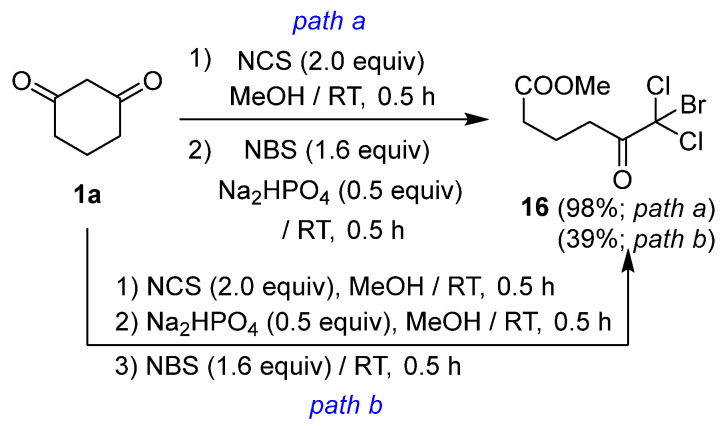
Control experiment for trihalogenative ring-cleavage reactions.

Mixed trihaloketoesters can be synthesized through a stepwise approach involving *gem*-chlorination of cyclic 1,3-diketones, followed by alcoholic ring cleavage of the resulting cyclic dichloro-1,3-diketones. For instance, dichloromonobromoketoester **16** was obtained in 98% yield via *gem*-chlorination of cyclic diketone **1a**, followed by the addition of Na_2_HPO_4_ in the presence of NBS (Scheme 3, path a). However, the addition of NBS after the ring-cleavage reaction with Na_2_HPO_4_ resulted in a reduced yield of 39% (Scheme 3, path b). This observation indicates that the enolate generated immediately prior to the ring-cleavage reaction served as a highly reactive intermediate for the third subsequent halogenation.

**Figure 2:**
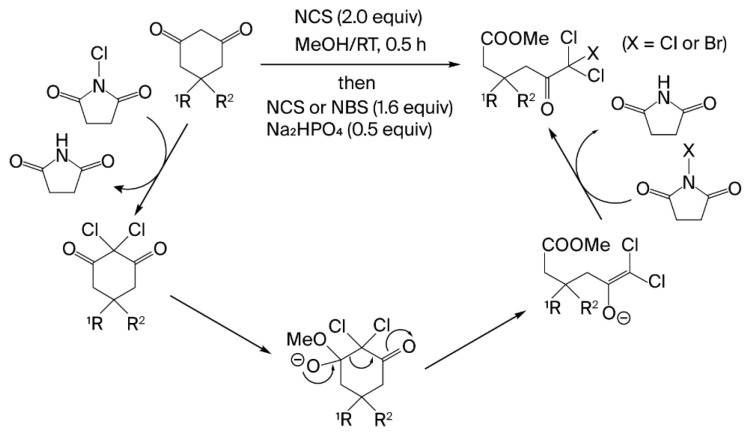
Mechanism of halogenative ring-cleavage reaction producing trihaloketoester.

Halogenative ring cleavage of cyclic 1,3-diketones involves two independent processes: *gem*-dichlorination and alcoholic ring opening (Figure 2). In the *gem*-dichlorination process, the reaction proceeds quantitatively—even in the absence of Na_2_HPO_4_—because the succinimide anion generated from NCS during chlorination acts as a base. The carbonyl group of the cyclic 1,3-diketone, which completely suppresses enol–enol isomerization via *gem*-dichlorination, regains its electrophilicity, thereby facilitating nucleophilic reactions with alcohols. However, the alcohol ring-opening process requires Na_2_HPO_4_. The recarbonylation of the alkoxy anion generated by the nucleophilic reaction of alcohols drives the irreversible cleavage of the C–C bond, while the intramolecular *gem*-chloroacetyl moiety aids in charge stabilization. Consequently, an environment with excess halogenating agent for *gem*-dichlorination resulted in the formation of a trihaloketoester from the enol anion, whereas an environment lacking excess halogenating agent yielded a dihaloketoester, as the enol anion was readily protonated by the surrounding protic solvent. Although the reaction mechanism is similar to the classical Lieben haloform reaction, it differs in the number of required halogen atoms and the reaction selectivity for C–C bond cleavage. Our method requires dihalogenation for the cleavage of cyclic 1,3-diketones, whereas the haloform reaction typically requires trihalogenation for substrates containing an acetyl group. Another distinction is that the trihaloacetyl moiety in the trihaloketoesters is retained without further fragmentation in our method. This unique chemoselectivity is synthetically advantageous.

## 3. Materials and Methods

### 3.1. General Information

5-(*p*-Methoxyphenyl)-1,3-cyclohexanedione **1d [97]** was prepared from 4-(*p*-methoxyphenyl)-3-butene-2-one and diethyl malonate according to the literature. 5-Methyl-5-phenyl-1,3-cyclohexanedione **1f [98]** and 5-methyl-5-*p*-tolyl-1,3-cyclohexanedione **1g** were prepared from 5-methylresorcinol according to the literature. ^1^H and ^13^C nuclear magnetic resonance (NMR) spectra were recorded on an ECS 400 and ECX 500 NMR spectrometer (JEOL Ltd., Tokyo, Japan) using deuterated chloroform (CDCl_3_) as the solvent. Chemical shifts (*δ*) are reported in ppm relative to tetramethylsilane (*δ* = 0) as an internal standard. The coupling constants (*J*) are reported in Hertz, and the multiplicity is reported according to the following convention: singlet (s), doublet (d), double doublet (dd), triplet (t), quintet (quin), sextet (sext), octet (o), broad singlet (br), and multiplet (m). The chemical shifts (number of protons, multiplicity, and coupling constants) are reported. Infrared (IR) spectra were recorded using a JASCO FT/IR-4200 spectrometer (JASCO, Tokyo, Japan) using the diffuse reflectance method with KBr powder. Absorption is expressed in units of reciprocal centimeters (cm^−1^). High-resolution mass spectra (HRMS) were obtained using the electron ionization (EI) method and recorded using a JMS-700 spectrometer (JEOL Ltd., Tokyo, Japan).

### 3.2. Synthesis of Haloketo Acid Alkyl Esters

#### 3.2.1. General Procedure for Synthesizing Dichloroketo Acid Methyl Esters

To a solution of a 1,3-cyclohexadione (1.0 mmol) and NCS (267.1 mg, 2.0 mmol) in MeOH (10 mL), Na_2_HPO_4_ (71 mg, 0.5 mmol) was added. After stirring the mixture at room temperature for 0.5 h, the solvent was evaporated. The product was dissolved in CH_2_Cl_2_, and the extract was filtered through filter paper for dehydration. After evaporation to remove the solvent, the product was dissolved in hexane by sonication, and the extract was filtered through filter paper. The removal of the solvent by evaporation afforded the purified product.

#### 3.2.2. *6,6-Dichloro-5-oxo-hexanoic Acid Methyl Ester* (**2a**)

Colorless oil. ^1^H NMR (400 MHz, CDCl_3_): *δ* 2.00 (2H, quin, *J* = 7.2 Hz), 2.40 (2H, t, *J* = 7.3 Hz), 2.92 (2H, t, *J* = 7.1 Hz), 3.69 (3H, s), 5.84 (1H, s) ppm. ^13^C NMR (100 MHz, CDCl_3_): *δ* 18.9, 32.6, 33.9, 51.7, 69.7, 173.3, 196.7 ppm. IR (ATR, KBr): *ν* 3649, 3545, 3456, 2953, 2848, 2596, 2411, 2068, 1733, 1606, 1438, 1372, 1210, 1088, 1053, 892, 867, 782, 727 cm^−1^. HRMS (EI, *m*/*z*) calcd for C_7_H_11_Cl_2_O_3_ [M + 1]^+^: 213.0085; found: 213.0079.

#### 3.2.3. *6,6-Dichloro-3-methyl-5-oxo-hexanoic Acid Methyl Ester* (**2b**)

Colorless oil. ^1^H NMR (400 MHz, CDCl_3_): *δ* 1.04 (3H, d, *J* = 6.9 Hz), 2.30 (1H, dd, *J* = 15.6, 7.3 Hz), 2.38 (1H, dd, *J* = 15.6, 6.4 Hz), 2.57 (1H, o, *J* = 6.9 Hz), 2.79 (1H, dd, *J* = 17.8, 7.3 Hz), 2.91 (1H, dd, *J* = 17.9, 6.2 Hz), 3.68 (3H, s), 5.82 (1H, s) ppm. ^13^C NMR (100 MHz, CDCl_3_): *δ* 19.8, 26.2, 40.2, 41.1, 51.6, 70.0, 172.6, 195.9 ppm. IR (ATR, KBr): *ν* 3656, 3620, 3539, 3456, 2968, 2845, 2741, 2610, 2019, 1736, 1437, 1370, 1171, 1078, 1039, 1009, 957, 858, 784, 733, 663, 551 cm^−1^. HRMS (EI, *m*/*z*) calcd for C_8_H_13_Cl_2_O_3_ [M + 1]^+^: 227.0242; found: 227.0240.

#### 3.2.4. *6,6-Dichloro-5-oxo-3-phenyl-hexanoic Acid Methyl Ester* (**2c**)

Colorless oil. ^1^H NMR (400 MHz, CDCl_3_): *δ* 2.68 (1H, dd, *J* = 15.6, 7.4 Hz), 2.74 (1H, dd, *J* = 16.0, 7.3 Hz), 3.23 (2H, d, *J* = 7.3 Hz), 3.61 (3H, s), 3.74 (1H, quin, *J* = 7.3 Hz), 5.71 (1H, s), 7.20–7.26 (3H, m), 7.28–7.34 (2H, m) ppm. ^13^C NMR (100 MHz, CDCl_3_): *δ* 37.0, 40.1, 41.0, 51.7, 69.9, 127.2, 127.2, 128.7, 142.1, 172.0, 195.0 ppm. IR (ATR, KBr): *ν* 3063, 3030, 3005, 2952, 2845, 1954, 1878, 1735, 1604, 1496, 1455, 1438, 1368, 1336, 1265, 1221, 1161, 1127, 1081, 1028, 993, 889, 845, 793, 764, 733, 700, 562, 522 cm^−1^. HRMS (EI, *m*/*z*) calcd for C_13_H_14_Cl_2_O_3_ [M]^+^: 288.0320; found: 288.0323.

#### 3.2.5. *6,6-Dichloro-3-(4-methoxy-phenyl)-5-oxo-hexanoic Acid Methyl Ester* (**2d**)

Colorless oil. ^1^H NMR (400 MHz, CDCl_3_): *δ* 2.64 (1H, dd, *J* = 15.1, 7.8 Hz), 2.70 (1H, dd, *J* = 16.0, 7.3 Hz), 3.18 (2H, d, *J* = 7.3 Hz), 3.60 (3H, s), 3.78 (3H, s), 5.70 (1H, s), 6.83 (2H, d, *J* = 8.7 Hz), 7.15 (2H, d, *J* = 8.7 Hz) ppm. ^13^C NMR (100 MHz, CDCl_3_): *δ* 36.3, 40.3, 41.2, 51.7, 55.2, 69.9, 114.0, 128.3, 134.0, 158.5, 172.0, 195.0 ppm. IR (ATR, KBr): *ν* 3659, 3562, 3459, 3002, 2954, 2838, 2602, 2549, 2493, 2426, 2059, 1883, 1736, 1612, 1583, 1514, 1439, 1366, 1250, 1084, 1031, 830, 794, 743, 556, 539 cm^−1^. HRMS (EI, *m*/*z*) calcd for C_14_H_16_Cl_2_O_4_ [M]^+^: 318.0426; found: 318.0426.

#### 3.2.6. *6,6-Dichloro-3,3-dimethyl-5-oxo-hexanoic Acid Methyl Ester* (**2e**)

Colorless oil. ^1^H NMR (400 MHz, CDCl_3_): *δ* 1.14 (6H, s), 2.50 (2H, s), 2.97 (2H, s), 3.65 (3H, s), 5.83 (1H, s) ppm. ^13^C NMR (100 MHz, CDCl_3_): *δ* 28.1, 32.6, 44.0, 44.3, 51.3, 70.5, 172.4, 195.7 ppm. ^1^H and ^13^C NMR data are consistent with those reported in the literature [67].

#### 3.2.7. *6,6-Dichloro-3-methyl-5-oxo-3-phenyl-hexanoic Acid Methyl Ester* (**2f**)

Colorless oil. ^1^H NMR (400 MHz, CDCl_3_): *δ* 2.88 (1H, d, *J* = 15.1 Hz), 2.98 (1H, d, *J* = 15.1 Hz), 3.43 (1H, d, *J* = 17.8 Hz), 3.50 (1H, d, *J* = 17.8 Hz), 3.58 (3H, s), 5.61 (1H, s), 7.20–7.25 (1H, m), 7.30–7.35 (4H, m) ppm. ^13^C NMR (100 MHz, CDCl_3_): *δ* 26.1, 39.0, 44.5, 44.9, 51.5, 70.4, 125.2, 126.7, 128.5, 145.4, 171.9, 194.7 ppm. IR (ATR, KBr): *ν* 3673, 3561, 3455, 3091, 3060, 2952, 2844, 2602, 1952, 1874, 1730, 1602, 1582, 1497, 1444, 1382, 1351, 1209, 1078, 1011, 956, 850, 766, 738, 697, 633, 560, 540, 525 cm^−1^. HRMS (EI, *m*/*z*) calcd for C_14_H_16_Cl_2_O_3_ [M]^+^: 302.0476; found: 302.0470.

#### 3.2.8. *6,6-Dichloro-3-methyl-5-oxo-3-p-tolyl-hexanoic Acid Methyl Ester* (**2g**)

Colorless oil. ^1^H NMR (400 MHz, CDCl_3_): *δ* 2.88 (3H, s), 2.31 (3H, s), 2.86 (1H, d, *J* = 15.1 Hz), 2.95 (1H, d, *J* = 15.1 Hz), 3.40 (1H, d, *J* = 17.9 Hz), 3.46 (1H, d, *J* = 17.8 Hz), 3.58 (3H, s), 5.60 (1H, s), 7.13 (2H, d, *J* = 8.3 Hz), 7.21 (2H, d, *J* = 8.7 Hz) ppm. ^13^C NMR (100 MHz, CDCl_3_): *δ* 20.9, 26.2, 38.7, 44.5, 45.1, 51.4, 70.4, 125.1, 129.2, 136.2, 142.4, 171.9, 194.8 ppm. HRMS (EI, *m*/*z*) calcd for C_15_H_18_Cl_2_O_3_ [M]^+^: 316.0633; found: 316.0628.

#### 3.2.9. General Procedure for Synthesis of Trichloroketo Acid Methyl Esters

To a solution of 1,3-cyclohexadiones (1.0 mmol) and NCS (480.7 mg, 3.6 mmol) in MeOH (10 mL), Na_2_HPO_4_ (71.0 mg, 0.5 mmol) was added. After the mixture was stirred at room temperature for 0.5 h, the solvent was removed by evaporation. The product was dissolved in CH_2_Cl_2_, and then the extract was filtered through filter paper for dehydration. After evaporation for the removal of the solvent, the product was dissolved in hexane by sonication, and then the extract was filtered through filter paper. The concentrated filtrate by evaporation was purified by silica gel chromatography with AcOEt/hexane solution.

#### 3.2.10. *6,6,6-Trichloro-5-oxo-hexanoic Acid Methyl Ester* (**3a**)

Colorless oil. ^1^H NMR (400 MHz, CDCl_3_): *δ* 2.08 (2H, quin, *J* = 7.2 Hz), 2.44 (2H, t, *J* = 7.1 Hz), 3.11 (1H, t, *J* = 7.2 Hz), 3.69 (3H, s) ppm. ^13^C NMR (100 MHz, CDCl_3_): *δ* 19.9, 32.4, 32.9, 51.7, 96.2, 173.0, 190.1 ppm. IR (ATR, KBr): *ν* 3482, 2957, 2880, 2846, 2364, 2357, 2330, 1737, 1438, 1370, 1266, 1171, 1104, 1062, 1010, 958, 877, 821, 752, 699, 650, 634, 591, 561 cm^−1^. HRMS (FAB, *m*/*z*) calcd for C_7_H_10_Cl_3_O_3_ [M + H]^+^: 246.9696; found: 246.9697.

#### 3.2.11. *6,6,6-Trichloro-3-methyl-5-oxo-hexanoic Acid Methyl Ester* (**3b**)

Colorless oil. ^1^H NMR (400 MHz, CDCl_3_): *δ* 1.07 (3H, d, *J* = 6.9 Hz), 2.34 (1H, dd, *J* = 15.5, 7.5 Hz), 2.43 (1H, dd, *J* = 15.5, 6.9 Hz), 2.64 (1H, o, *J* = 6.8 Hz), 2.98 (1H, dd, *J* = 17.8, 7.5 Hz), 3.11 (1H, dd, *J* = 17.6, 5.9 Hz), 3.69 (3H, s) ppm. ^13^C NMR (100 MHz, CDCl_3_): *δ* 19.5, 27.0, 39.9, 40.1, 51.6, 96.4, 172.4, 189.3 ppm. IR (ATR, KBr): 3820, 3661, 3481, 2953, 2880, 2845, 2741, 2616, 2017, 1738, 1437, 1370, 1267, 1173, 1102, 1063, 1009, 957, 876, 819, 754, 704, 592, 553 cm^−1^. HRMS (FAB, *m*/*z*) calcd for C_8_H_12_Cl_3_O_3_ [M + H]^+^: 260.9852; found: 260.9854.

#### 3.2.12. *6,6,6-Trichloro-5-oxo-3-phenyl-hexanoic Acid Methyl Ester* (**3c**)

Colorless oil. ^1^H NMR (400 MHz, CDCl_3_): *δ* 2.71 (1H, dd, *J* = 16.0, 7.3 Hz), 2.80 (1H, dd, *J* = 16.0, 7.3 Hz), 3.41 (2H, d, *J* = 7.3 Hz), 3.61 (3H, s), 3.80 (1H, quin, *J* = 7.4 Hz), 7.20–7.33 (5H, m) ppm. ^13^C NMR (100 MHz, CDCl_3_): *δ* 37.0, 40.1, 40.2, 51.7, 55.2, 96.2, 114.0, 128.4, 133.6, 158.6, 171.9, 188.4 ppm. IR (ATR, KBr): 3474, 3447, 3085, 3063, 3031, 3008, 2956, 2933, 2905, 2848, 1968, 1953, 1889, 1731, 1693, 1603, 1583, 1496, 1455, 1438, 1425, 1378, 1362, 1258, 1218, 1166, 1102, 1077, 989, 918, 903, 876, 824, 764, 700, 610, 557, 542 cm^−1^. HRMS (EI, *m*/*z*) calcd for C_14_H_13_Cl_3_O_3_ [M]^+^: 321.9930; found: 321.9928.

#### 3.2.13. *6,6,6-Trichloro-3-(4-methoxy-phenyl)-5-oxo-hexanoic Acid Methyl Ester* (**3d**)

Colorless oil. ^1^H NMR (400 MHz, CDCl_3_): *δ* 2.68 (1H, dd, *J* = 15.9, 7.9 Hz), 2.75 (1H, dd, *J* = 16.0, 7.3 Hz), 3.36 (2H, d, *J* = 6.9 Hz), 3.61 (3H, s), 3.70–3.83 (1H, m), 6.83 (2H, d, *J* = 8.7), 7.17 (2H, d, *J* = 8.7) ppm. ^13^C NMR (100 MHz, CDCl_3_): *δ* 37.0, 40.1, 40.2, 51.7, 55.2, 96.2, 114.0, 128.4, 133.6, 158.6, 171.9, 188.4 ppm. IR (ATR, KBr): 3003, 2953, 2838, 1738, 1612, 1583, 1514, 1438, 1366, 1301, 1250, 1179, 1114, 1089, 1035, 874, 833, 747, 708, 549 cm^−1^. HRMS (EI, *m*/*z*) calcd for C_15_H_15_Cl_3_O_4_ [M]^+^: 352.0036; found: 352.0037.

#### 3.2.14. *6,6,6-Trichloro-3,3-dimethyl-oxo-hexanoic Acid Methyl Ester* (**3e**)

Colorless oil. ^1^H NMR (400 MHz, CDCl_3_): *δ* 1.17 (6H, s), 2.58 (2H, s), 3.23 (2H, s), 3.66 (3H, s) ppm. ^13^C NMR (100 MHz, CDCl_3_): *δ* 27.7, 32.8, 42.7, 44.1, 51.3, 96.7, 172.3, 189.2 ppm. IR (ATR, KBr): 3672, 3477, 3241, 2953, 2844, 2629, 2021, 1739, 1437, 1392, 1352, 1227, 1076, 1011, 881, 833, 748, 697, 652, 626, 560 cm^−1^. HRMS (EI, *m*/*z*) calcd for C_9_H_14_Cl_3_O_3_ [M + H]^+^: 275.0008; found: 275.0016.

#### 3.2.15. *6,6,6-Trichloro-3-methyl-5-oxo-3-phenyl-hexanoic Acid Methyl Ester* (**3f**)

Colorless oil. ^1^H NMR (400 MHz, CDCl_3_): *δ* 1.63 (3H, s), 2.91 (1H, d, *J* = 15.6 Hz), 3.05 (1H, d, *J* = 15.1 Hz), 3.58 (3H, s), 3.71 (1H, d, *J* = 18.8 Hz), 3.77 (1H, d, *J* = 18.8 Hz), 7.20–7.25 (1H, m), 7.30–7.38 (4H, m) ppm. ^13^C NMR (100 MHz, CDCl_3_): *δ* 25.7, 39.1, 42.9, 44.7, 51.5, 96.5, 125.3, 126.6, 128.5, 145.2, 171.8, 188.2 ppm. IR (ATR, KBr): *ν* 3670, 3489, 3091, 3060, 3026, 2952, 2843, 2602, 2017, 1949, 1873, 1731, 1602, 1583, 1498, 1436, 1383, 1347, 1277, 1206, 1082, 1013, 958, 874, 821, 758, 698, 632, 551, 535 cm^−1^.

#### 3.2.16. *6,6,6-Trichloro-3-methyl-5-oxo-3-p-tolyl-hexanoic Acid Methyl Ester* (**3g**)

Colorless oil. ^1^H NMR (400 MHz, CDCl_3_): *δ* 1.60 (3H, s), 2.31 (3H, s), 2.94 (1H, d, *J* = 15.1 Hz), 3.03 (1H, d, *J* = 15.1 Hz), 3.59 (3H, s), 3.64 (1H, d, *J* = 18.8 Hz), 3.74 (1H, d, *J* = 18.8 Hz), 7.13 (2H, d, *J* = 8.2 Hz), 7.23 (2H, d, *J* = 8.2 Hz) ppm. ^13^C NMR (100 MHz, CDCl_3_): *δ* 20.9, 25.8, 38.8, 42.9, 44.7, 51.5, 96.6, 125.2, 129.2, 136.2, 142.3, 171.9, 188.2 ppm. IR (ATR, KBr): *ν* 3508, 2952, 1901, 1735, 1517, 1436, 1417, 1381, 1347, 1277, 1204, 1097, 1077, 1013, 959, 877, 858, 816, 751, 708, 681, 661, 630, 602, 593, 551 cm^−1^. HRMS (EI, *m*/*z*) calcd for C_15_H_17_Cl_3_O_3_ [M]^+^: 320.0243; found: 320.0244.

#### 3.2.17. General Procedure for Synthesis of Dichloroketo Acid Alkyl Esters

To a solution of a 1,3-cyclohexadione (1.0 mmol) and NCS (267.1 mg, 2.0 mmol) in alcohol (10 mL), Na_2_HPO_4_ (71.0 mg, 0.5 mmol) was added. After stirring the mixture at room temperature for 0.5 h, the solvent was evaporated. The product was dissolved in CH_2_Cl_2_, and the extract was filtered through filter paper for dehydration. After evaporating the solvent, the product was dissolved in hexane via sonication, and the extract was filtered through filter paper. The filtrate was concentrated by evaporation and purified by silica gel chromatography using an ethyl acetate (AcOEt)/hexane solution.

#### 3.2.18. *6,6-Dichloro-5-oxo-hexanoic Acid Ethyl Ester* (**4**)

Colorless oil. ^1^H NMR (400 MHz, CDCl_3_): *δ* 1.26 (3H, t, *J* = 7.3 Hz), 2.00 (2H, quin, *J* = 7.3 Hz), 2.38 (2H, t, *J* = 7.3 Hz), 2.92 (2H, t, *J* = 7.3 Hz), 4.14 (2H, q, *J* = 7.3 Hz), 5.83 (1H, s) ppm ^13^C NMR (100 MHz, CDCl_3_): *δ* 4.2, 18.9, 32.9, 33.8, 60.5, 69.8, 172.8, 196.7 ppm. IR (ATR, KBr): *ν* 3643, 3451, 2983, 1731, 1448, 1376, 1190, 1094, 1029, 858, 789, 735 cm^−1^. HRMS (EI, *m*/*z*) calcd for C_8_H_13_Cl_2_O_3_ [M + H]^+^: 227.0242; found: 227.0244.

#### 3.2.19. *6,6-Dichloro-5-oxo-hexanoic Acid Propyl Ester* (**5**)

Colorless oil. ^1^H NMR (400 MHz, CDCl_3_): *δ* 0.94 (3H, t, *J* = 7.5 Hz), 1.65 (3H, sext, *J* = 7.3 Hz), 2.00 (2H, quin, *J* = 7.3 Hz), 2.39 (2H, t, *J* = 7.3 Hz), 2.93 (2H, t, *J* = 7.1 Hz), 4.05 (2H, t, *J* = 6.9 Hz), 5.83 (1H, s) ppm. ^13^C NMR (100 MHz, CDCl_3_): *δ* 10.4, 18.9, 21.9, 32.8, 33.8, 66.2, 69.8, 172.9, 196.7 ppm. IR (ATR, KBr): *ν* 3446, 2969, 2880, 1732, 1459, 1395, 1379, 1314, 1185, 1089, 1061, 1002, 912, 849, 788, 736 cm^−1^. HRMS (EI, *m*/*z*) calcd for C_9_H_14_Cl_2_O_3_ [M + H]^+^: 241.0398; found: 241.0400.

#### 3.2.20. *6,6-Dichloro-5-oxo-hexanoic Acid 2-Methoxy-Ethyl Ester* (**6**)

Colorless oil. ^1^H NMR (400 MHz, CDCl_3_): *δ* 2.01 (2H, q, *J* = 7.3 Hz), 2.44 (2H, t, *J* = 7.3 Hz), 2.93 (2H, t, *J* = 7.1 Hz), 3.39 (3H, s), 3.58–3.61 (2H, m), 4.24–4.26 (2H, m), 5.84 (1H, s) ppm. ^13^C NMR (100 MHz, CDCl_3_): *δ* 18.9, 32.7, 33.8, 59.0, 63.6, 69.8, 70.4, 172.8, 196.7 ppm. ^1^ IR (ATR, KBr): *ν* 3632, 3449, 2936, 1732, 1452, 1407, 1380, 1127, 1033, 861, 784, 734, 534 cm^−1^. HRMS (EI, *m*/*z*) calcd for C_9_H_14_Cl_2_O_4_ [M + H]^+^: 257.0347; found: 257.0344.

#### 3.2.21. *6,6-Dichloro-5-oxo-hexanoic Acid Butyl Ester* (**7**)

Colorless oil. ^1^H NMR (400 MHz, CDCl_3_): *δ* 0.94 (3H, t, *J* = 7.5 Hz), 1.38 (2H, sext, *J* = 7.6 Hz), 1.61 (2H, quin, *J* = 7.2 Hz), 2.00 (2H, quin, *J* = 7.3 Hz), 2.39 (2H, t, *J* = 7.3 Hz), 2.92 (2H, t, *J* = 7.1 Hz), 4.09 (2H, t, *J* = 6.9 Hz), 5.83 (1H, s) ppm. ^13^C NMR (100 MHz, CDCl_3_): *δ* 13.7, 18.9, 19.1, 30.6, 32.9, 33.9, 64.5, 69.8, 172.9, 196.7 ppm. IR (ATR, KBr): *ν* 3620, 3449, 2961, 2874, 1732, 1457, 1394, 1314, 1188, 1063, 1024, 966, 845, 788, 731, 518 cm^−1^. HRMS (EI, *m*/*z*) calcd for C_10_H_16_Cl_2_O_3_ [M + H]^+^: 255.0555; found: 255.0552.

#### 3.2.22. *6,6-Dichloro-5-oxo-hexanoic Acid Isobutyl Ester* (**8**)

Colorless oil. ^1^H NMR (400 MHz, CDCl_3_): *δ* 0.93 (6H, d, *J* = 6.8 Hz), 1.88–1.98 (1H, m), 2.00 (2H, quin, *J* = 7.3 Hz), 2.40 (2H, t, *J* = 7.3 Hz), 2.93 (2H, t, *J* = 7.1 Hz), 3.87 (2H, d, *J* = 6.9 Hz), 5.83 (1H, s) ppm. ^13^C NMR (100 MHz, CDCl_3_): *δ* 18.9, 19.1, 27.7, 32.8, 33.9, 69.8, 70.7, 172.9, 196.7 ppm. IR (ATR, KBr): *ν* 3650, 3450, 2961, 2875, 1732, 1470, 1382, 1189, 1088, 1014, 946, 782, 728, 521 cm^−1^. HRMS (EI, *m*/*z*) calcd for C_10_H_16_Cl_2_O_3_ [M + H]^+^: 255.0555; found: 255.0552.

#### 3.2.23. *7,7-Dichloro-5-oxo-heptanoic Acid Methyl Ester* (**10**)

Colorless oil. ^1^H NMR (500 MHz, CDCl_3_): *δ* 1.67–1.72 (4H, m), 2.36 (2H, t, *J* = 6.9 Hz), 2.86 (2H, t, *J* = 6.9 Hz), 3.68 (3H, s), 5.84 (1H, s) ppm. ^13^C NMR (125 MHz, CDCl_3_): *δ* 23.1, 24.1, 33.6, 34.5, 51.6, 69.8, 173.6, 196.9 ppm. HRMS (EI, *m*/*z*) calcd for C_8_H_13_Cl_2_O_3_ [M + H]^+^: 227.0242; found: 227.0243.

#### 3.2.24. *8,8-Dichloro-5-oxo-octanoic Acid Methyl Ester* (**12**)

Colorless oil. ^1^H NMR (500 MHz, CDCl_3_): *δ* 1.35–1.41 (2H, m), 1.63–1.72 (4H, m), 2.33 (2H, t, *J* = 7.5 Hz), 2.83 (2H, t, *J* = 7.2 Hz), 3.67 (3H, s), 5.82 (1H, s) ppm. ^13^C NMR (125 MHz, CDCl_3_): *δ* 23.4, 24.5, 28.3, 33.8, 34.6, 51.5, 69.9, 174.0, 197.1 ppm. HRMS (EI, *m*/*z*) calcd for C_9_H_15_Cl_2_O_3_ [M + H]^+^: 241.0398; found: 241.0404.

#### 3.2.25. *8,8,8-Trichloro-5-oxo-octanoic Acid Methyl Ester* (**13**)

Colorless oil. ^1^H NMR (500 MHz, CDCl_3_): *δ* 1.41 (2H, quin, *J* = 7.7 Hz), 1.68 (2H, quin, *J* = 7.6 Hz), 1.77 (2H, quin, *J* = 7.4 Hz), 2.34 (2H, t, *J* = 7.5 Hz), 3.00 (2H, t, *J* = 7.5 Hz), 3.68 (3H, s) ppm. ^13^C NMR (125 MHz, CDCl_3_): *δ* 24.4, 24.5, 28.3, 33.66, 33.75, 51.6, 96.4, 173.9, 190.5 ppm.

#### 3.2.26. *5,5-Dichloro-5-oxo-pentanoic Acid Methyl Ester* (**15**)

Colorless oil. ^1^H NMR (500 MHz, CDCl_3_): *δ* 2.70 (2H, t, *J* = 6.6 Hz), 3.16 (2H, t, *J* = 6.6 Hz), 3.70 (3H, s), 5.93 (1H, s) ppm. ^13^C NMR (125 MHz, CDCl_3_): *δ* 27.9, 30.2, 52.0, 69.7, 172.4, 195.9 ppm. HRMS (EI, *m*/*z*) calcd for C_6_H_9_Cl_2_O_3_ [M + H]^+^: 198.9929; found: 198.9929.

#### 3.2.27. *6-Bromo-6,6-dichloro-5-oxo-hexanoic Acid Methyl Ester* (**16**)

Colorless oil. ^1^H NMR (500 MHz, CDCl_3_): *δ* 2.06 (2H, quin, *J* = 7.2 Hz), 2.42 (2H, t, *J* = 7.5 Hz), 3.14 (2H, t, *J* = 7.2 Hz), 3.67 (3H, s) ppm. ^13^C NMR (125 MHz, CDCl_3_): *δ* 20.1, 32.4, 32.8, 51.7, 80.8, 173.1, 190.4 ppm. HRMS (EI, *m*/*z*) calcd for C_7_H_10_BrCl_2_O_3_ [M + H]^+^: 290.9190; found: 290.9194.

## 4. Conclusions

In this study, we identified a unique reaction system that facilitates the direct synthesis of di- and trichloroketoesters from cyclic 1,3-diketones at ambient temperatures. The use of Na_2_HPO_4_, which plays a key role in the alcoholytic ring-opening reactions of the 2,2-dichloro-1,3-diketones generated as intermediates, was demonstrated to yield haloketo acid methyl esters with high efficiency. The selective synthesis of dichloro and trichloro products can be modulated by the stoichiometry of the NCS. Although the mechanism by which Na_2_HPO_4_ influences C–C bond cleavage remains unclear, this method has significant potential for further derivatization. The integration of this method with various heterocycle formation techniques utilizing haloketo acids and their esters is anticipated to aid in the synthesis of diverse new compounds functionalized with or without halogen atoms.

## Figures and Tables

**Table 1:** Effect of buffer salt on the halogenative ring-cleavage reaction.

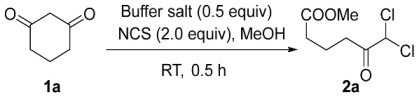		
Entry	Buffer Salt	Yield (%)
1	Na_2_HPO_4_	100
2	K_2_HPO_4_	80
3	NaH_2_PO_4_	60 (10) ^1^
4	KH_2_PO_4_	14 (21) ^1^
5	NaHCO_3_	91
6	KHCO_3_	82
7	Na_2_CO_3_	71
8	K_2_CO_3_	57
9	AcONa	95
10	AcOK	92

^1^ The numerals in parentheses are the yield of 2,2-dichloro-1,3-cyclohexadione.

**Table 2:** Substrate scope of the halogenative ring-cleavage reaction producing dichloroketoesters.

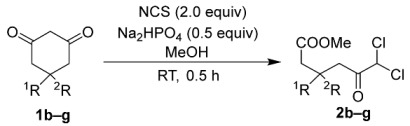		
Entry	Product	Yield (%)
1	**2b** (^1^R = H, ^2^R = Me)	98
2	**2c** (^1^R = H, ^2^R = Ph)	99
3	**2d** (^1^R = H, ^2^R = *p*-MeOC_6_H_4_)	87
4	**2e** (^1^R, ^2^R = Me)	97
5	**2f** (^1^R = Ph, ^2^R = Me)	93
6	**2g** (^1^R = *p*-Me C_6_H_4_, ^2^R = Me)	95

**Table 3:** Halogenative ring-cleavage reaction producing trichloroketoesters **3a**–**g**.

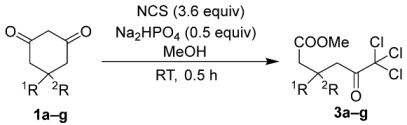		
Entry	Product	Yield (%)
1	**3a** (^1^R, ^2^R = H)	91
2	**3b** (^1^R = H, ^2^R = Me)	99
3	**3c** (^1^R = H, ^2^R = Ph)	90
4	**3d** (^1^R = H, ^2^R = *p*-MeOC_6_H_4_)	85
5	**3e** (^1^R, ^2^R = Me)	98
6	**3f** (^1^R = Ph, ^2^R = Me)	100
7	**3g** (^1^R = *p*-Me C_6_H_4_, ^2^R = Me)	98

**Table 4:** Synthesis of dichloroketo acid alkyl esters.

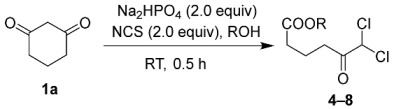		
Entry (Alcohol)	Product	Yield (%)
1 (EtOH)	**4** (R = Et)	86
2 (*^n^*PrOH)	**5** (R = *^n^*Pr)	77
3 (2-methoxyethanol)	**6** (R = CH_2_CH_2_OCH_3_)	78
4 (*^n^*BuOH)	**7** (R = *^n^*Bu)	40
5 (*^t^*BuOH)	**8** (R = *^t^*Bu)	27

## Data Availability

The data supporting this work is in the article and Supplementary Materials.

## Supplementary Materials

The following supporting information can be downloaded at https://www.mdpi.com/1420-3049/31/1/199/s1, ^1^H and ^13^C NMR spectra of compounds **2a−g**, **3a−g**, **4−8**, **10**, **12**, **13**, **15**, and **16**.
