# Supplementary material for: Chemoselectively Functionalized Ketoesters by Halogenative C–C Bond Cleavage of Cyclic Diketones
Source: Molecules. 2026 Jan 5;31(1):199. doi: 10.3390/molecules31010199 (PMC12787718; doi:10.3390/molecules31010199)
Supplement: Supplementary file 1 [file molecules-31-00199-s001.zip › molecules-4068948-supplementary.pdf]

Supporting Information

Chemoselectively Functionalized Ketoesters by Halogenative C–C Bond Cleavage of Cyclic Diketones

Hideyasu China, Nami Kageyama, Hodaka Yatabe, Mihoyo Fujitaka, Yusei Matsumoto, Jing Zhihan, and Toshifumi Dohi

1. ^{1}H and ^{13}C NMR spectra of Compound **2a** 2
2. ^{1}H and ^{13}C NMR spectra of Compound **2b** 3
3. ^{1}H and ^{13}C NMR spectra of Compound **2c** 4
4. ^{1}H and ^{13}C NMR spectra of Compound **2d** 5
5. ^{1}H and ^{13}C NMR spectra of Compound **2e** 6
6. ^{1}H and ^{13}C NMR spectra of Compound **2f** 7
7. ^{1}H and ^{13}C NMR spectra of Compound **2g** 8
8. ^{1}H and ^{13}C NMR spectra of Compound **3a** 9
9. ^{1}H and ^{13}C NMR spectra of Compound **3b** 10
10. ^{1}H and ^{13}C NMR spectra of Compound **3c** 11
11. ^{1}H and ^{13}C NMR spectra of Compound **3d** 12
12. ^{1}H and ^{13}C NMR spectra of Compound **3e** 13
13. ^{1}H and ^{13}C NMR spectra of Compound **3f** 14
14. ^{1}H and ^{13}C NMR spectra of Compound **3g** 15
15. ^{1}H and ^{13}C NMR spectra of Compound **4** 16
16. ^{1}H and ^{13}C NMR spectra of Compound **5** 17
17. ^{1}H and ^{13}C NMR spectra of Compound **6** 18
18. ^{1}H and ^{13}C NMR spectra of Compound **7** 19
19. ^{1}H and ^{13}C NMR spectra of Compound **8** 20
20. ^{1}H and ^{13}C NMR spectra of Compound **10** 21
21. ^{1}H and ^{13}C NMR spectra of Compound **12** 22
22. ^{1}H and ^{13}C NMR spectra of Compound **13** 23
23. ^{1}H and ^{13}C NMR spectra of Compound **15** 24
24. ^{1}H and ^{13}C NMR spectra of Compound **16** 25

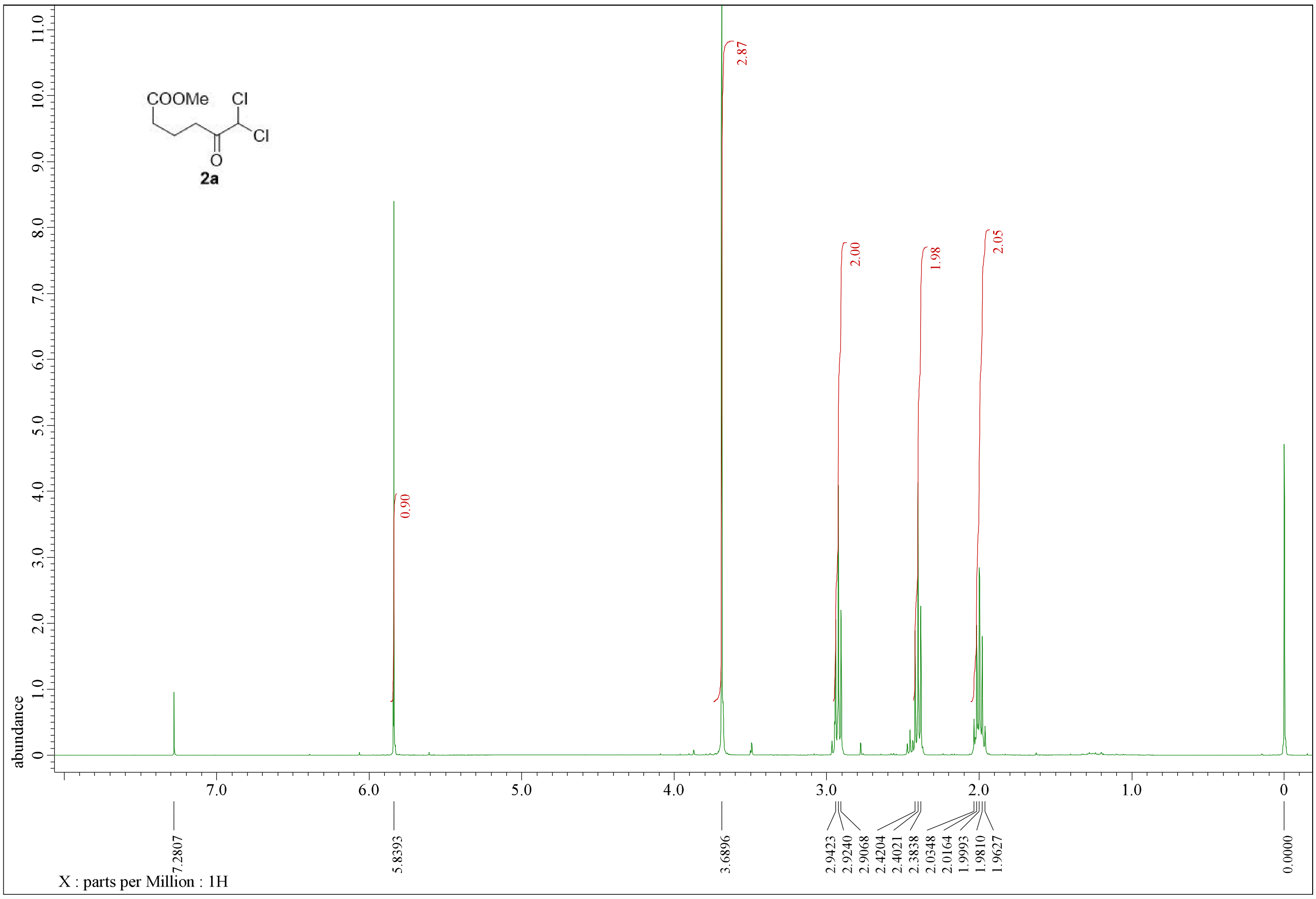

COOMe
Cl
Cl
O
2a
abundance
X : parts per Million : 1H
2.87
0.90
2.00
1.98
2.05
7.2807
5.8393
3.6896
2.9423
2.9240
2.9068
2.4204
2.4021
2.3838
2.0348
2.0164
1.9993
1.9810
1.9627
0.0000

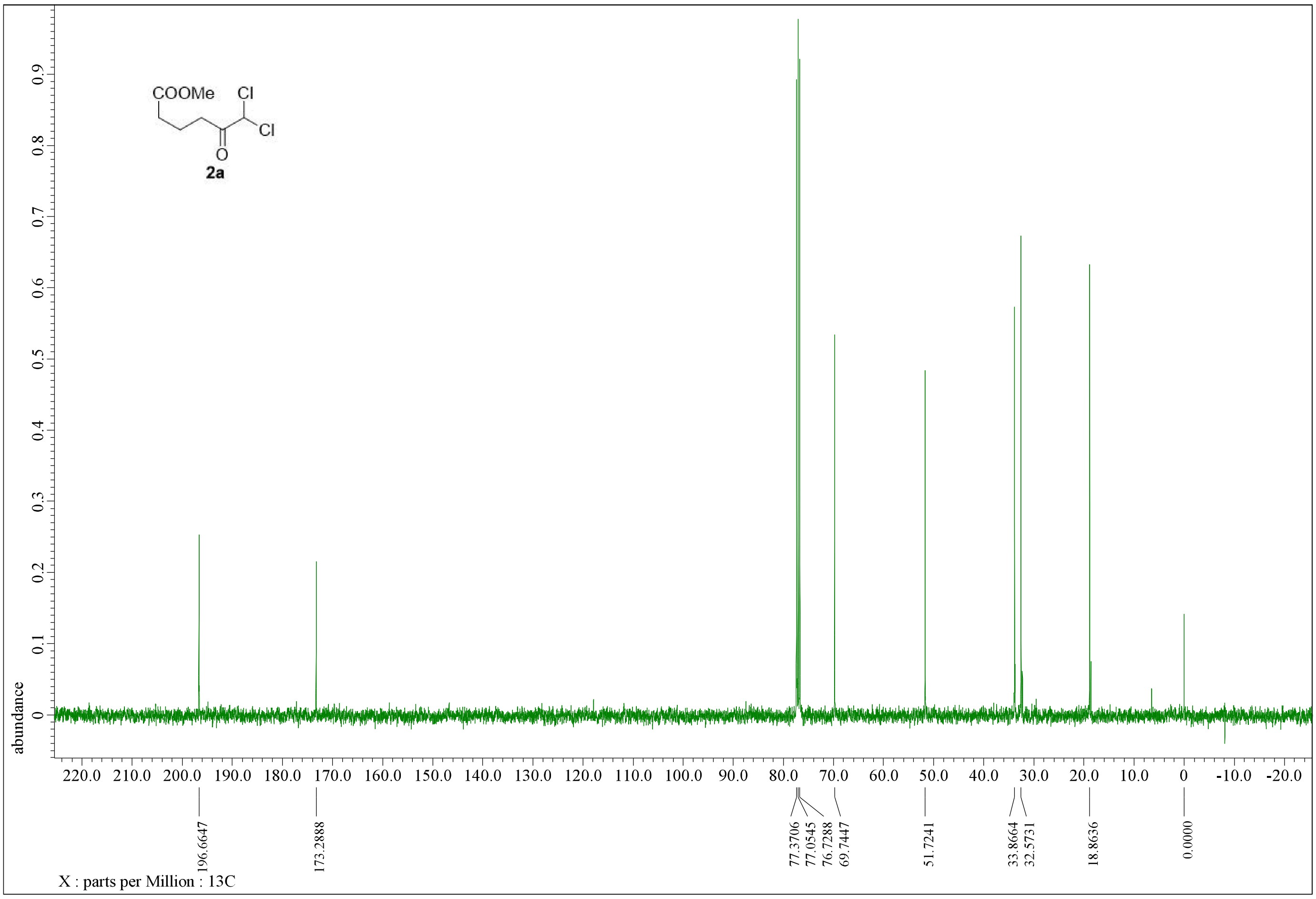

COOMe
Cl
Cl
O
2a
abundance
X : parts per Million : 13C
196.6647
173.2888
77.3706
77.0545
76.7288
69.7447
51.7241
33.8664
32.5731
18.8636
0.0000

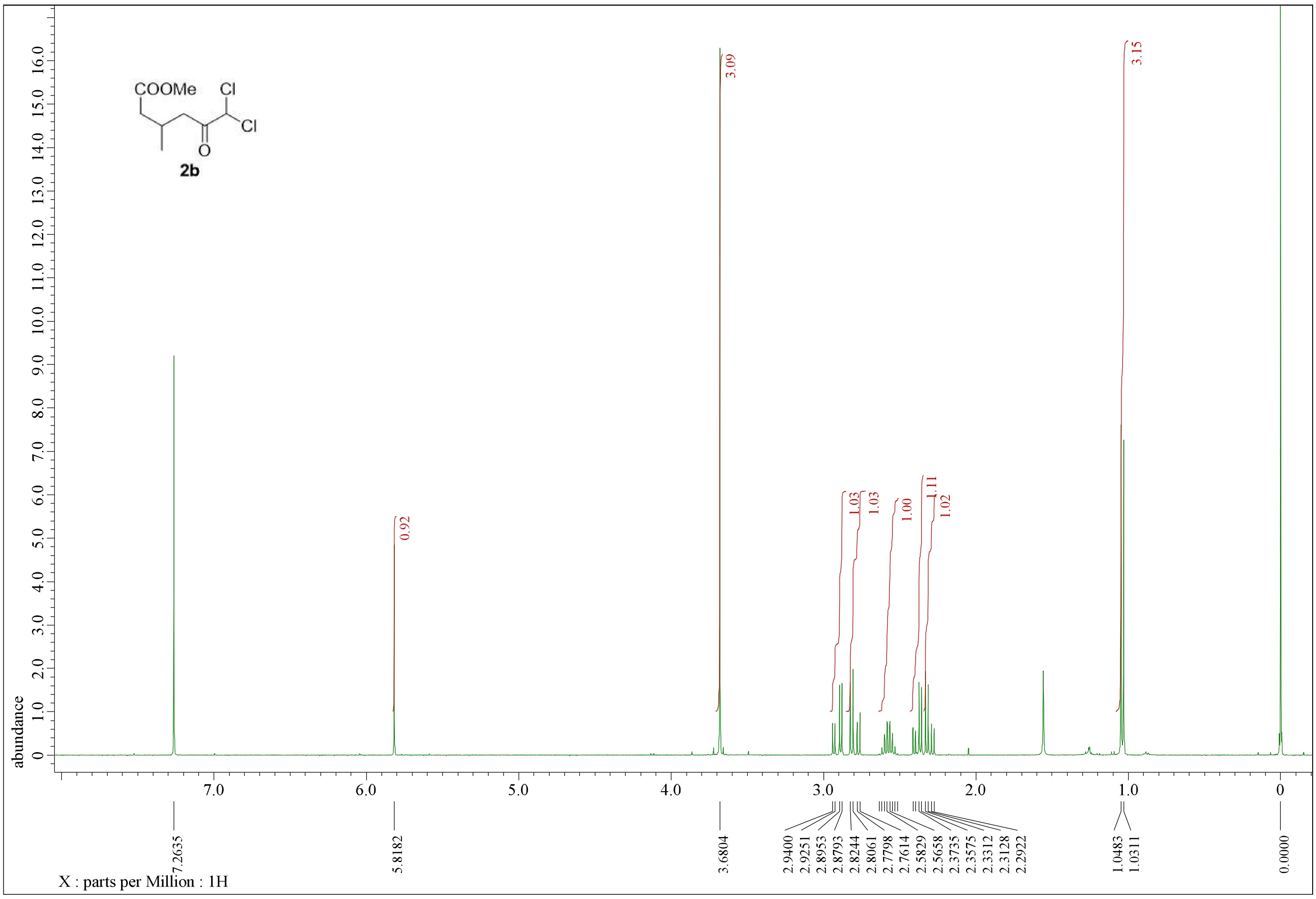
COOMe
Cl
Cl
O
2b
abundance
X : parts per Million : 1H
7.2635
5.8182
3.6804
2.9400
2.9251
2.8953
2.8793
2.8244
2.8061
2.7798
2.7614
2.5829
2.5658
2.3735
2.3575
2.3312
2.3128
2.2922
1.0483
1.0311
0.0000
0.92
3.09
1.03
1.03
1.00
1.11
1.02
3.15

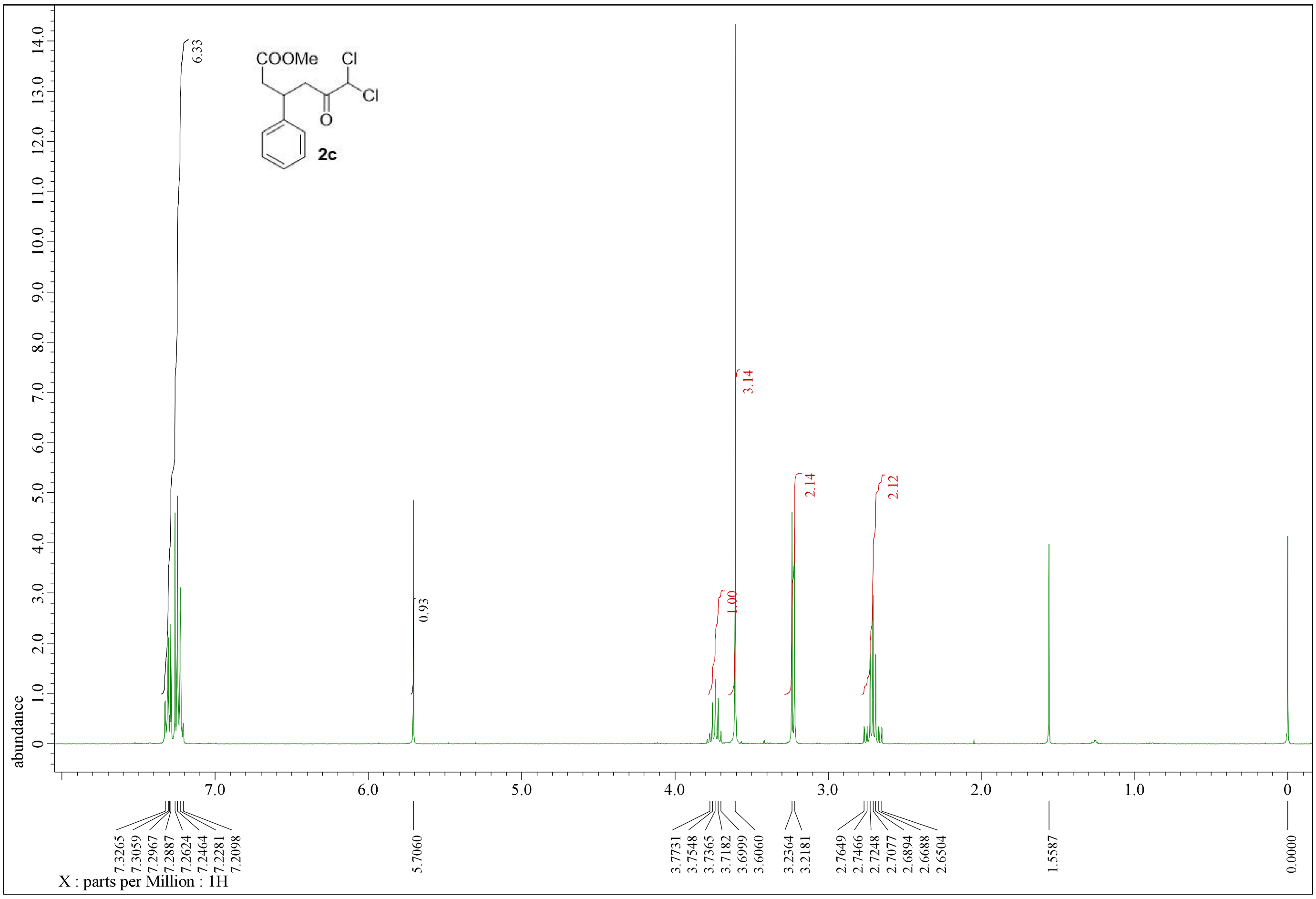
COOMe
Cl
Cl
O
2c
abundance
X : parts per Million : 1H
7.3265
7.3059
7.2967
7.2887
7.2624
7.2464
7.2281
7.2098
5.7060
3.7731
3.7548
3.7365
3.7182
3.6999
3.6060
3.2364
3.2181
2.7649
2.7466
2.7248
2.7077
2.6894
2.6688
2.6504
1.5587
0.0000
6.33
0.93
1.00
3.14
2.14
2.12

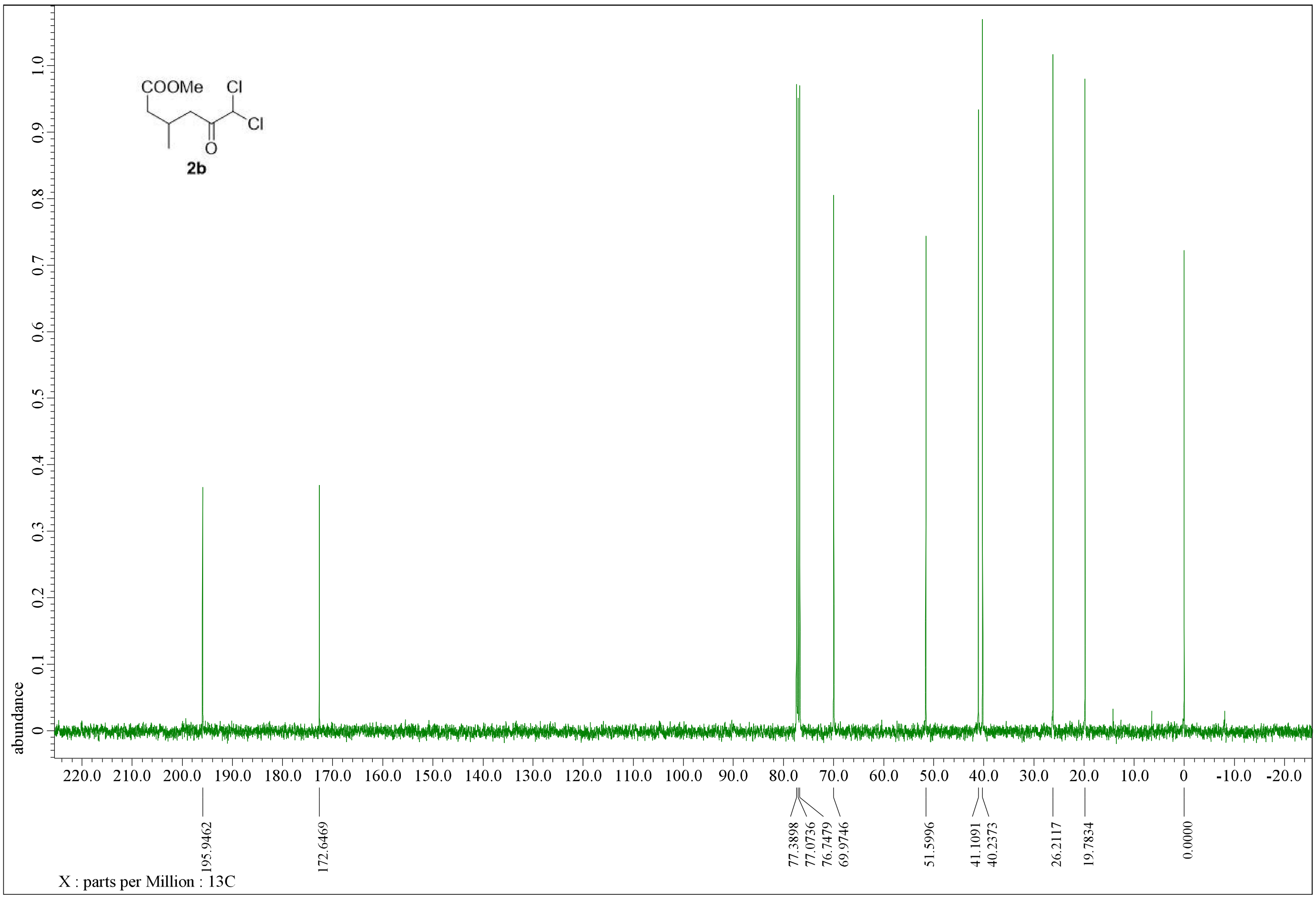
COOMe
Cl
Cl
O
2b
abundance
X : parts per Million : 13C
195.9462
172.6469
77.3898
77.0736
76.7479
69.9746
51.5996
41.1091
40.2373
26.2117
19.7834
0.0000

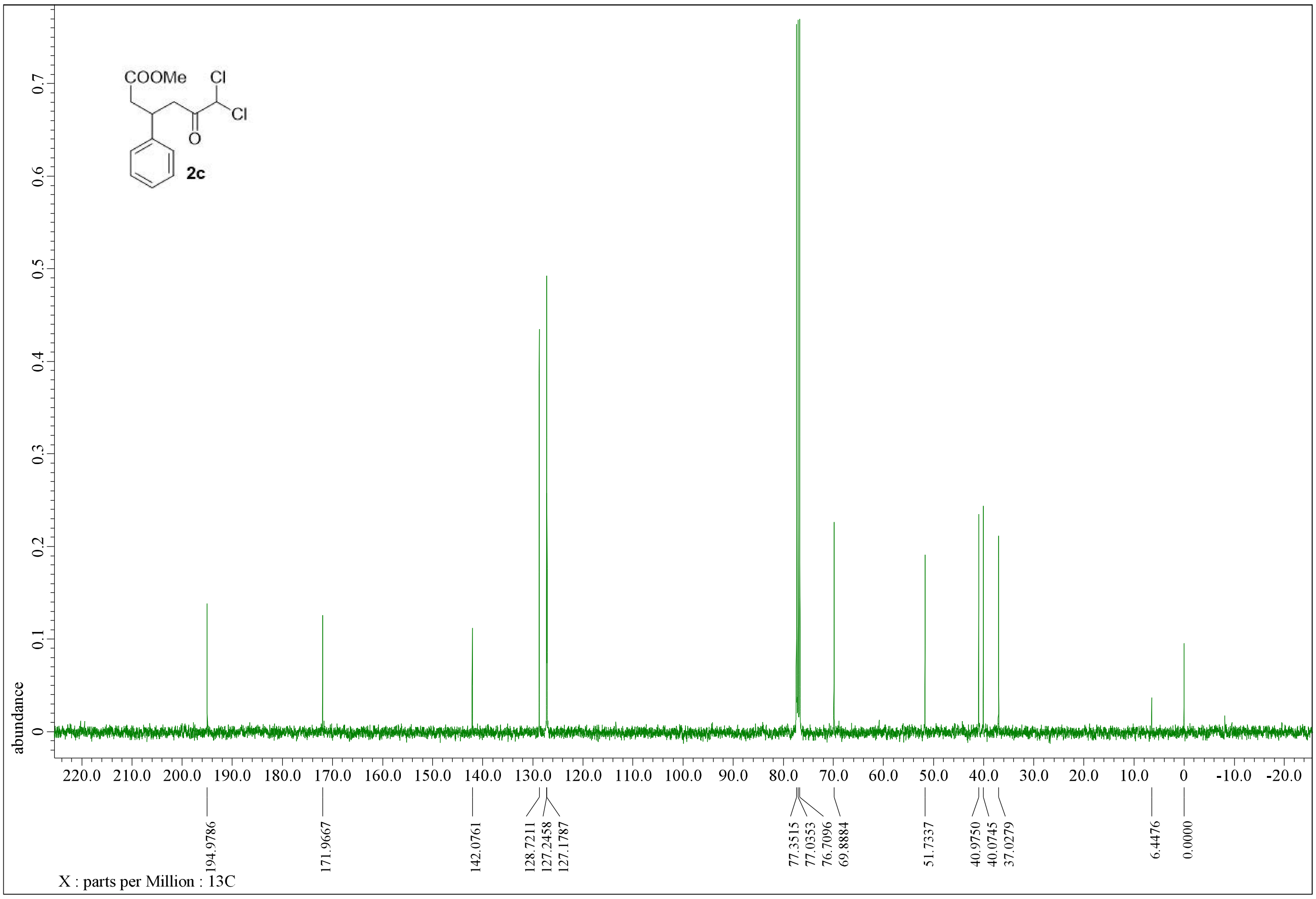
COOMe
Cl
Cl
O
2c
abundance
X : parts per Million : 13C
194.9786
171.9667
142.0761
128.7211
127.2458
127.1787
77.3515
77.0353
76.7096
69.8884
51.7337
40.9750
40.0745
37.0279
6.4476
0.0000

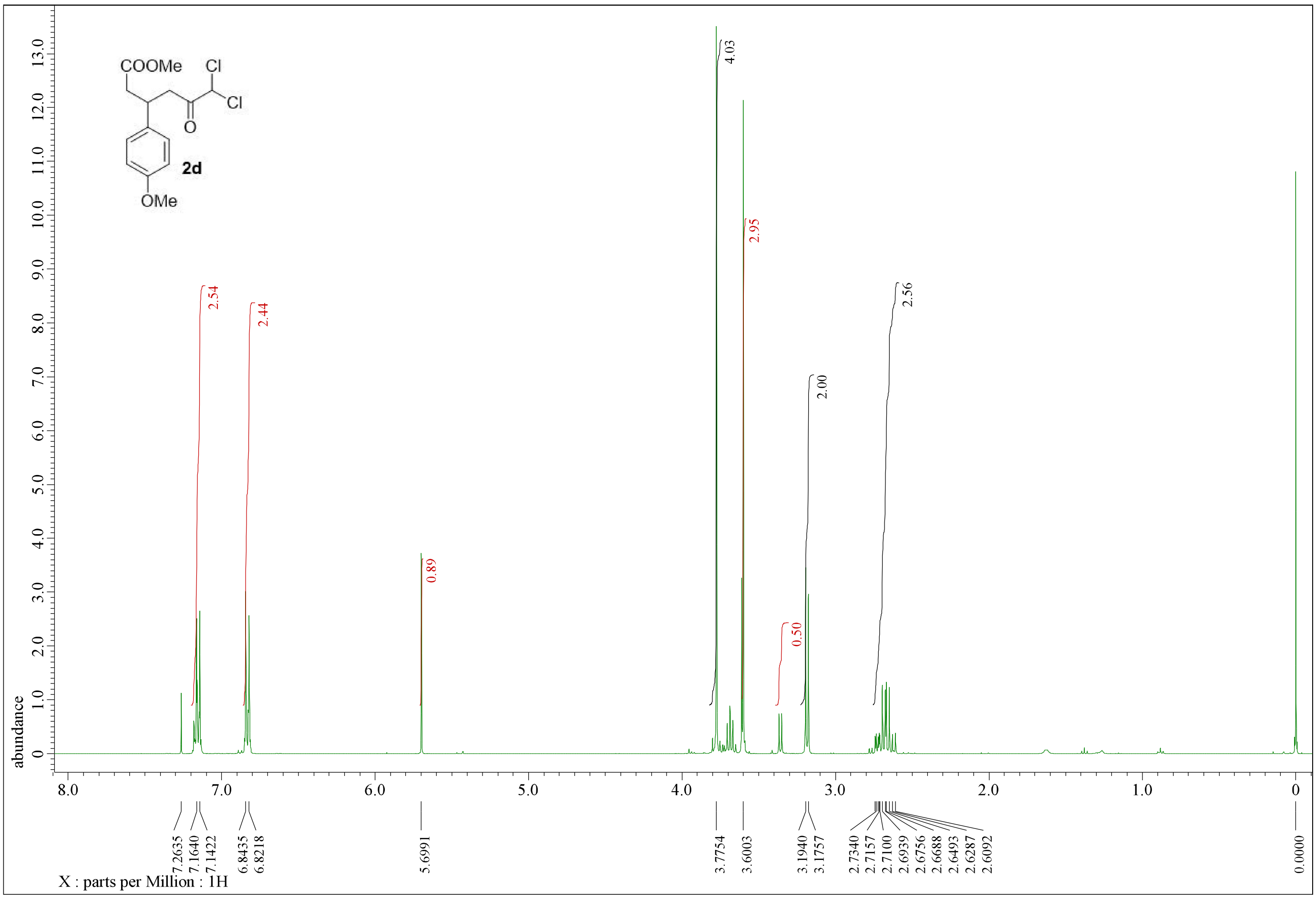
COOMe
Cl
Cl
O
2d
OMe
abundance
X : parts per Million : 1H
2.54
2.44
0.89
4.03
2.95
0.50
2.00
2.56
7.2635
7.1640
7.1422
6.8435
6.8218
5.6991
3.7754
3.6003
3.1940
3.1757
2.7340
2.7157
2.7100
2.6939
2.6756
2.6688
2.6493
2.6287
2.6092
0.0000

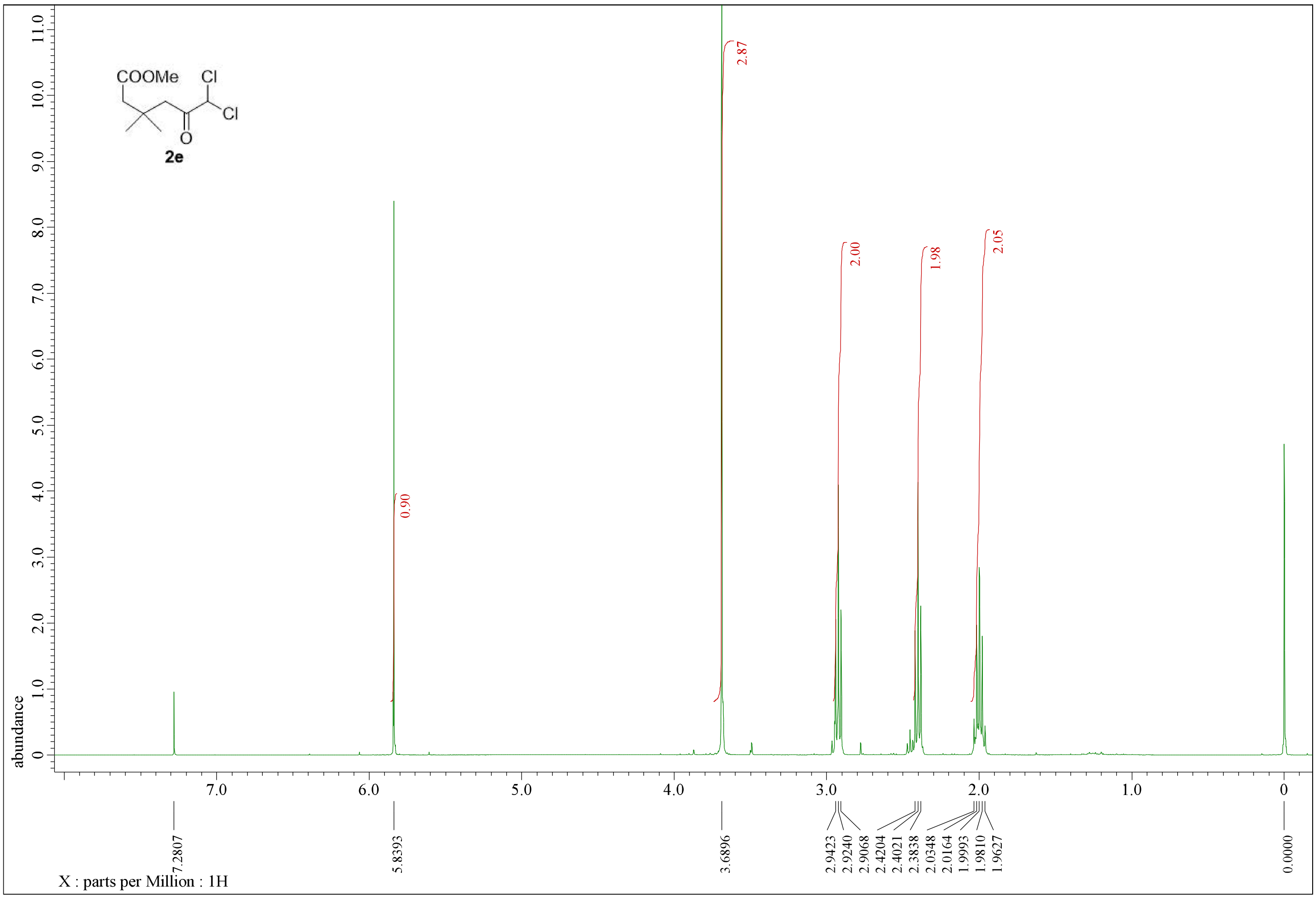
COOMe
Cl
Cl
O
2e
abundance
X : parts per Million : 1H
0.90
2.87
2.00
1.98
2.05
7.2807
5.8393
3.6896
2.9423
2.9240
2.9068
2.4204
2.4021
2.3838
2.0348
2.0164
1.9993
1.9810
1.9627
0.0000

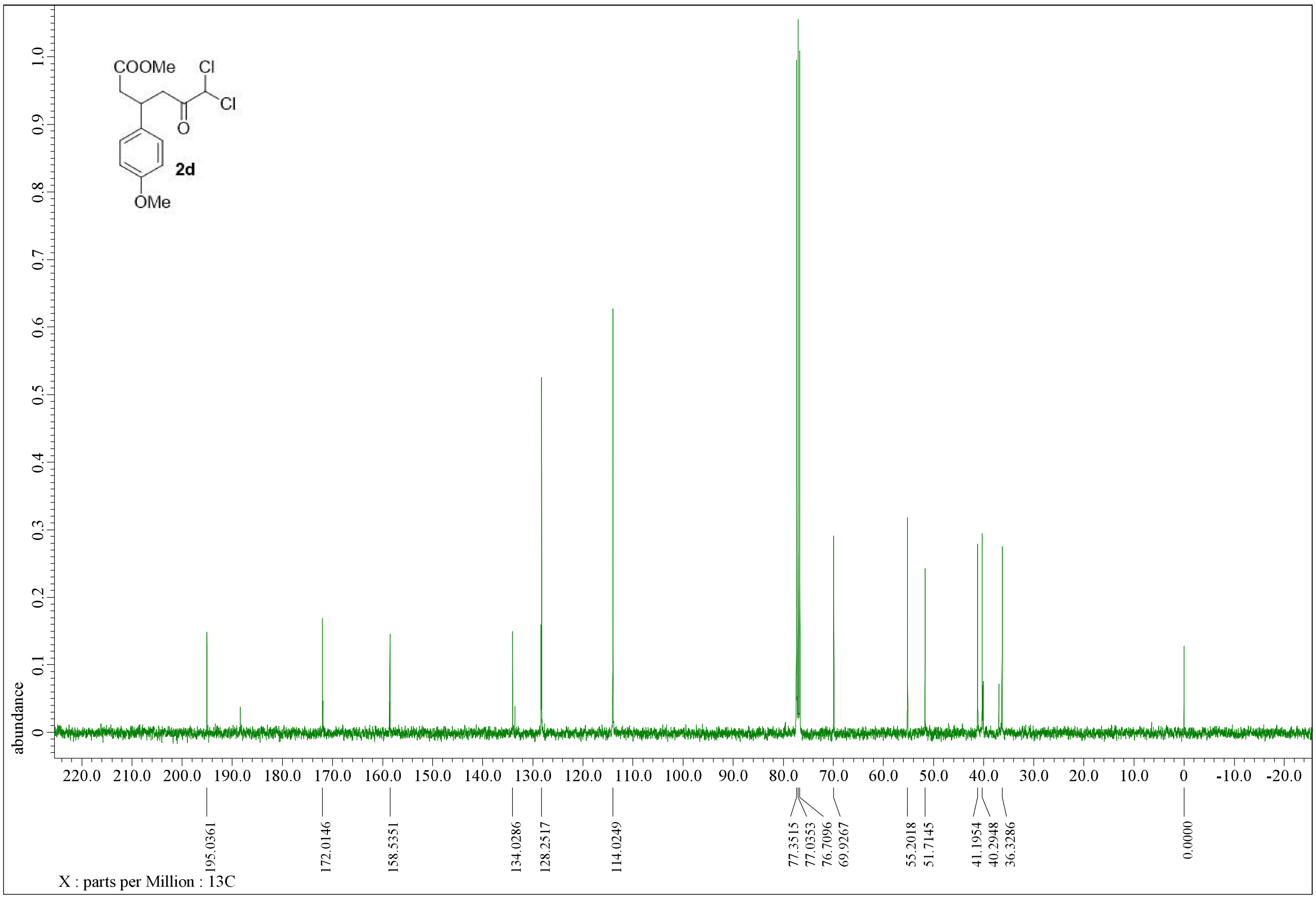
COOMe
Cl
Cl
O
2d
OMe
abundance
X : parts per Million : 13C
195.0361
172.0146
158.5351
134.0286
128.2517
114.0249
77.3515
77.0353
76.7096
69.9267
55.2018
51.7145
41.1954
40.2948
36.3286
0.0000

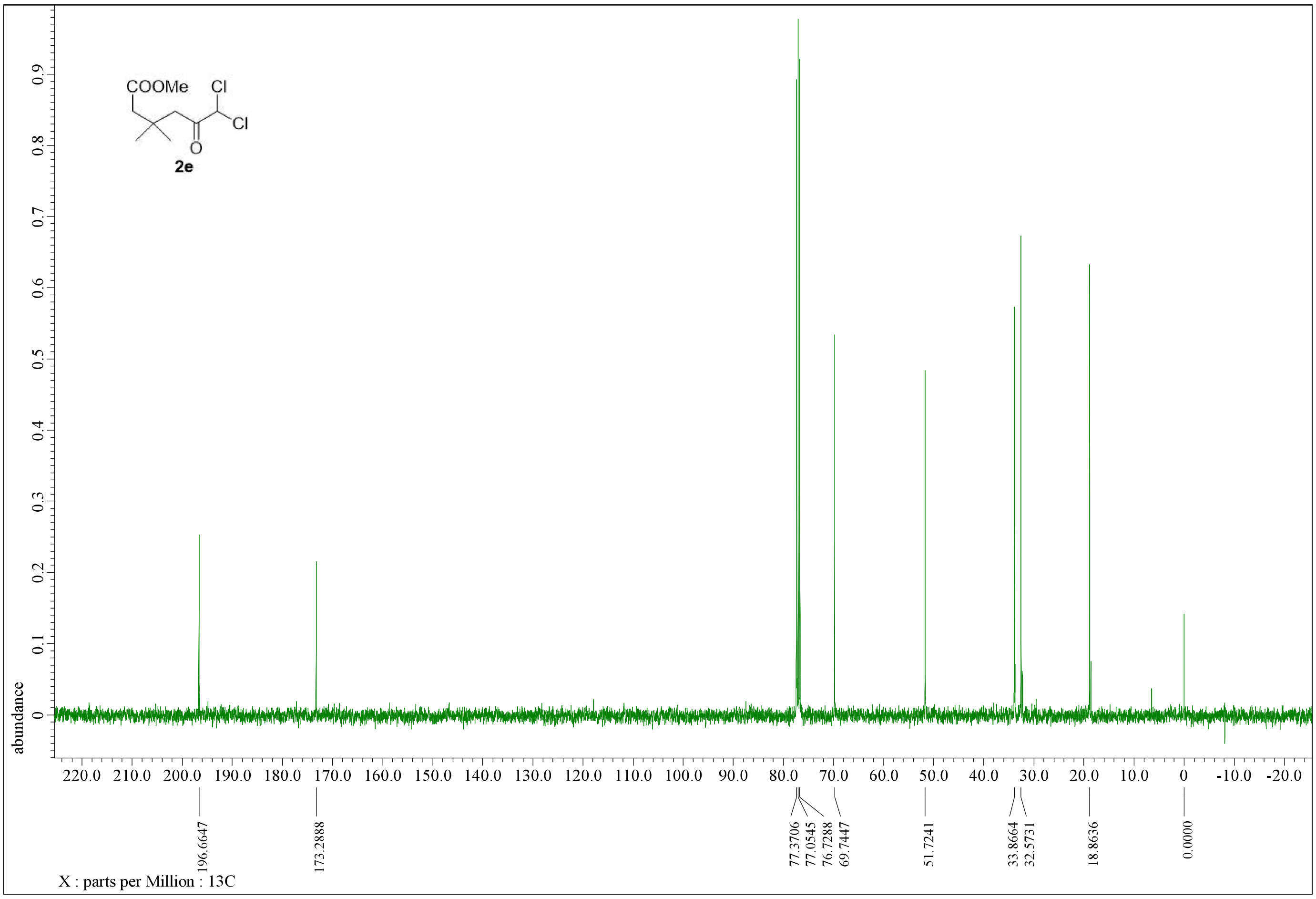
COOMe
Cl
Cl
O
2e
abundance
X : parts per Million : 13C
196.6647
173.2888
77.3706
77.0545
76.7288
69.7447
51.7241
33.8664
32.5731
18.8636
0.0000

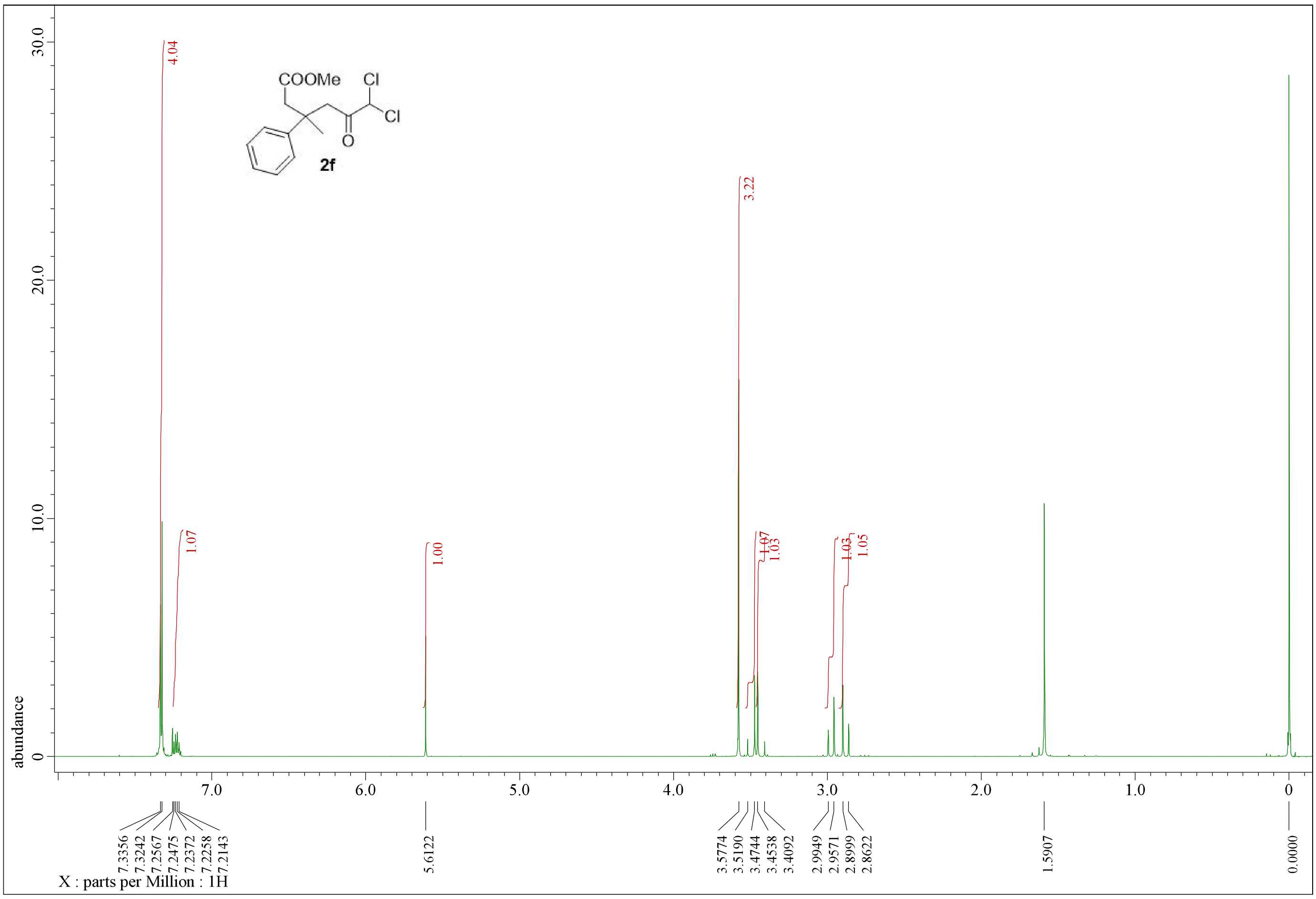
COOMe
Cl
Cl
O
2f
abundance
X : parts per Million : 1H
4.04
1.07
1.00
3.22
1.07
1.03
1.03
1.05
7.3356
7.3242
7.2567
7.2475
7.2372
7.2258
7.2143
5.6122
3.5774
3.5190
3.4744
3.4538
3.4092
2.9949
2.9571
2.8999
2.8622
1.5907
0.0000

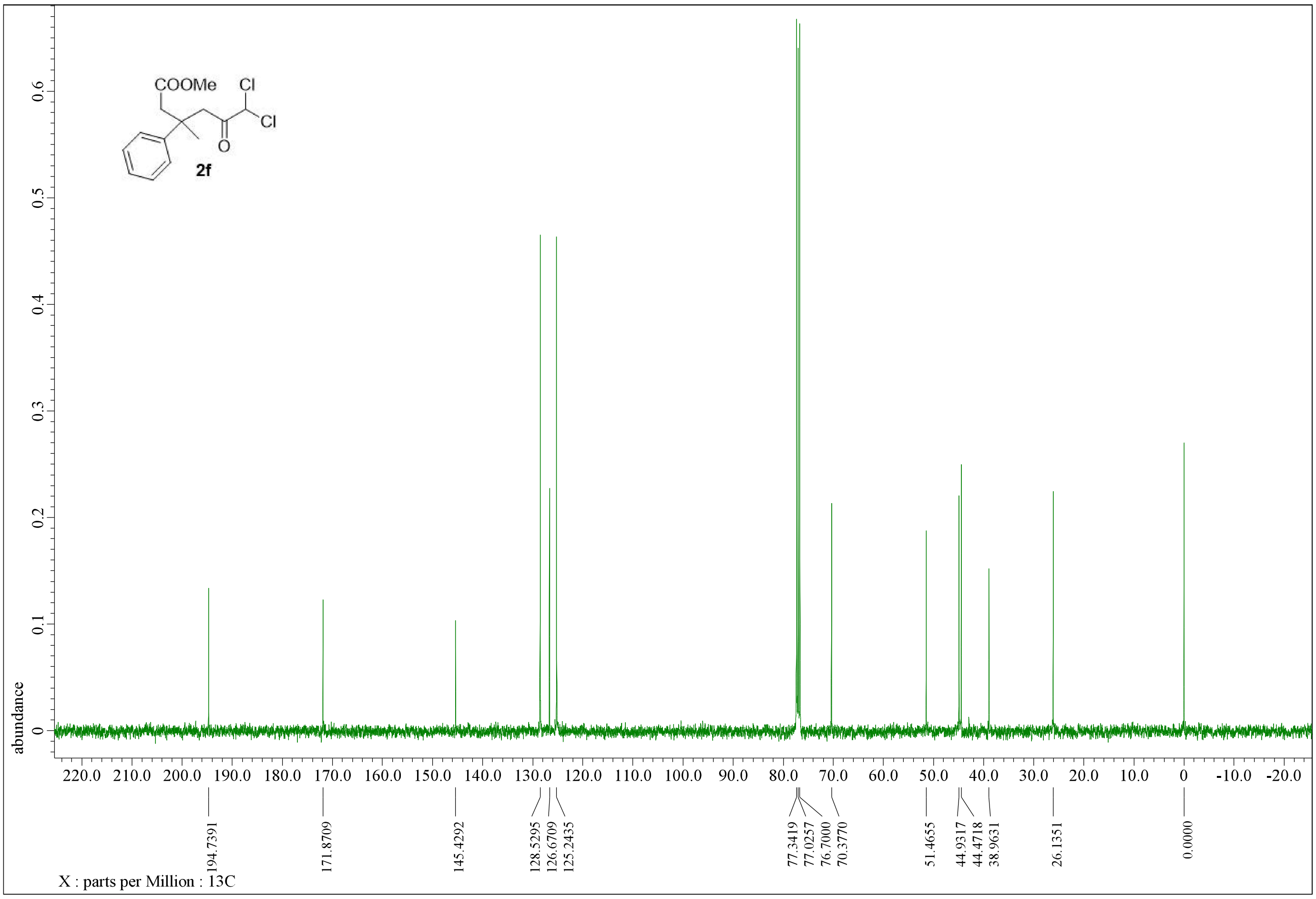
COOMe
Cl
Cl
O
2f
abundance
X : parts per Million : 13C
194.7391
171.8709
145.4292
128.5295
126.6709
125.2435
77.3419
77.0257
76.7000
70.3770
51.4655
44.9317
44.4718
38.9631
26.1351
0.0000

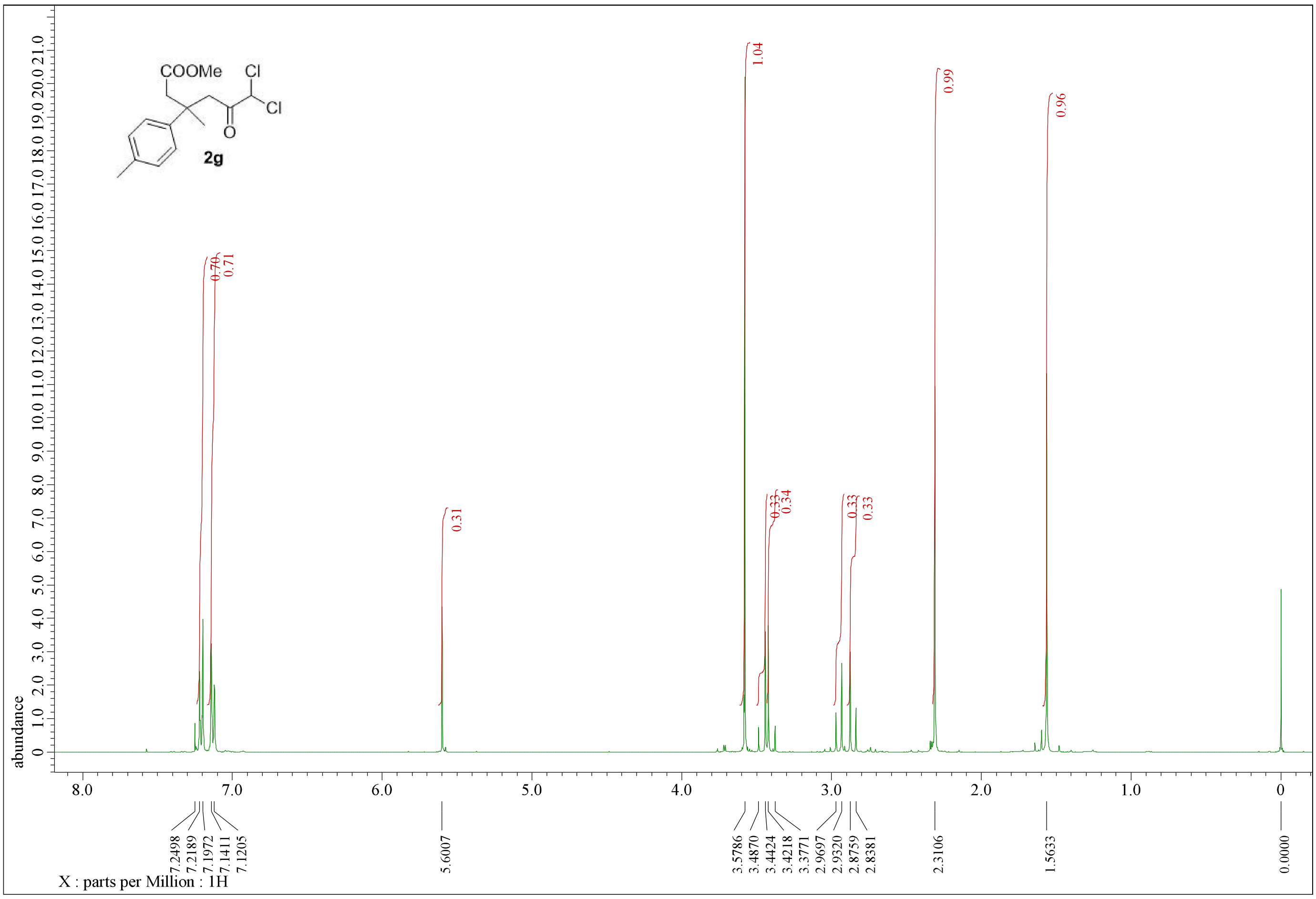
COOMe
Cl
Cl
O
2g
abundance
X : parts per Million : 1H
7.2498
7.2189
7.1972
7.1411
7.1205
5.6007
3.5786
3.4870
3.4424
3.4218
3.3771
2.9697
2.9320
2.8759
2.8381
2.3106
1.5633
0.0000
0.70
0.71
0.31
1.04
0.33
0.34
0.33
0.33
0.99
0.96

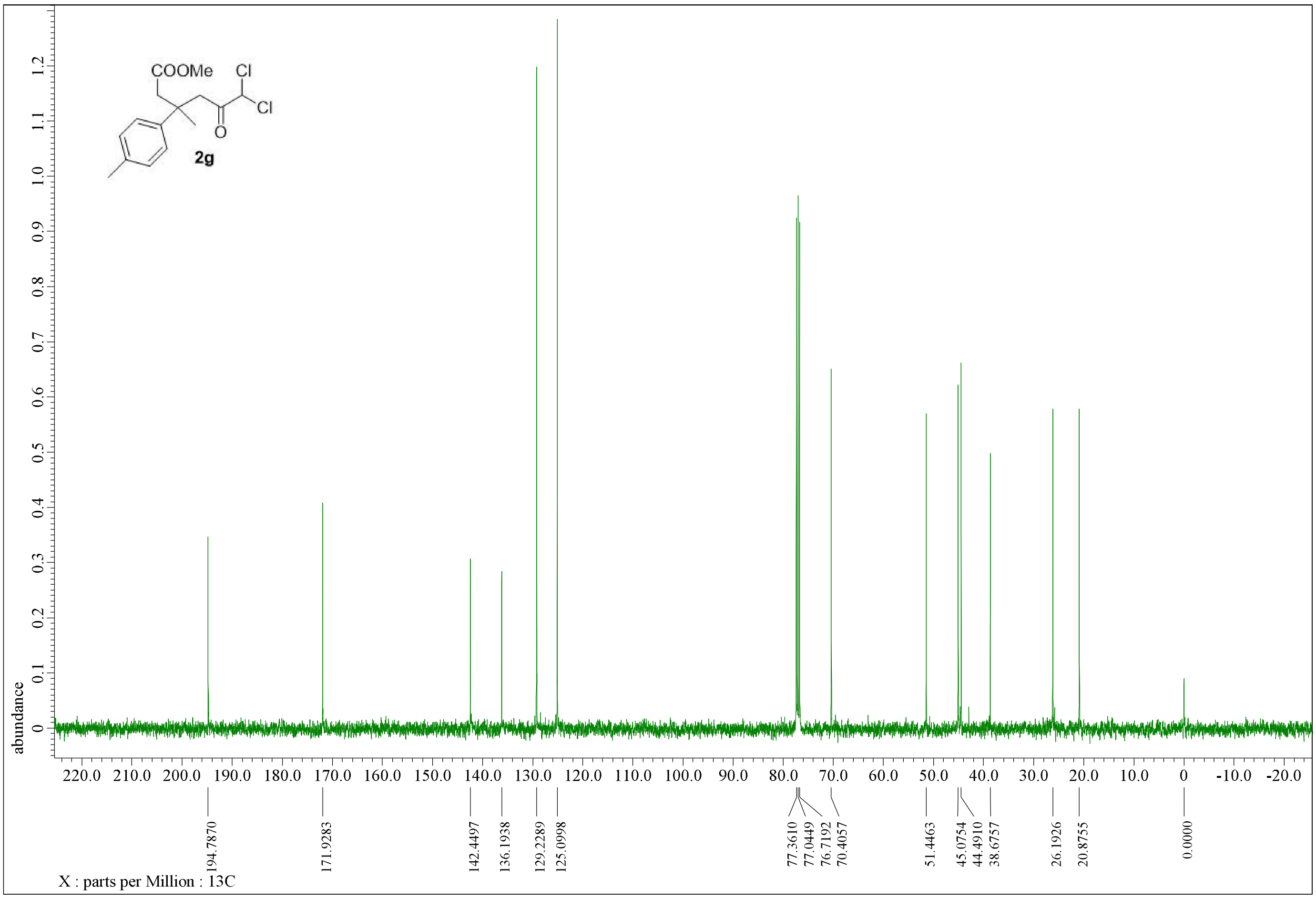
COOMe
Cl
Cl
O
2g
abundance
X : parts per Million : 13C
194.7870
171.9283
142.4497
136.1938
129.2289
125.0998
77.3610
77.0449
76.7192
70.4057
51.4463
45.0754
44.4910
38.6757
26.1926
20.8755
0.0000

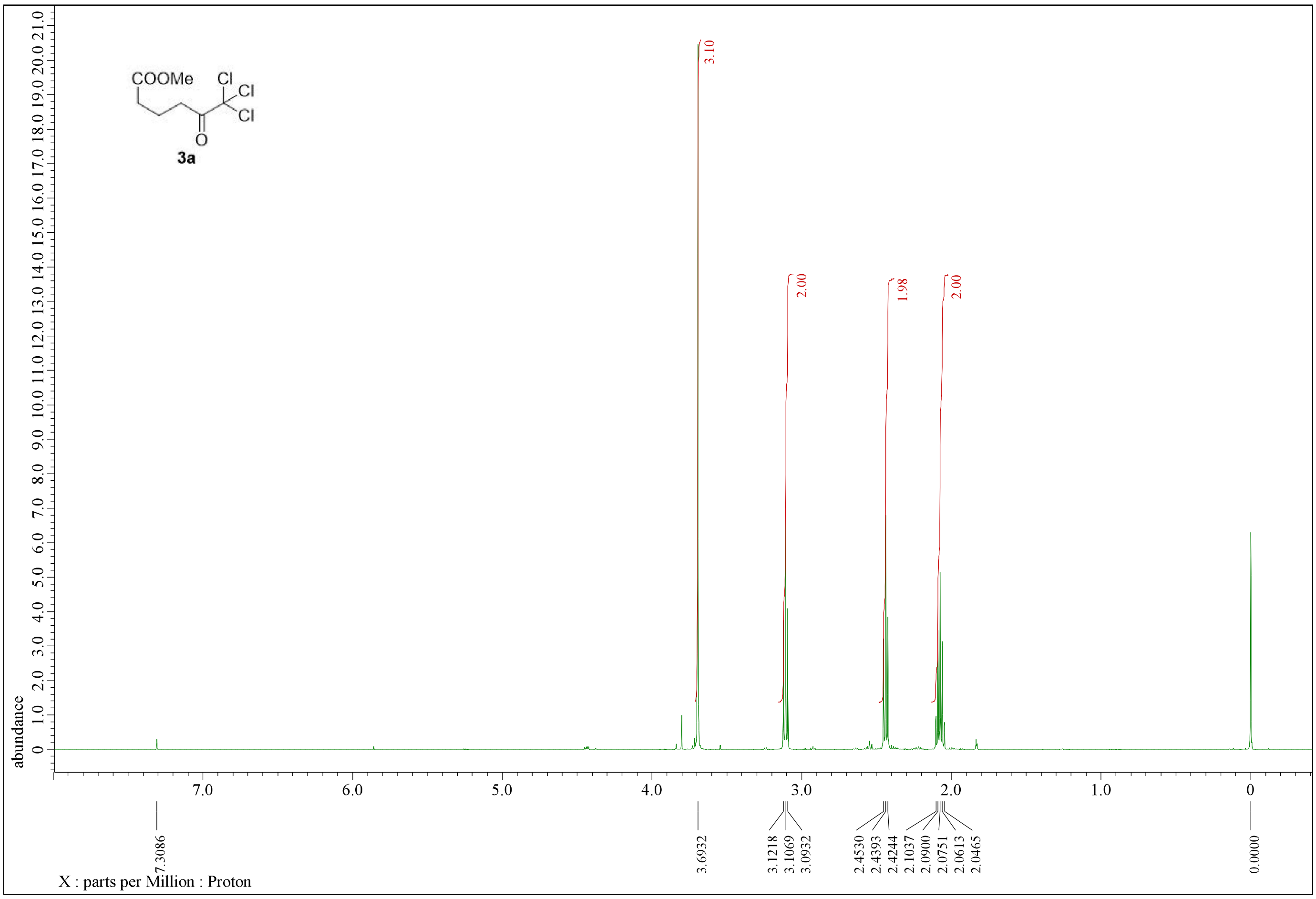
COOMe
Cl
Cl
Cl
O
3a
3.10
2.00
1.98
2.00
abundance
7.0
6.0
5.0
4.0
3.0
2.0
1.0
0
7.3086
3.6932
3.1218
3.1069
3.0932
2.4530
2.4393
2.4244
2.1037
2.0900
2.0751
2.0613
2.0465
0.0000
X : parts per Million : Proton

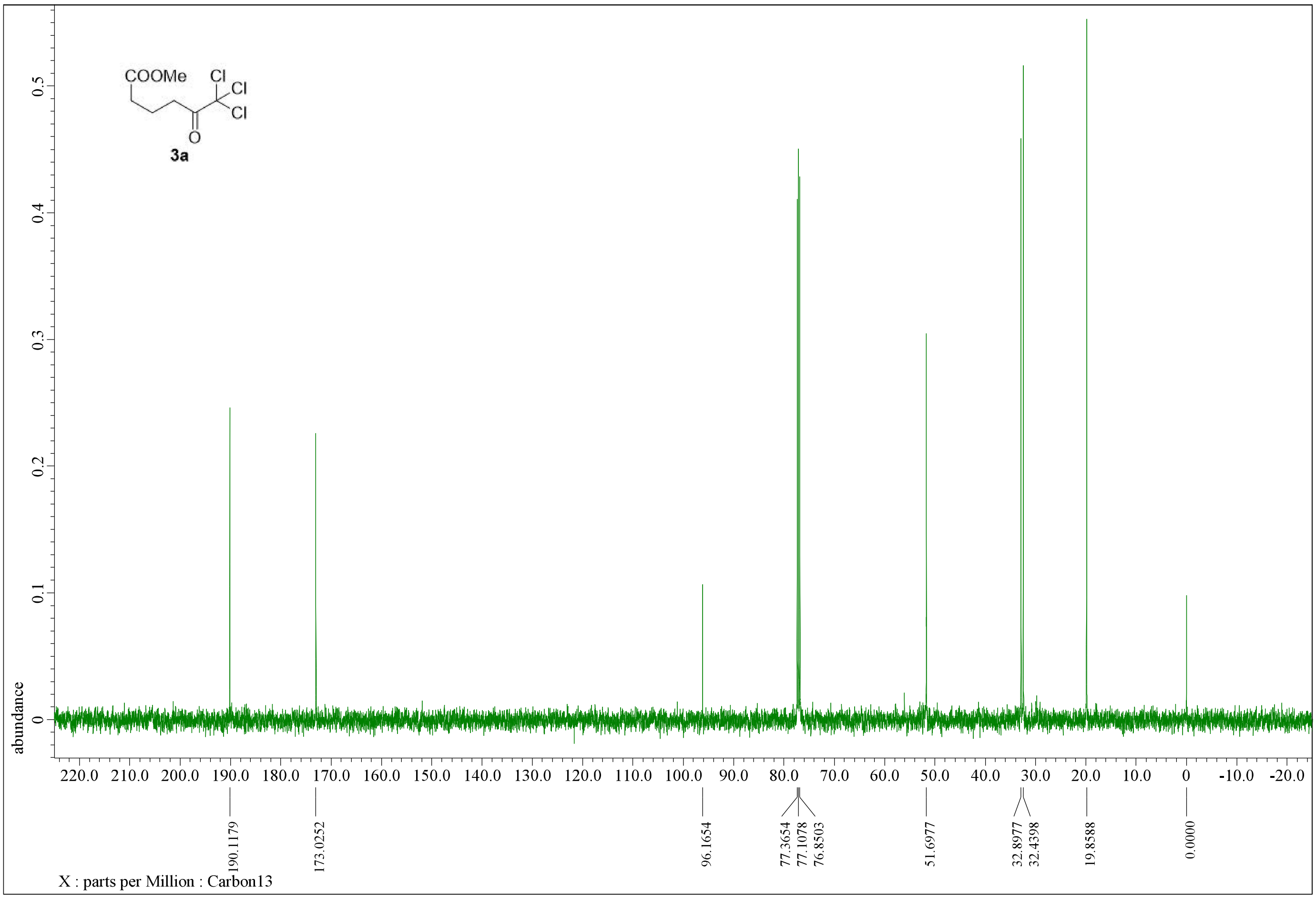
COOMe
Cl
Cl
Cl
O
3a
abundance
190.1179
173.0252
96.1654
77.3654
77.1078
76.8503
51.6977
32.8977
32.4398
19.8588
0.0000
X : parts per Million : Carbon13

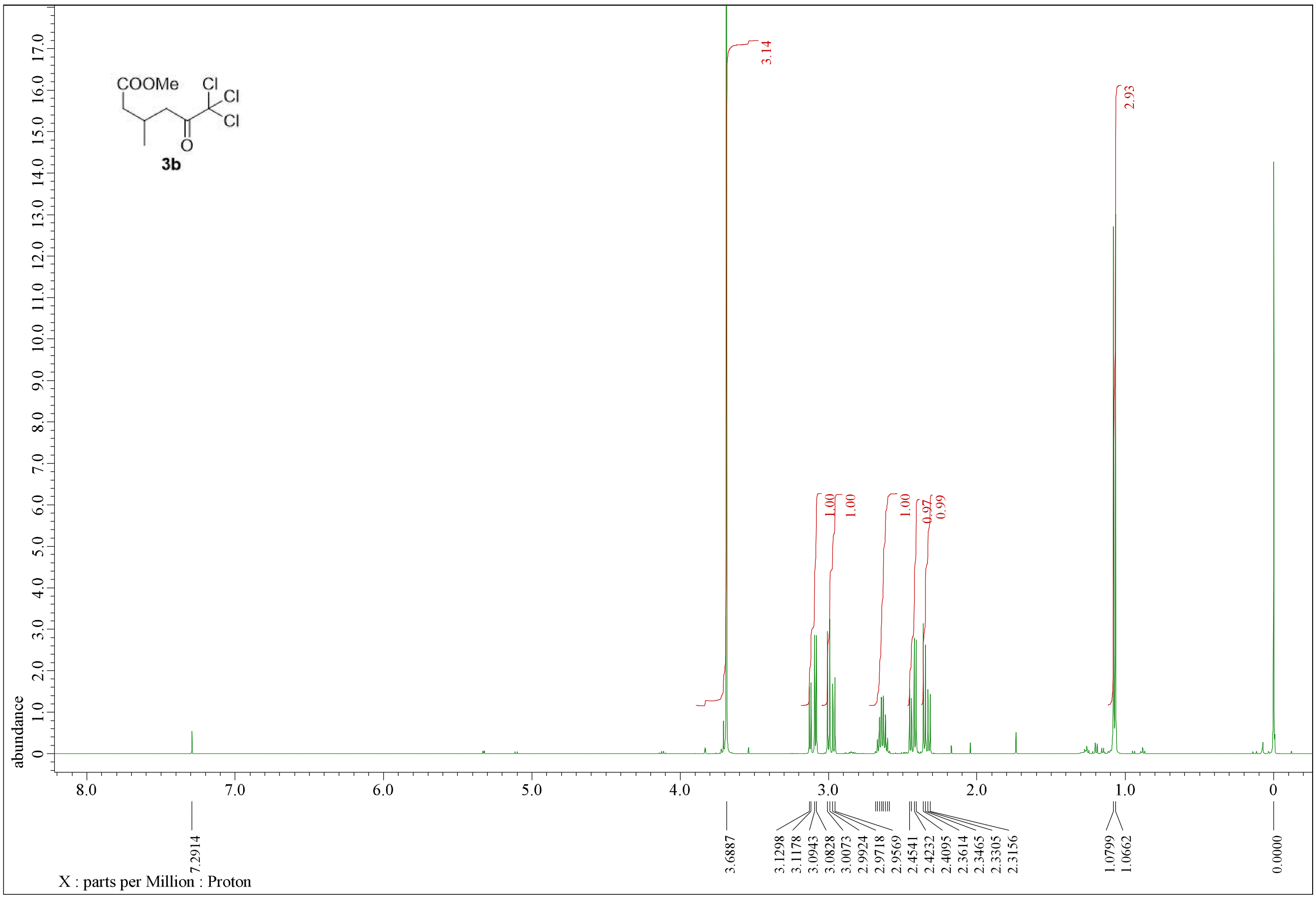
COOMe
Cl
Cl
Cl
O
3b
abundance
X : parts per Million : Proton
3.14
1.00
1.00
1.00
0.97
0.99
2.93
7.2914
3.6887
3.1298
3.1178
3.0943
3.0828
3.0073
2.9924
2.9718
2.9569
2.4541
2.4232
2.4095
2.3614
2.3465
2.3305
2.3156
1.0799
1.0662
0.0000

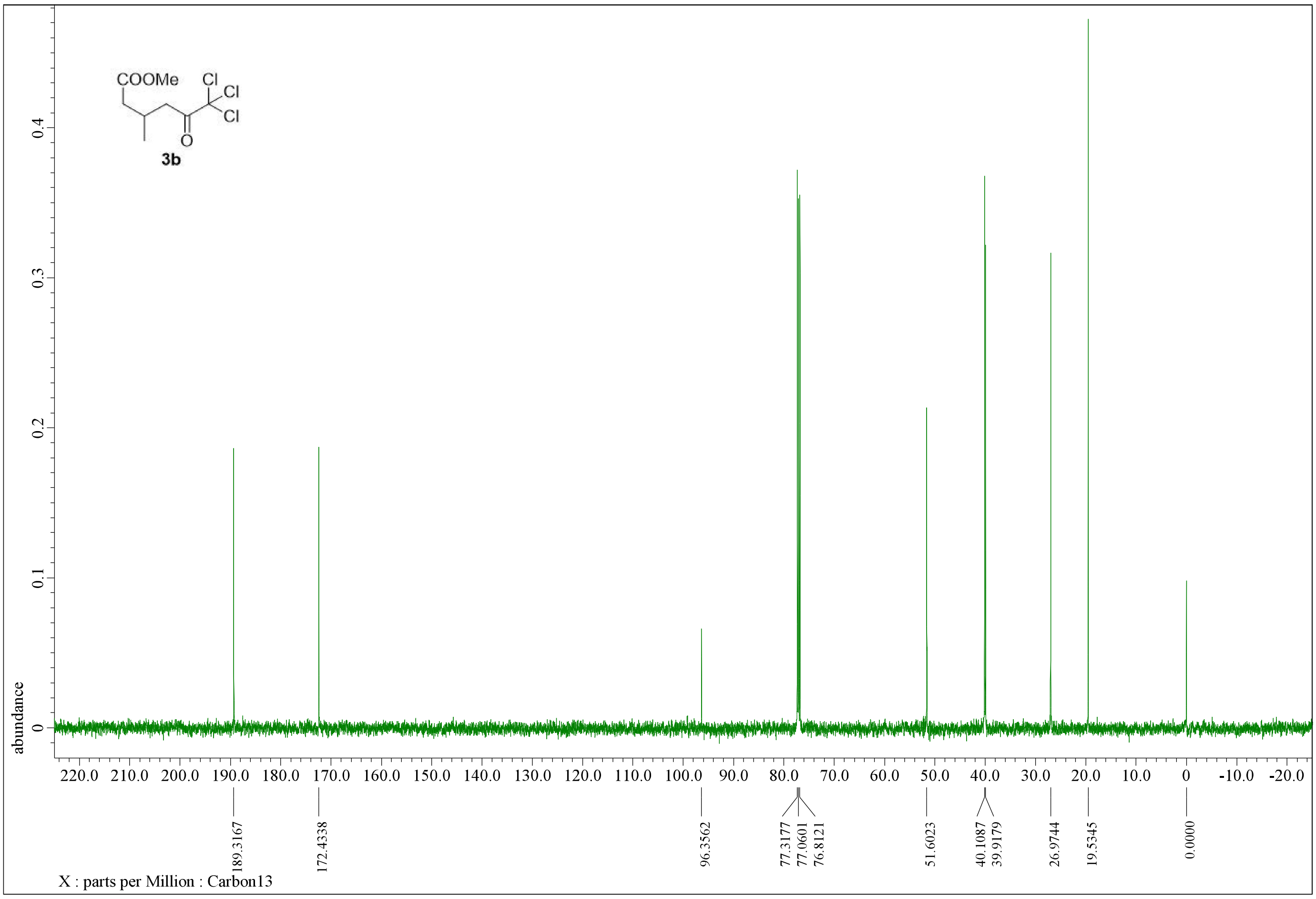
COOMe
Cl
Cl
Cl
O
3b
abundance
X : parts per Million : Carbon13
189.3167
172.4338
96.3562
77.3177
77.0601
76.8121
51.6023
40.1087
39.9179
26.9744
19.5345
0.0000

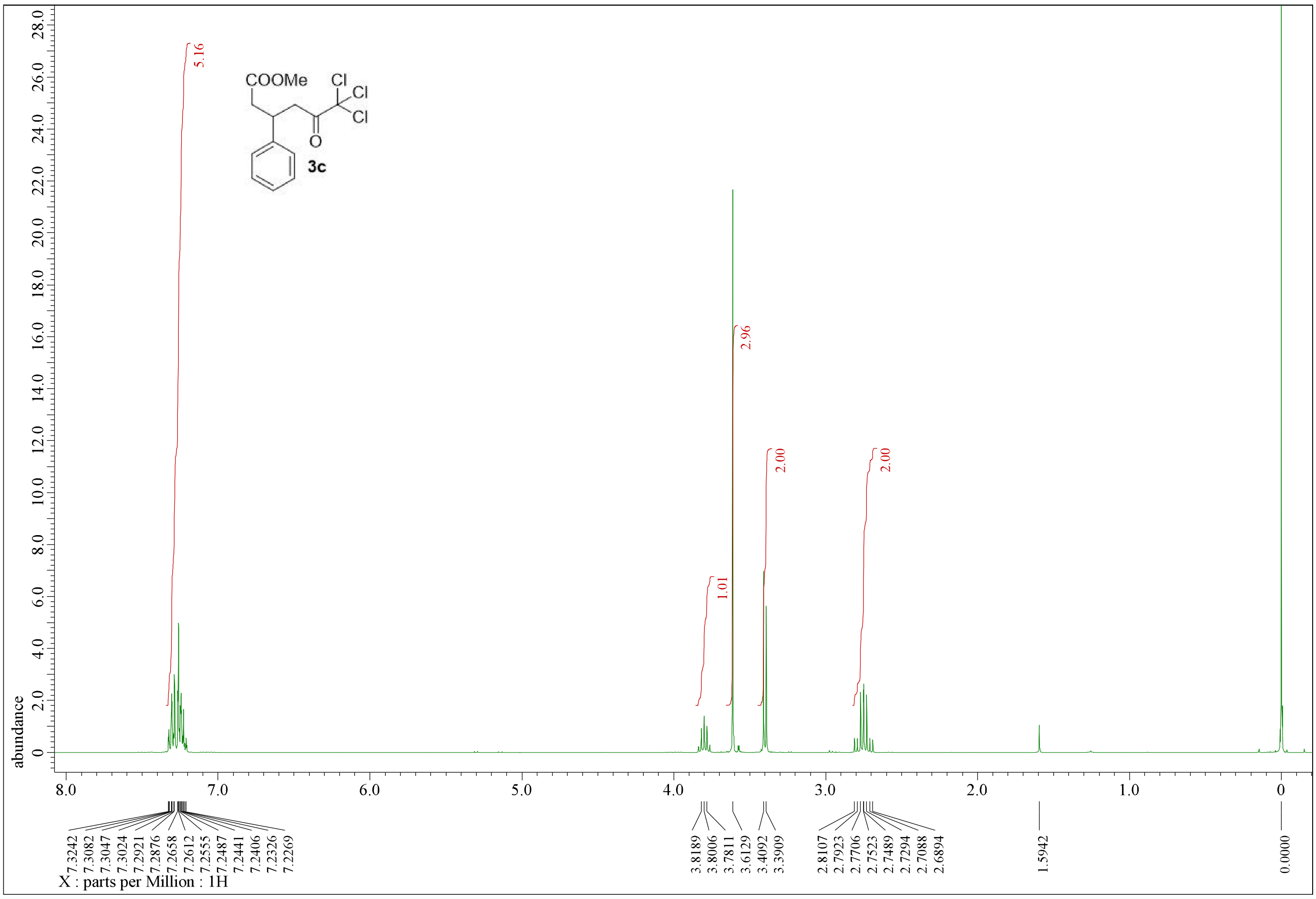

COOMe
Cl
Cl
Cl
O
3c
5.16
2.96
2.00
2.00
1.01
abundance
X : parts per Million : 1H
7.3242
7.3082
7.3047
7.3024
7.2921
7.2876
7.2658
7.2612
7.2555
7.2487
7.2441
7.2406
7.2326
7.2269
3.8189
3.8006
3.7811
3.6129
3.4092
3.3909
2.8107
2.7923
2.7706
2.7523
2.7489
2.7294
2.7088
2.6894
1.5942
0.0000

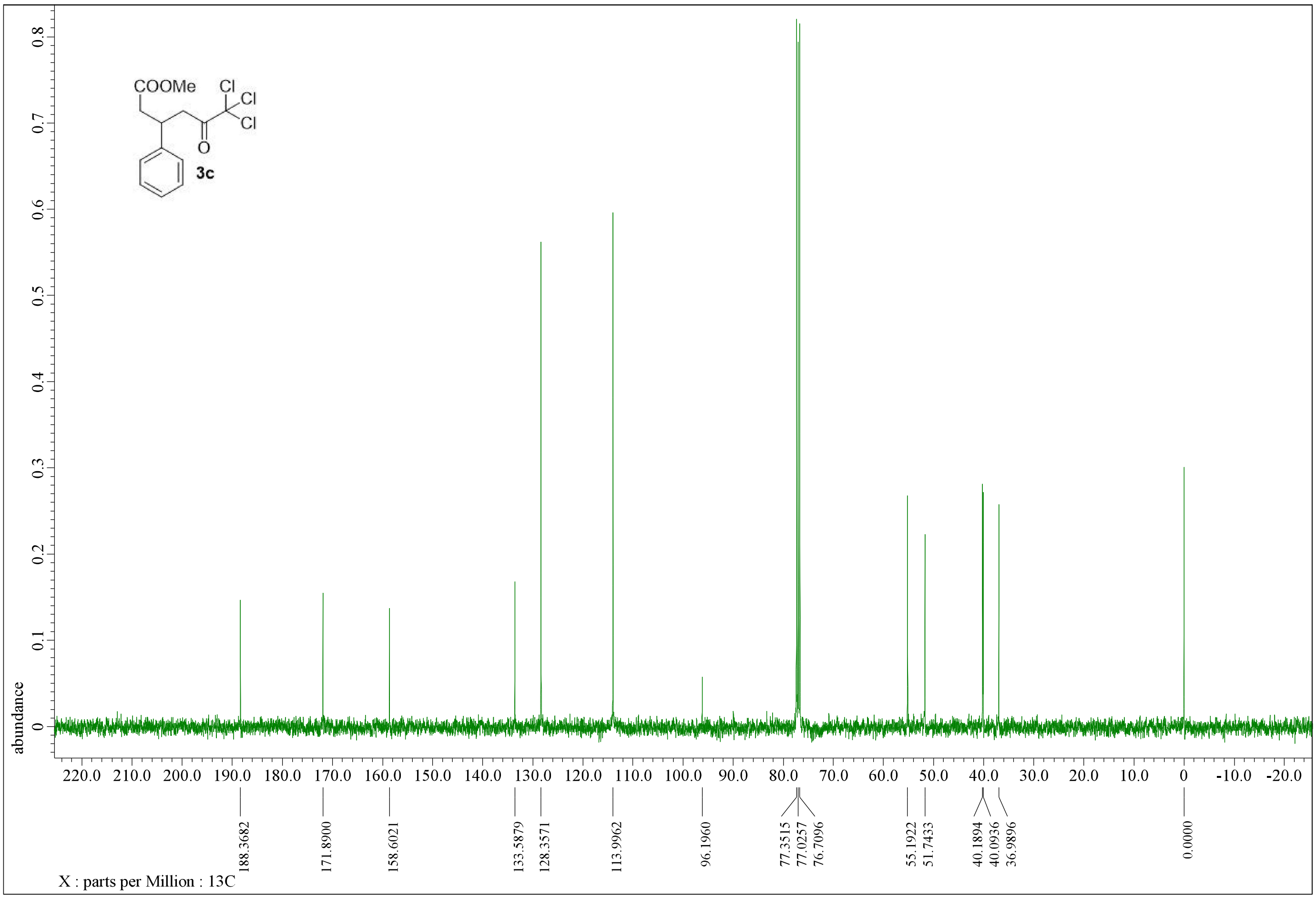

COOMe
Cl
Cl
Cl
O
3c
abundance
X : parts per Million : 13C
188.3682
171.8900
158.6021
133.5879
128.3571
113.9962
96.1960
77.3515
77.0257
76.7096
55.1922
51.7433
40.1894
40.0936
36.9896
0.0000

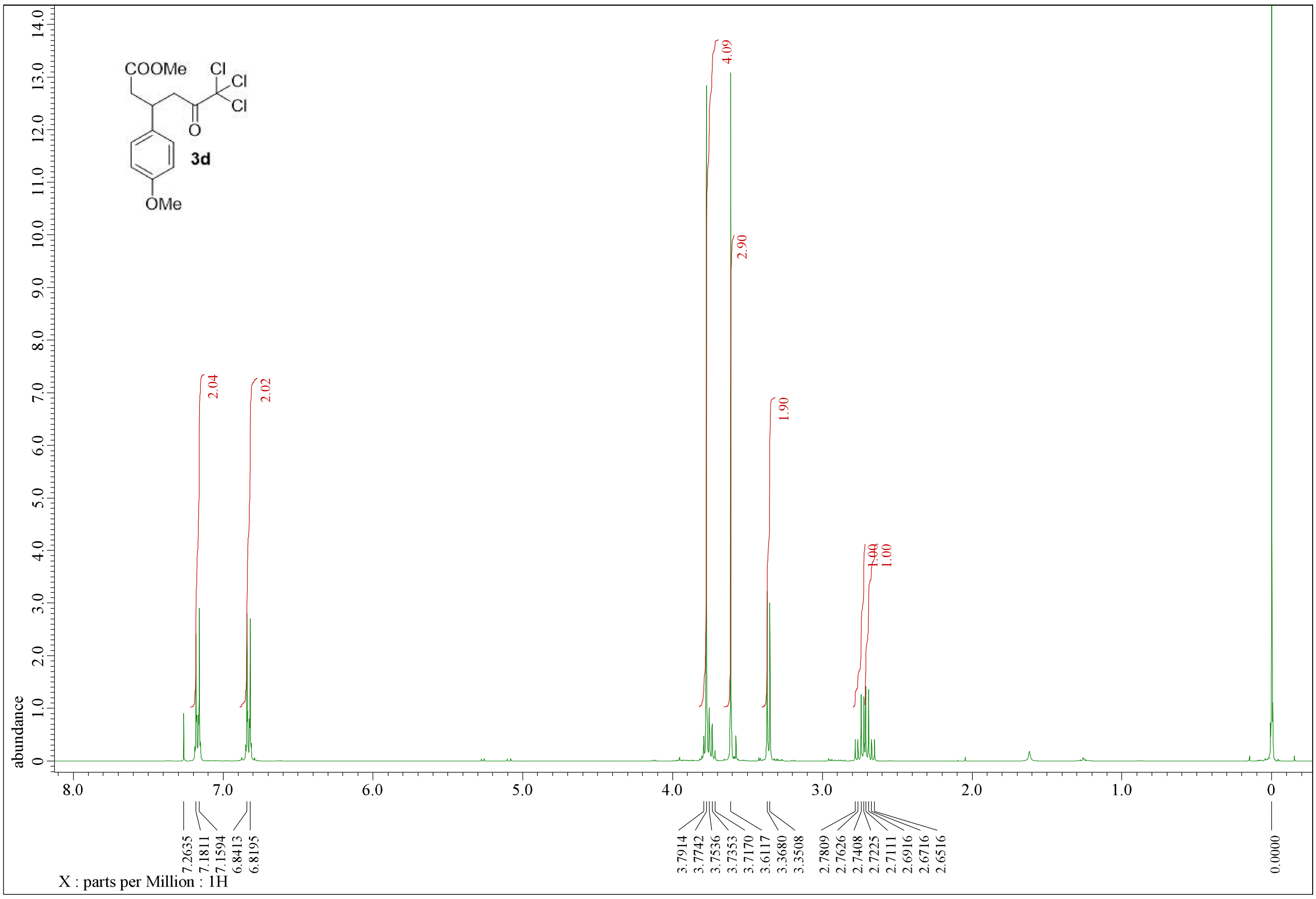
COOMe
Cl
Cl
Cl
O
3d
OMe
abundance
X : parts per Million : 1H
7.2635
7.1811
7.1594
6.8413
6.8195
3.7914
3.7742
3.7536
3.7353
3.7170
3.6117
3.3680
3.3508
2.7809
2.7626
2.7408
2.7225
2.7111
2.6916
2.6716
2.6516
0.0000
2.04
2.02
4.09
2.90
1.90
1.00
1.00

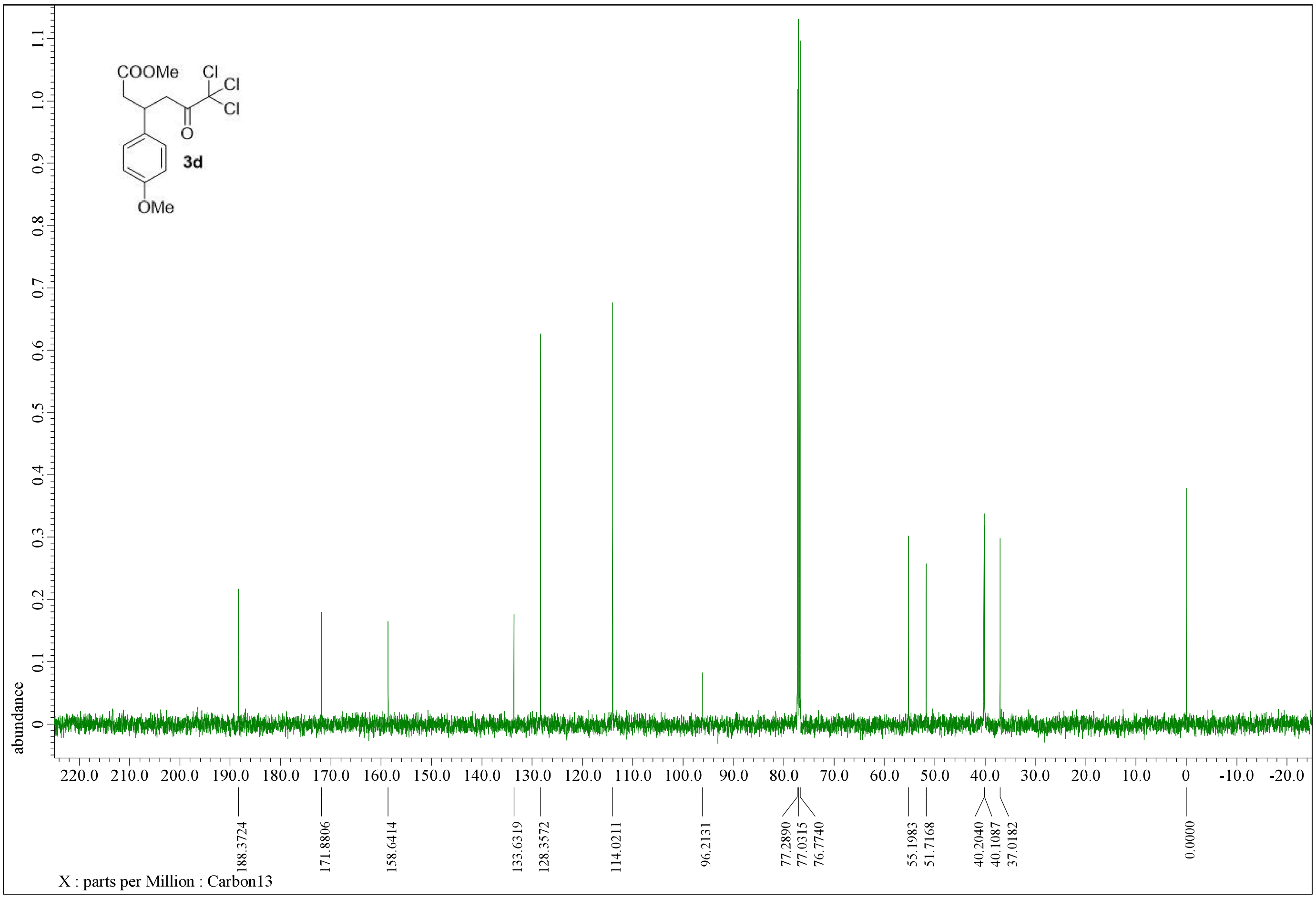
COOMe
Cl
Cl
Cl
O
3d
OMe
abundance
X : parts per Million : Carbon13
188.3724
171.8806
158.6414
133.6319
128.3572
114.0211
96.2131
77.2890
77.0315
76.7740
55.1983
51.7168
40.2040
40.1087
37.0182
0.0000

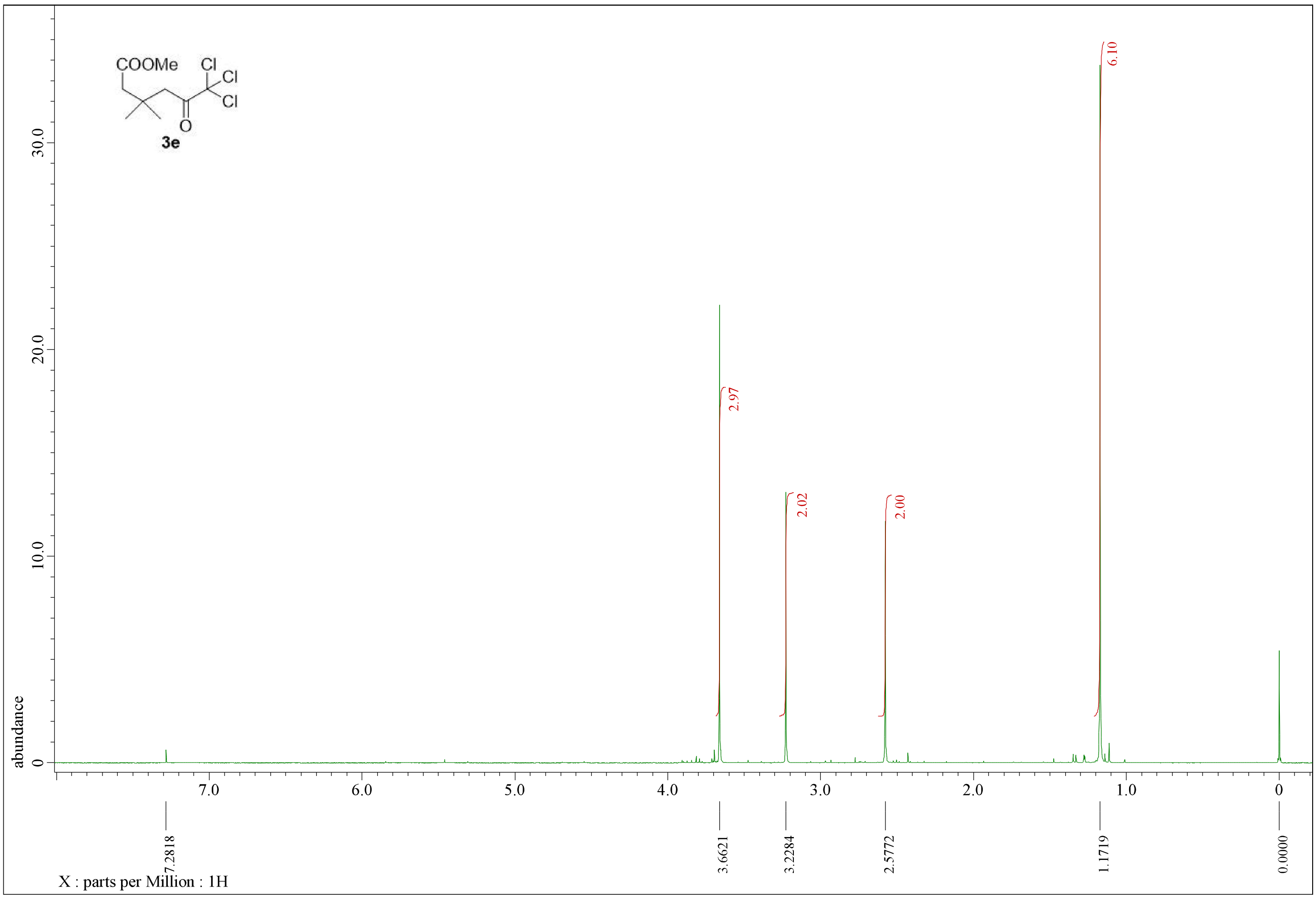
COOMe
Cl
Cl
Cl
O
3e
abundance
30.0
20.0
10.0
0
2.97
2.02
2.00
6.10
7.0
6.0
5.0
4.0
3.0
2.0
1.0
0
7.2818
3.6621
3.2284
2.5772
1.1719
0.0000
X : parts per Million : 1H

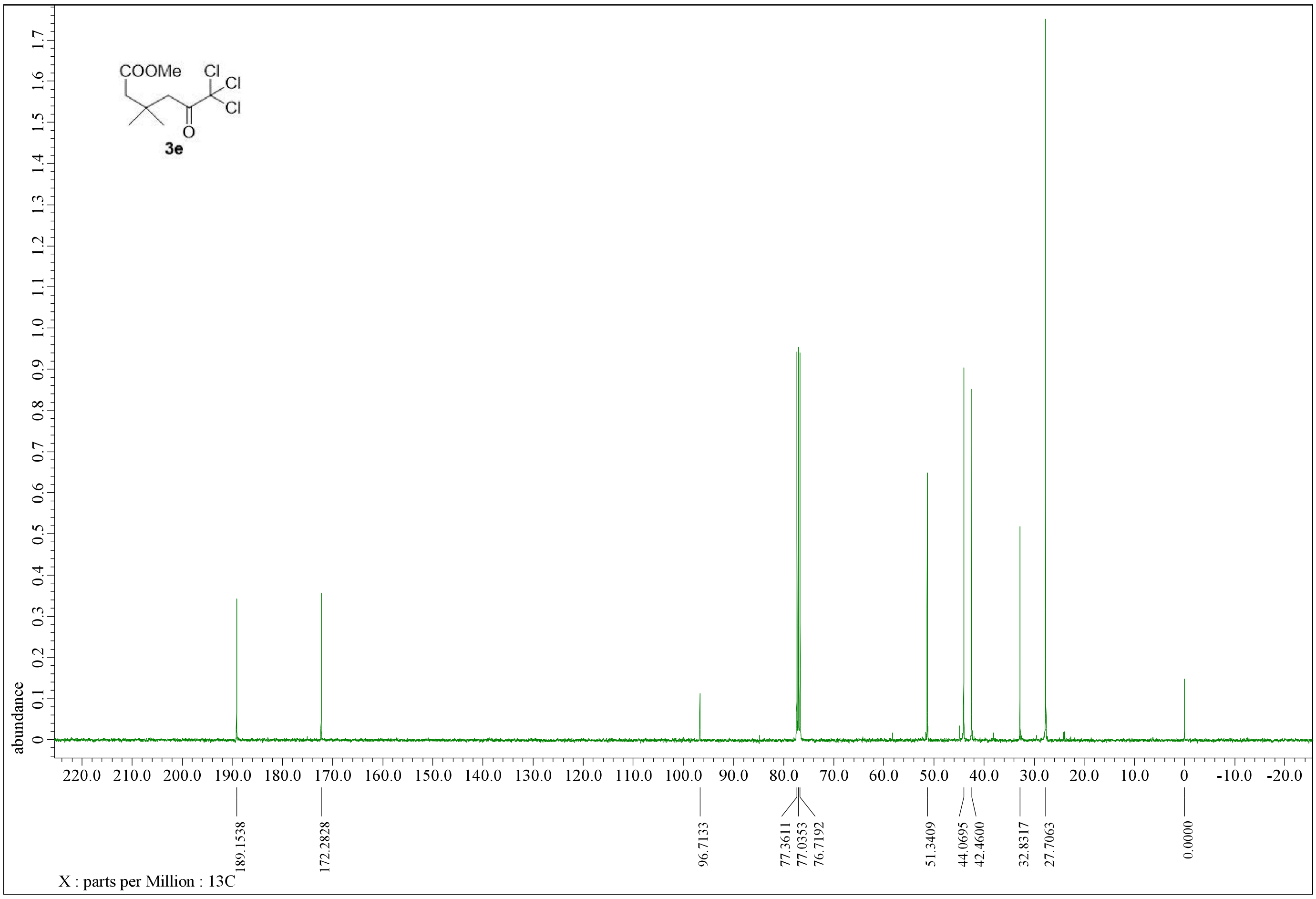
COOMe
Cl
Cl
Cl
O
3e
abundance
1.7
1.6
1.5
1.4
1.3
1.2
1.1
1.0
0.9
0.8
0.7
0.6
0.5
0.4
0.3
0.2
0.1
0
220.0 210.0 200.0 190.0 180.0 170.0 160.0 150.0 140.0 130.0 120.0 110.0 100.0 90.0 80.0 70.0 60.0 50.0 40.0 30.0 20.0 10.0 0 -10.0 -20.0
189.1538
172.2828
96.7133
77.3611
77.0353
76.7192
51.3409
44.0695
42.4600
32.8317
27.7063
0.0000
X : parts per Million : 13C

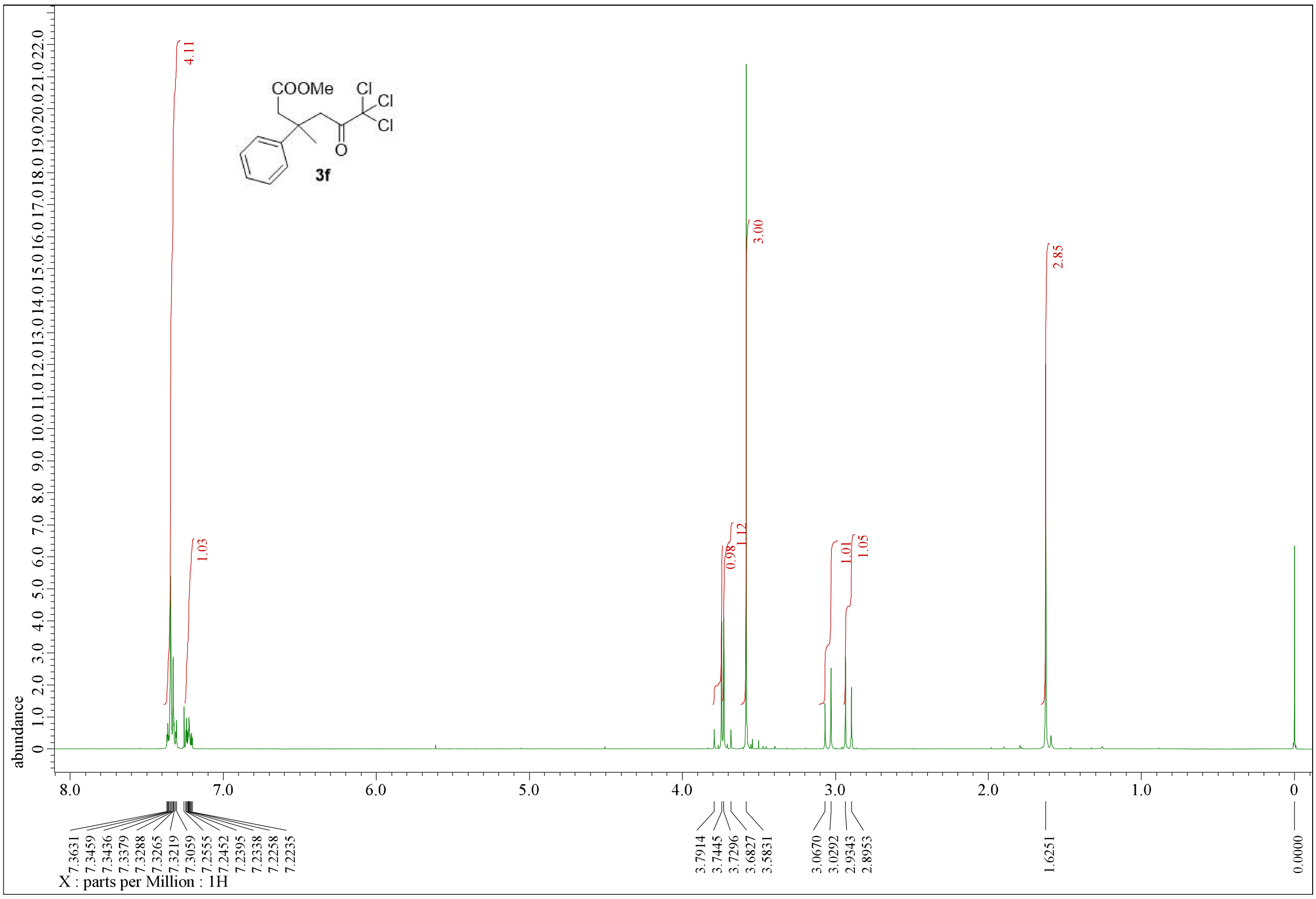
COOMe
Cl
Cl
Cl
O
3f
4.11
1.03
0.98
1.12
3.00
1.01
1.05
2.85
abundance
7.3631
7.3459
7.3436
7.3379
7.3288
7.3265
7.3219
7.3059
7.2555
7.2452
7.2395
7.2338
7.2258
7.2235
3.7914
3.7445
3.7296
3.6827
3.5831
3.0670
3.0292
2.9343
2.8953
1.6251
0.0000
X : parts per Million : 1H

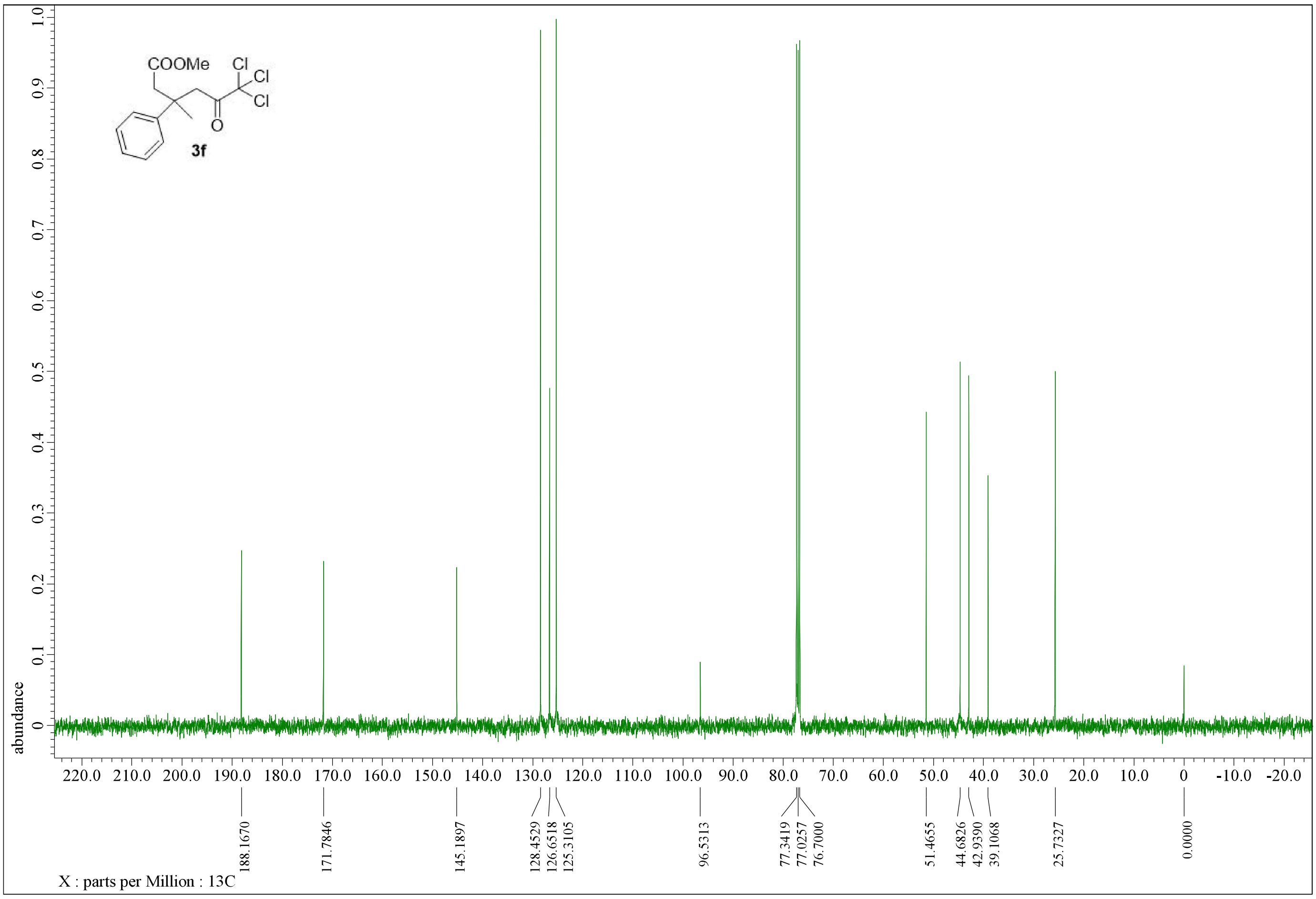
COOMe
Cl
Cl
Cl
O
3f
abundance
188.1670
171.7846
145.1897
128.4529
126.6518
125.3105
96.5313
77.3419
77.0257
76.7000
51.4655
44.6826
42.9390
39.1068
25.7327
0.0000
X : parts per Million : 13C

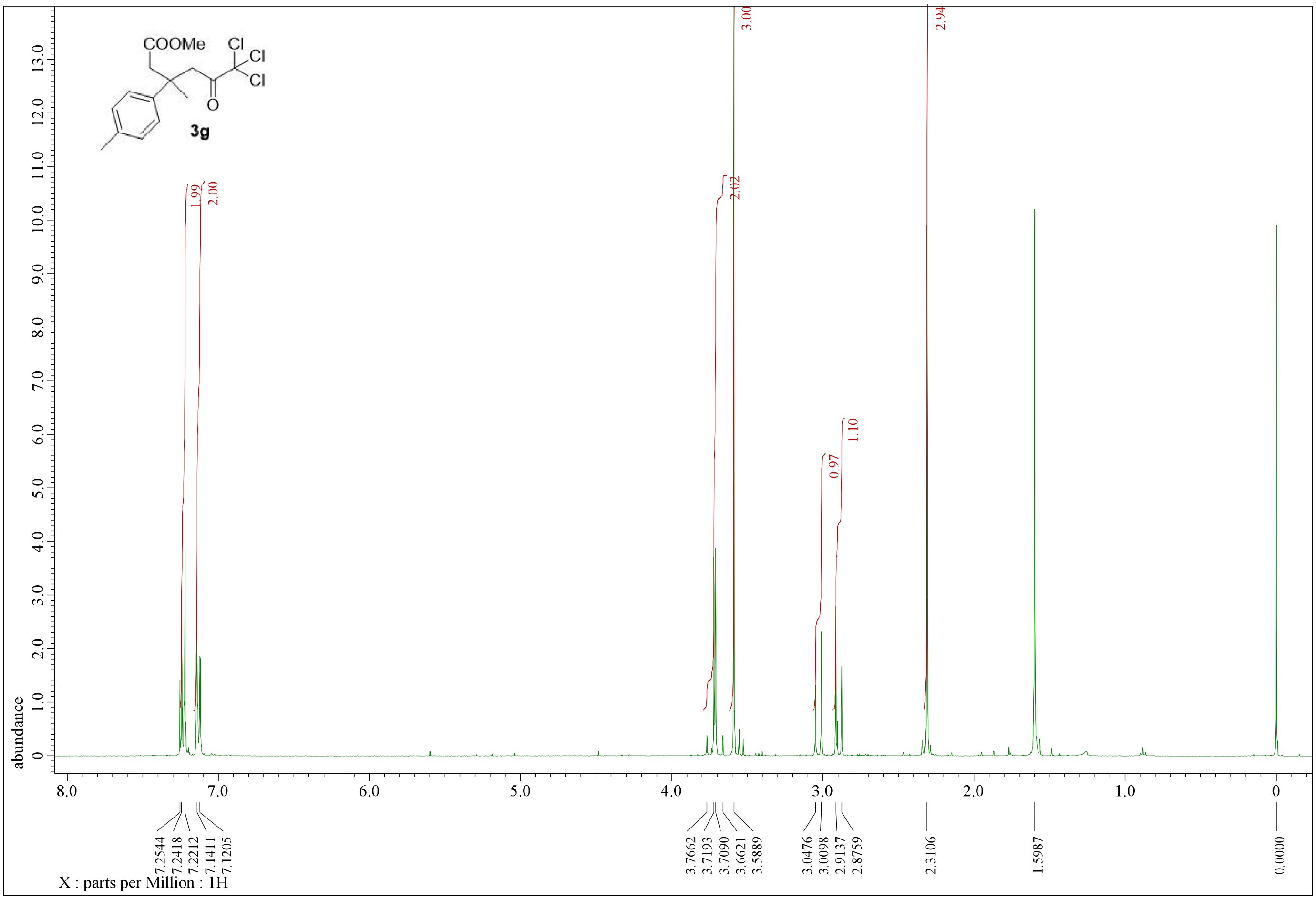
COOMe
Cl
Cl
Cl
O
3g
abundance
X : parts per Million : 1H
1.99
2.00
2.02
3.00
0.97
1.10
2.94
7.2544
7.2418
7.2212
7.1411
7.1205
3.7662
3.7193
3.7090
3.6621
3.5889
3.0476
3.0098
2.9137
2.8759
2.3106
1.5987
0.0000

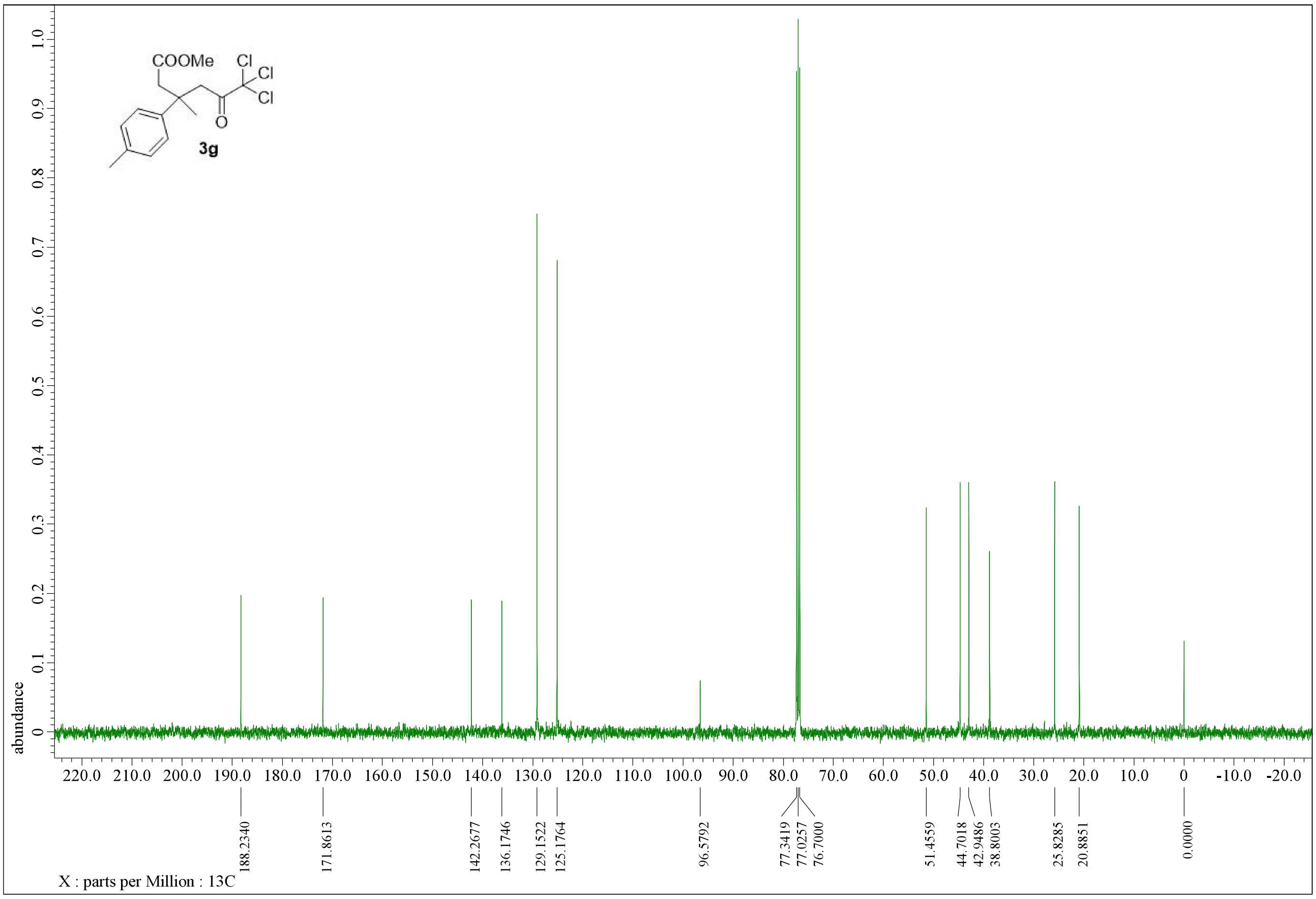
COOMe
Cl
Cl
Cl
O
3g
abundance
X : parts per Million : 13C
188.2340
171.8613
142.2677
136.1746
129.1522
125.1764
96.5792
77.3419
77.0257
76.7000
51.4559
44.7018
42.9486
38.8003
25.8285
20.8851
0.0000

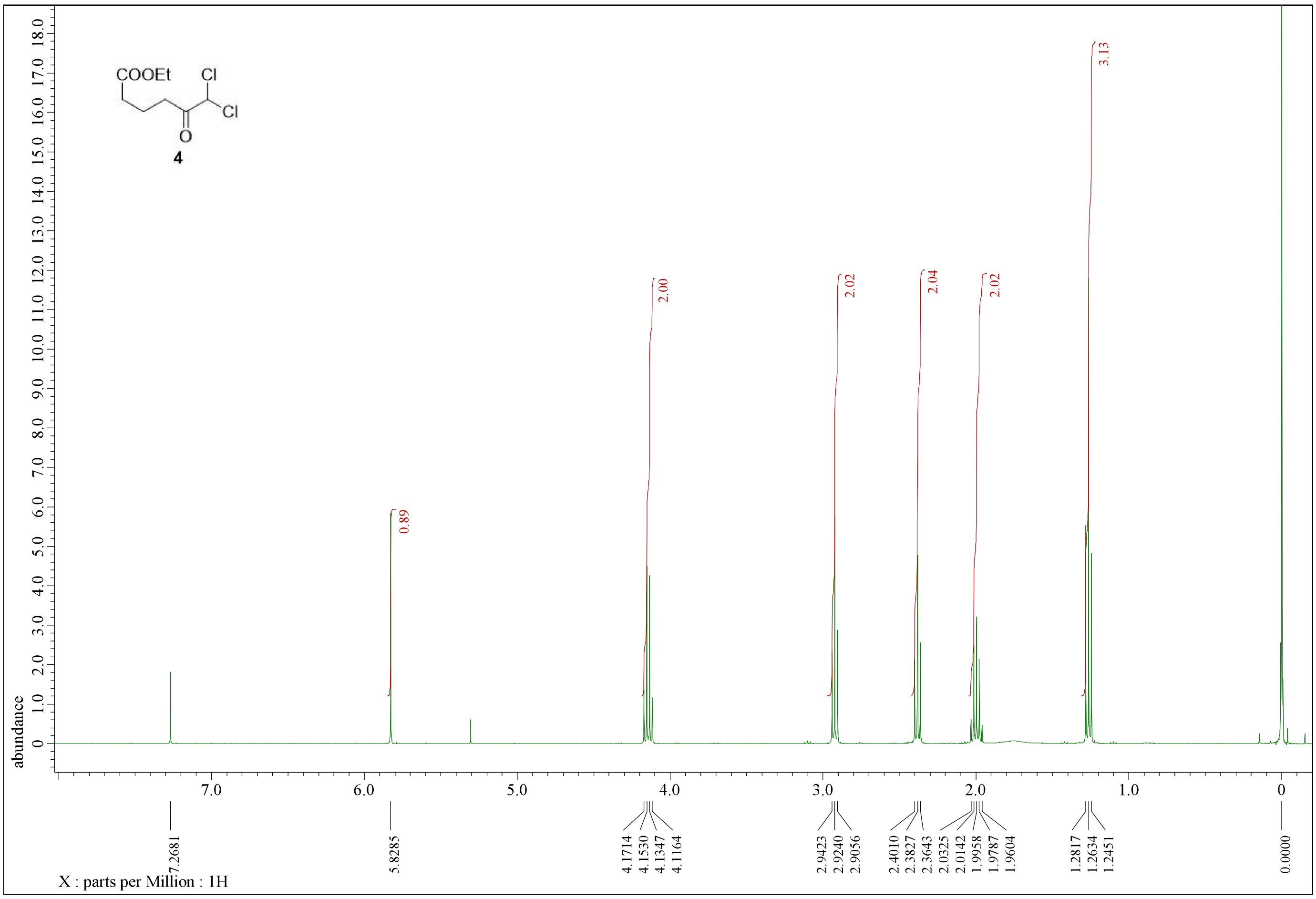
COOEt
Cl
Cl
O
4
abundance
0.89
2.00
2.02
2.04
2.02
3.13
7.2681
5.8285
4.1714
4.1530
4.1347
4.1164
2.9423
2.9240
2.9056
2.4010
2.3827
2.3643
2.0325
2.0142
1.9958
1.9787
1.9604
1.2817
1.2634
1.2451
0.0000
X : parts per Million : 1H

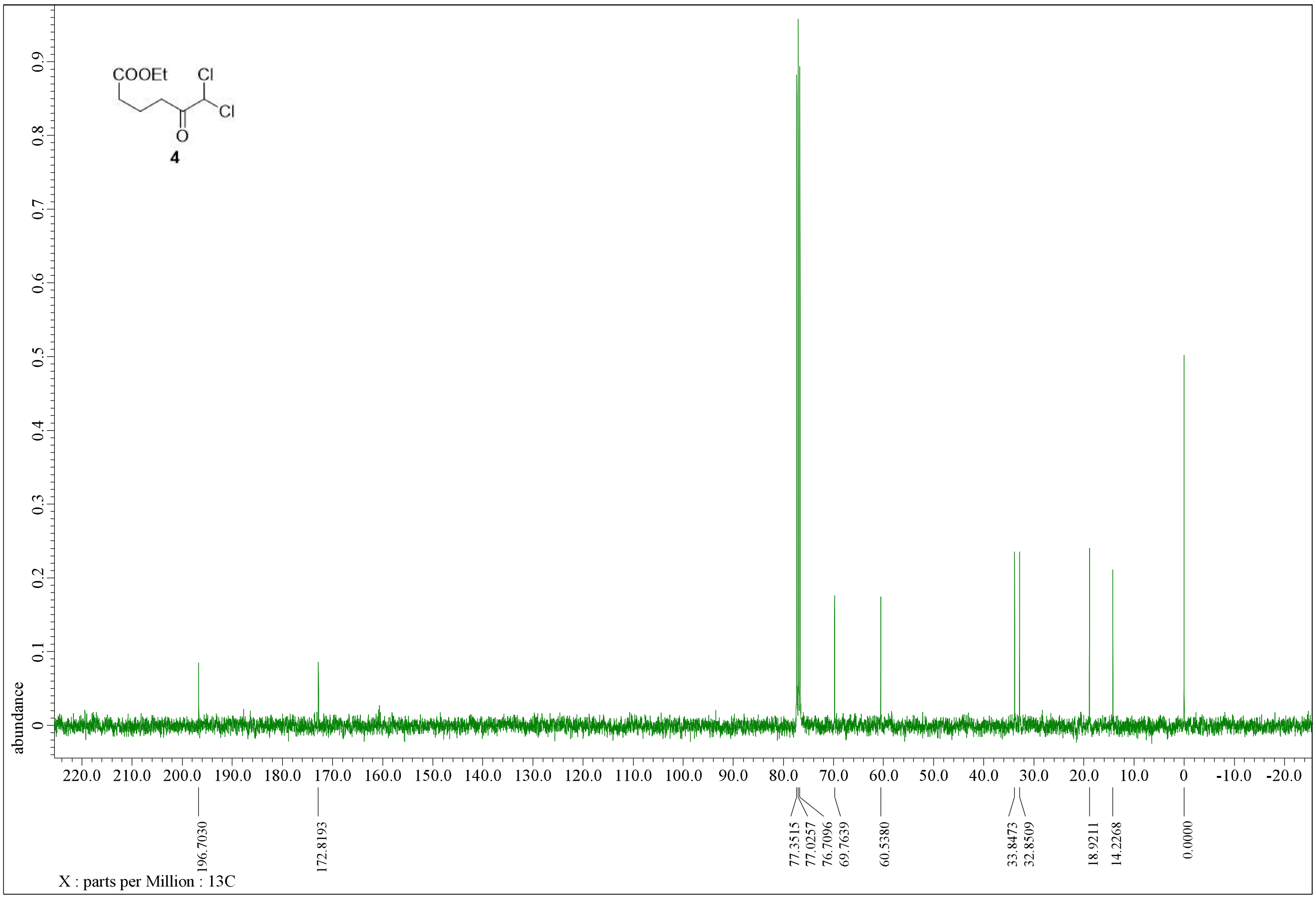
COOEt
Cl
Cl
O
4
abundance
196.7030
172.8193
77.3515
77.0257
76.7096
69.7639
60.5380
33.8473
32.8509
18.9211
14.2268
0.0000
X : parts per Million : 13C

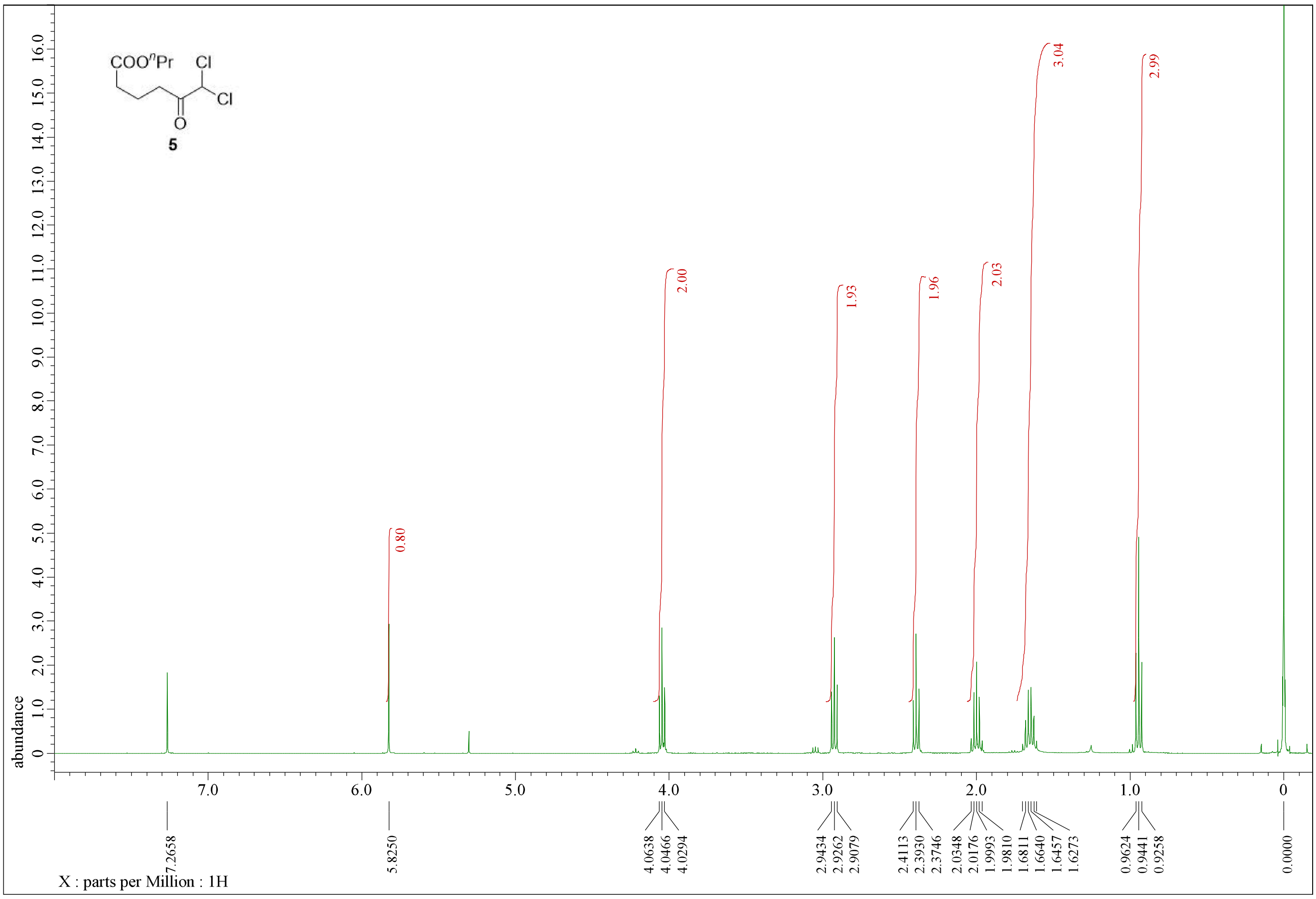
COO^nPr Cl
Cl
O
5
abundance
X : parts per Million : 1H
7.2658
5.8250
4.0638 4.0466 4.0294
2.9434 2.9262 2.9079
2.4113 2.3930 2.3746
2.0348 2.0176 1.9993 1.9810
1.6811 1.6640 1.6457 1.6273
0.9624 0.9441 0.9258
0.0000
0.80
2.00
1.93
1.96
2.03
3.04
2.99

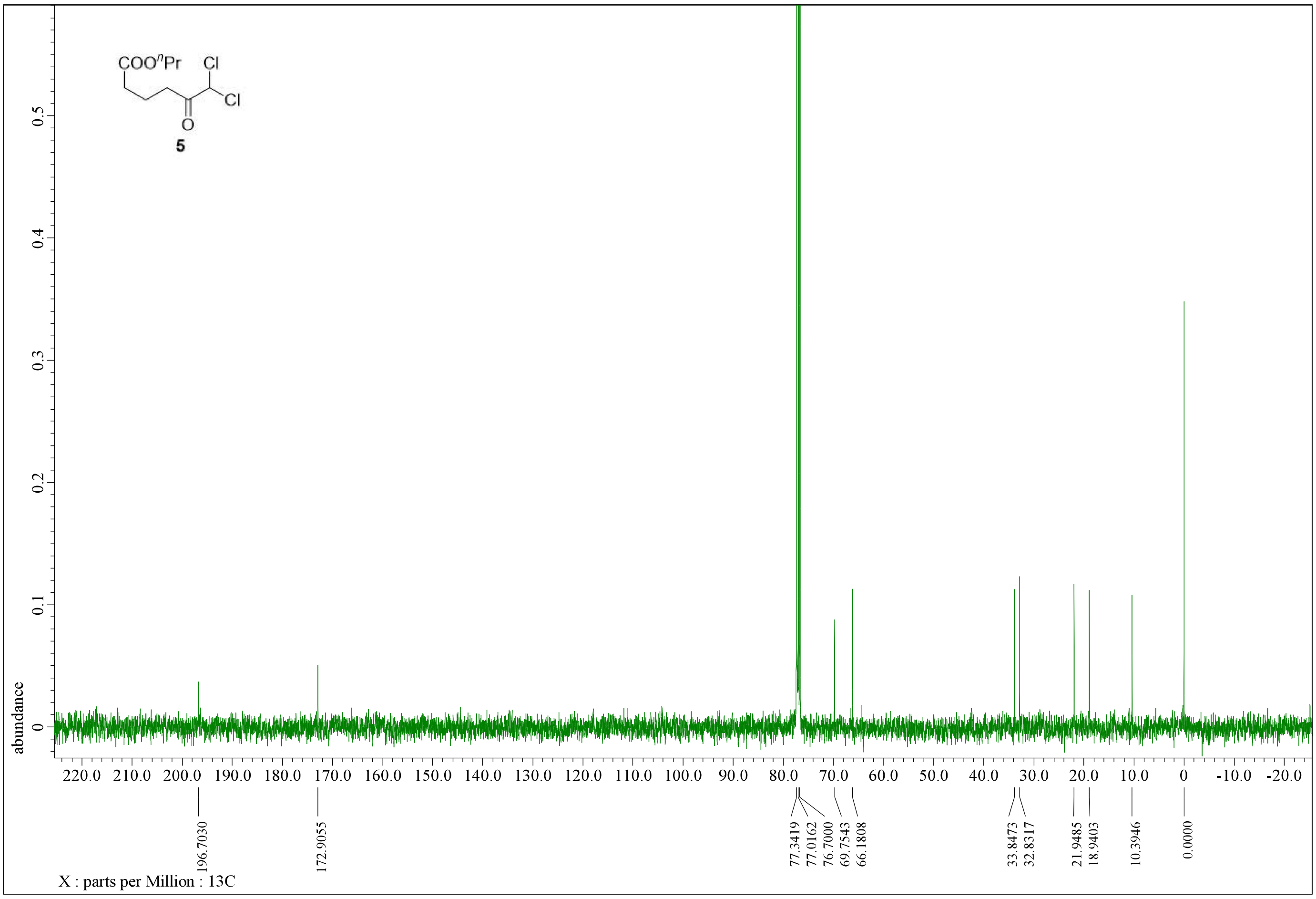
COO^nPr Cl
Cl
O
5
abundance
X : parts per Million : 13C
196.7030
172.9055
77.3419 77.0162 76.7000 69.7543 66.1808
33.8473 32.8317
21.9485 18.9403
10.3946
0.0000

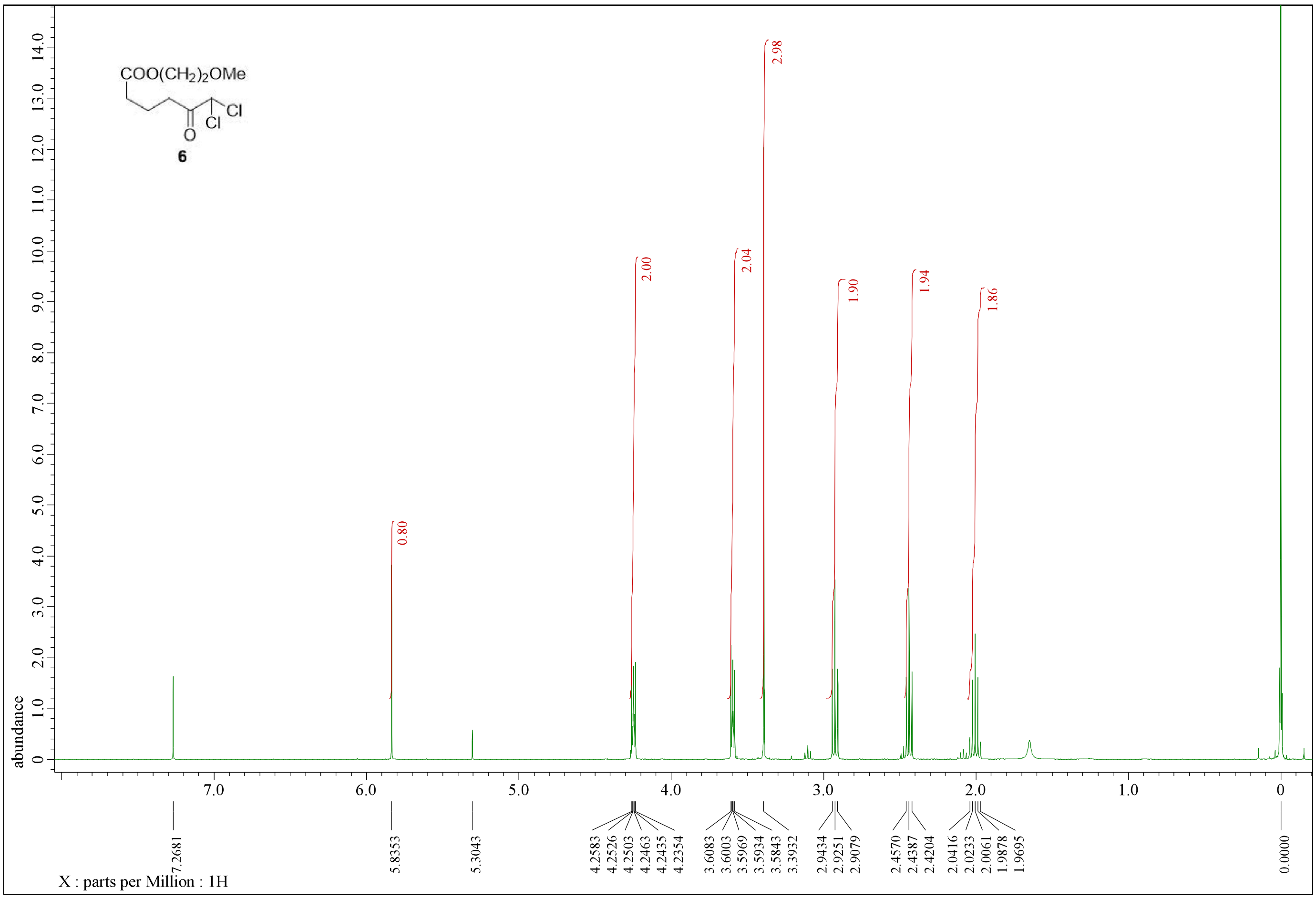
COO(CH2)2OMe
Cl
Cl
O
6
abundance
0.80
2.00
2.04
2.98
1.90
1.94
1.86
7.0
6.0
5.0
4.0
3.0
2.0
1.0
0
7.2681
5.8353
5.3043
4.2583
4.2526
4.2503
4.2463
4.2435
4.2354
3.6083
3.6003
3.5969
3.5934
3.5843
3.3932
2.9434
2.9251
2.9079
2.4570
2.4387
2.4204
2.0416
2.0233
2.0061
1.9878
1.9695
0.0000
X : parts per Million : 1H

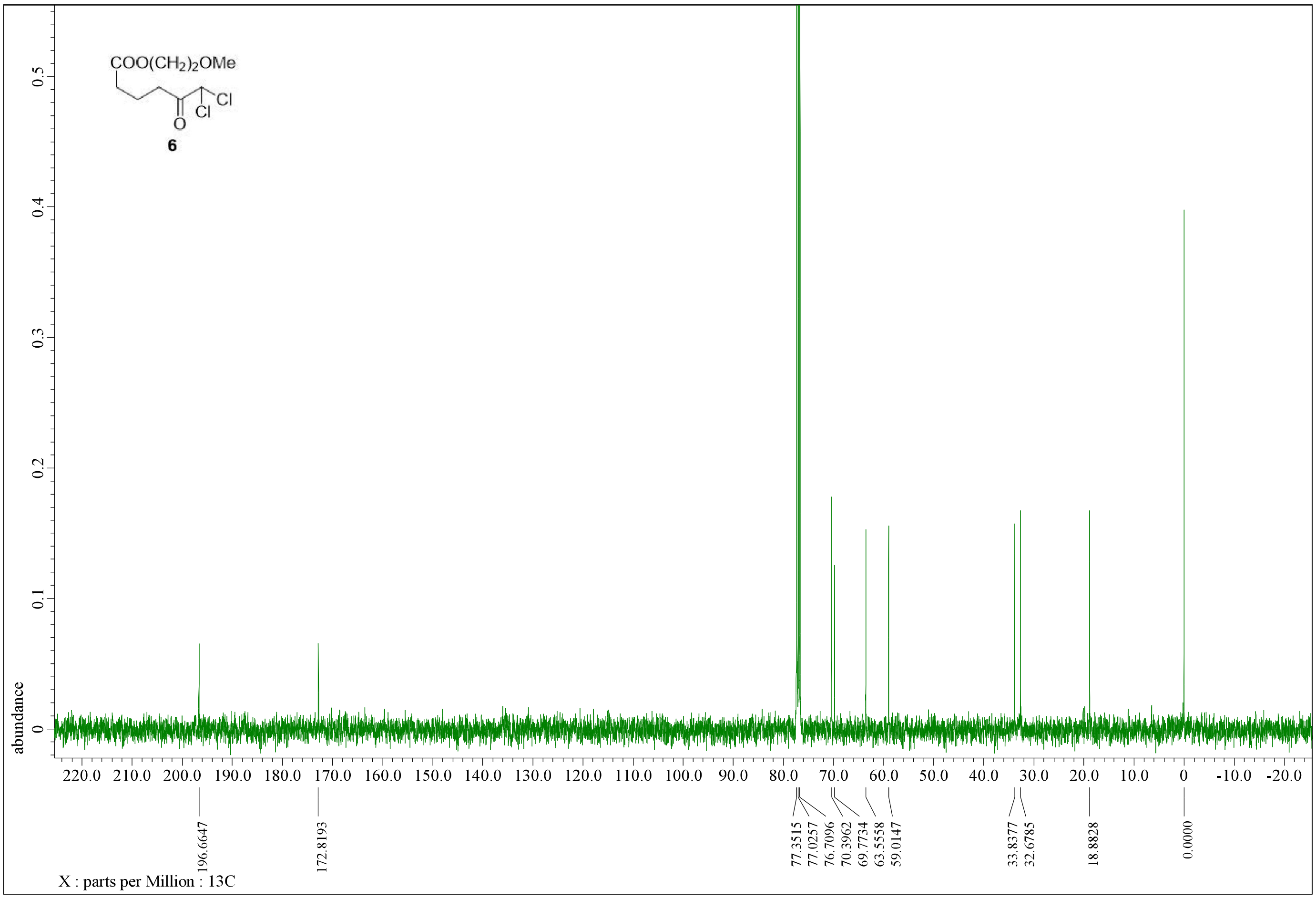
COO(CH2)2OMe
Cl
Cl
O
6
abundance
196.6647
172.8193
77.3515
77.0257
76.7096
70.3962
69.7734
63.5558
59.0147
33.8377
32.6785
18.8828
0.0000
X : parts per Million : 13C

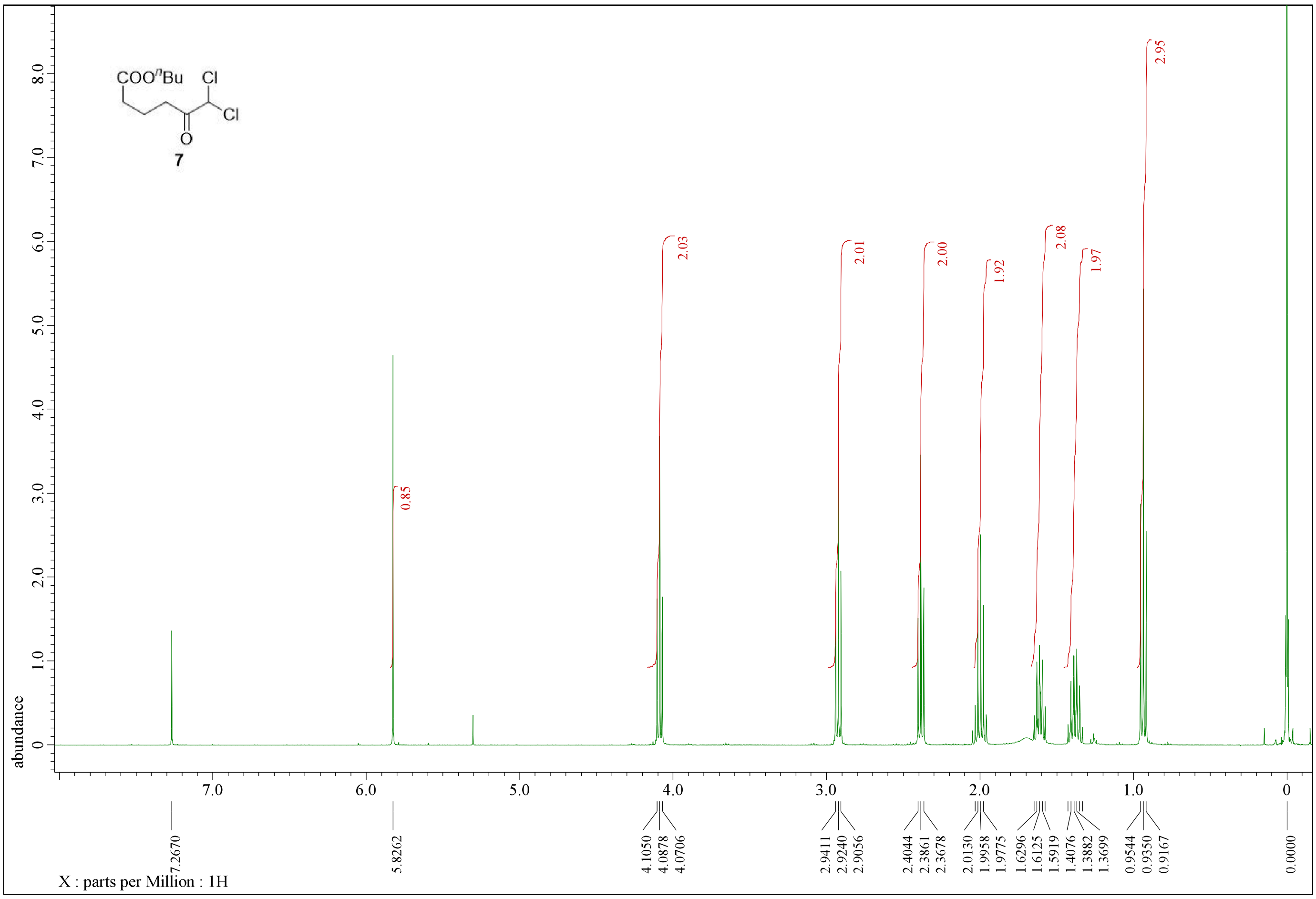
COO^{n}Bu Cl
Cl
O
7
abundance
X : parts per Million : 1H
7.2670
5.8262
4.1050 4.0878 4.0706
2.9411 2.9240 2.9056
2.4044 2.3861 2.3678
2.0130 1.9958 1.9775
1.6296 1.6125 1.5919
1.4076 1.3882 1.3699
0.9544 0.9350 0.9167
0.0000
0.85 2.03 2.01 2.00 1.92 2.08 1.97 2.95

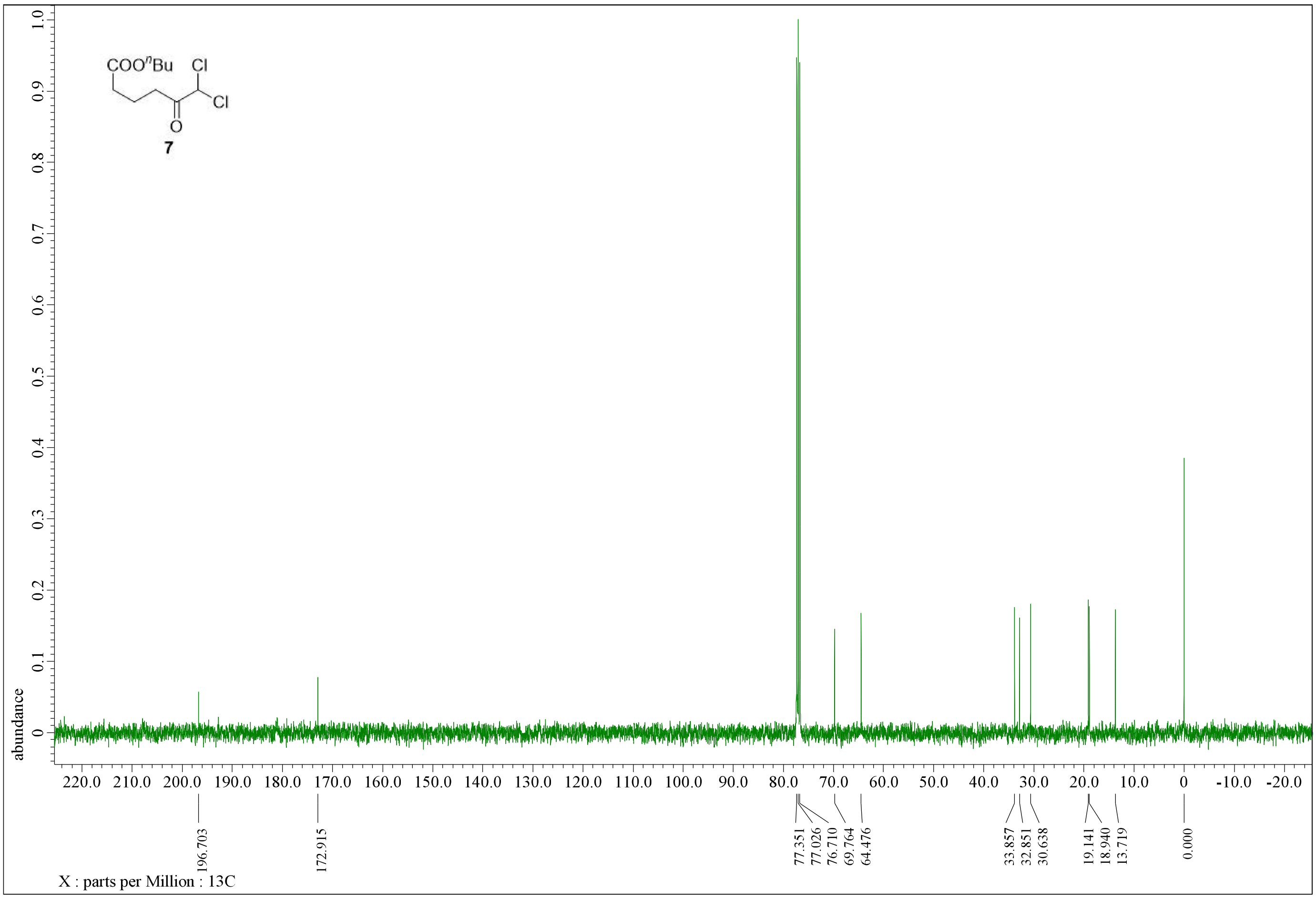
COO^{n}Bu Cl
Cl
O
7
abundance
X : parts per Million : 13C
196.703
172.915
77.351 77.026 76.710 69.764 64.476
33.857 32.851 30.638
19.141 18.940 13.719
0.000

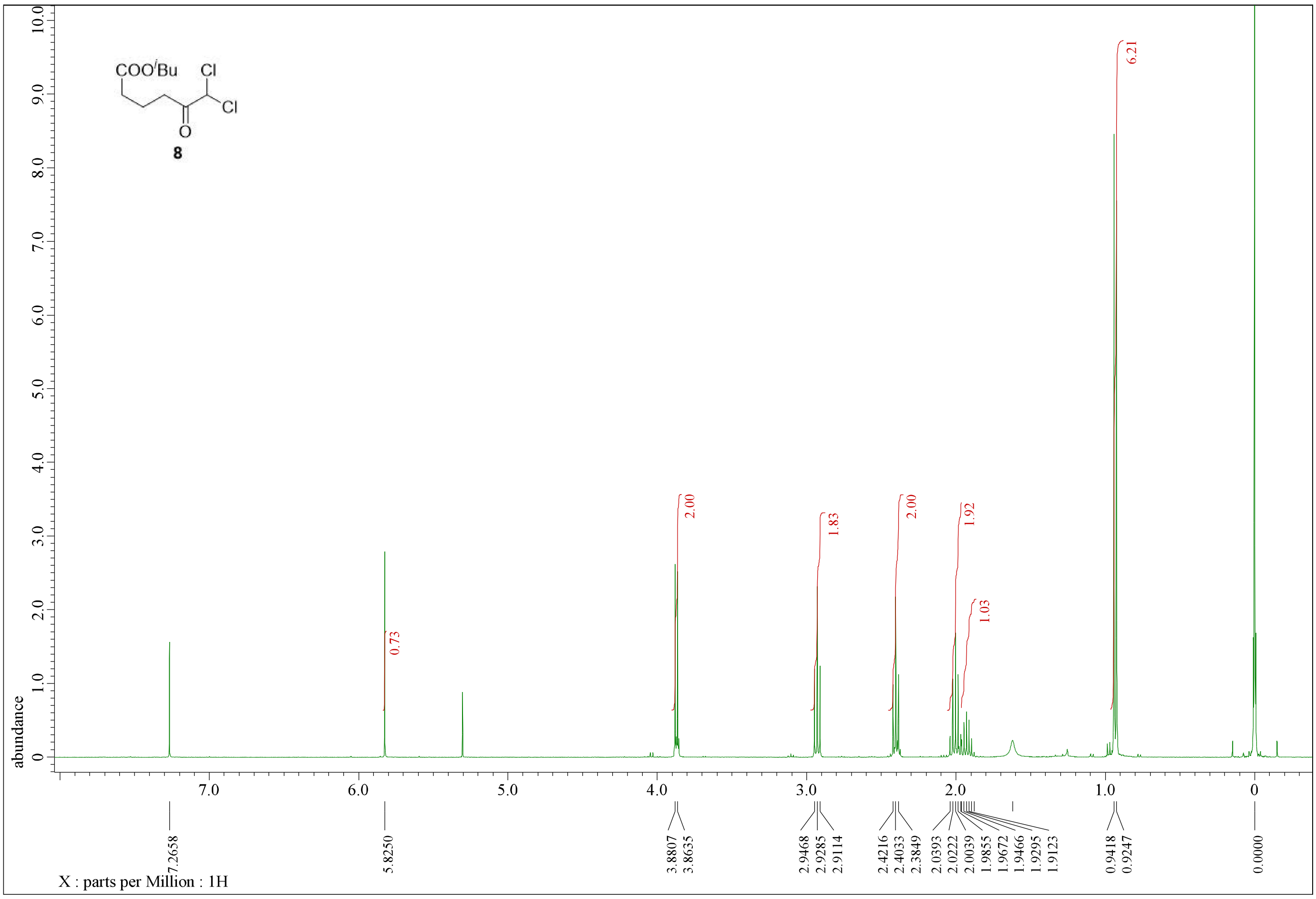
COO^iBu Cl
Cl
O
8
abundance
10.0 9.0 8.0 7.0 6.0 5.0 4.0 3.0 2.0 1.0 0
0.73
2.00
1.83
2.00
1.92
1.03
6.21
7.0 6.0 5.0 4.0 3.0 2.0 1.0 0
7.2658
5.8250
3.8807 3.8635
2.9468 2.9285 2.9114
2.4216 2.4033 2.3849
2.0393 2.0222 2.0039 1.9855 1.9672 1.9466 1.9295 1.9123
0.9418 0.9247
0.0000
X : parts per Million : 1H

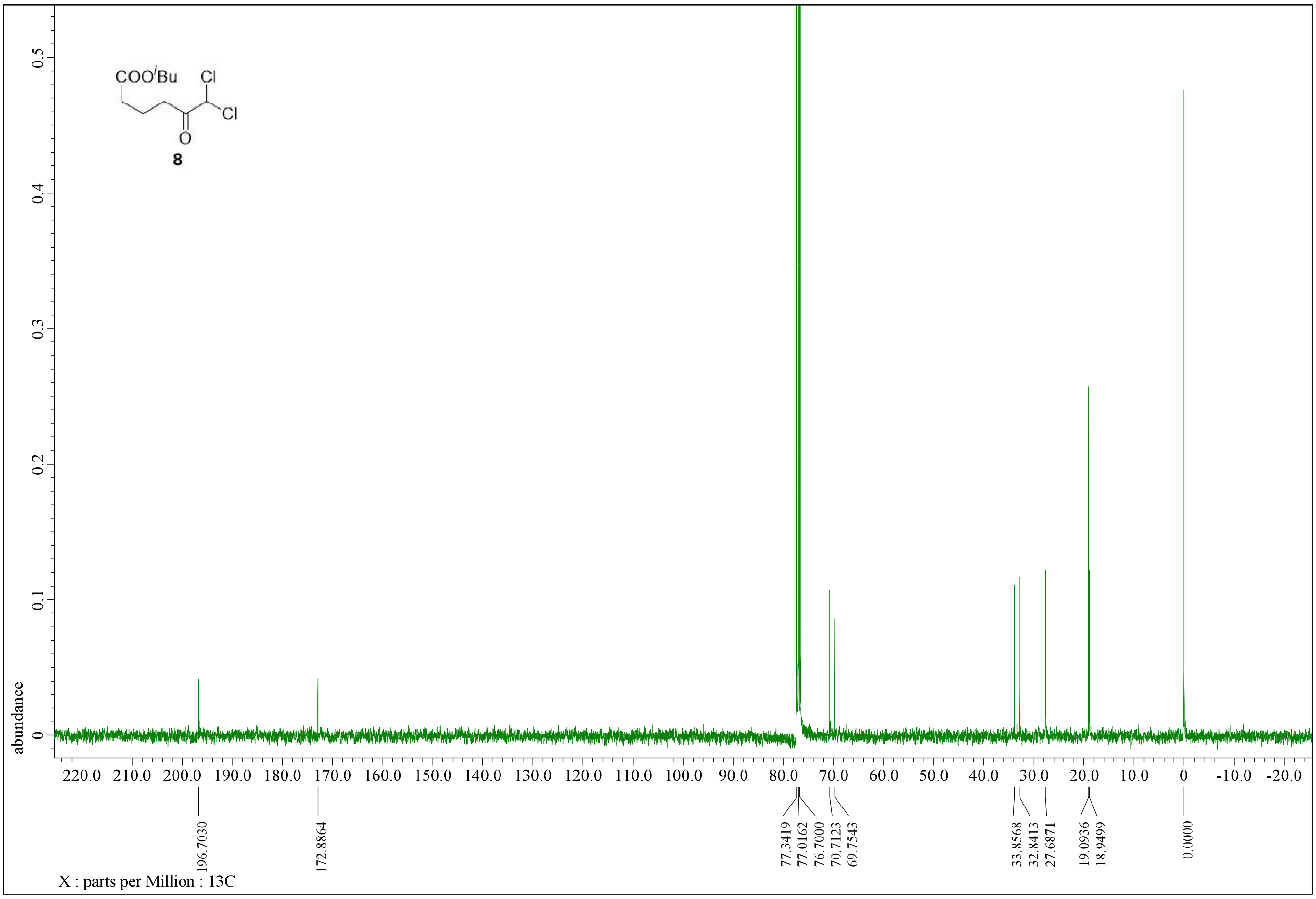
COO^iBu Cl
Cl
O
8
abundance
0.5 0.4 0.3 0.2 0.1 0
220.0 210.0 200.0 190.0 180.0 170.0 160.0 150.0 140.0 130.0 120.0 110.0 100.0 90.0 80.0 70.0 60.0 50.0 40.0 30.0 20.0 10.0 0 -10.0 -20.0
196.7030
172.8864
77.3419 77.0162 76.7000 70.7123 69.7543
33.8568 32.8413 27.6871
19.0936 18.9499
0.0000
X : parts per Million : 13C

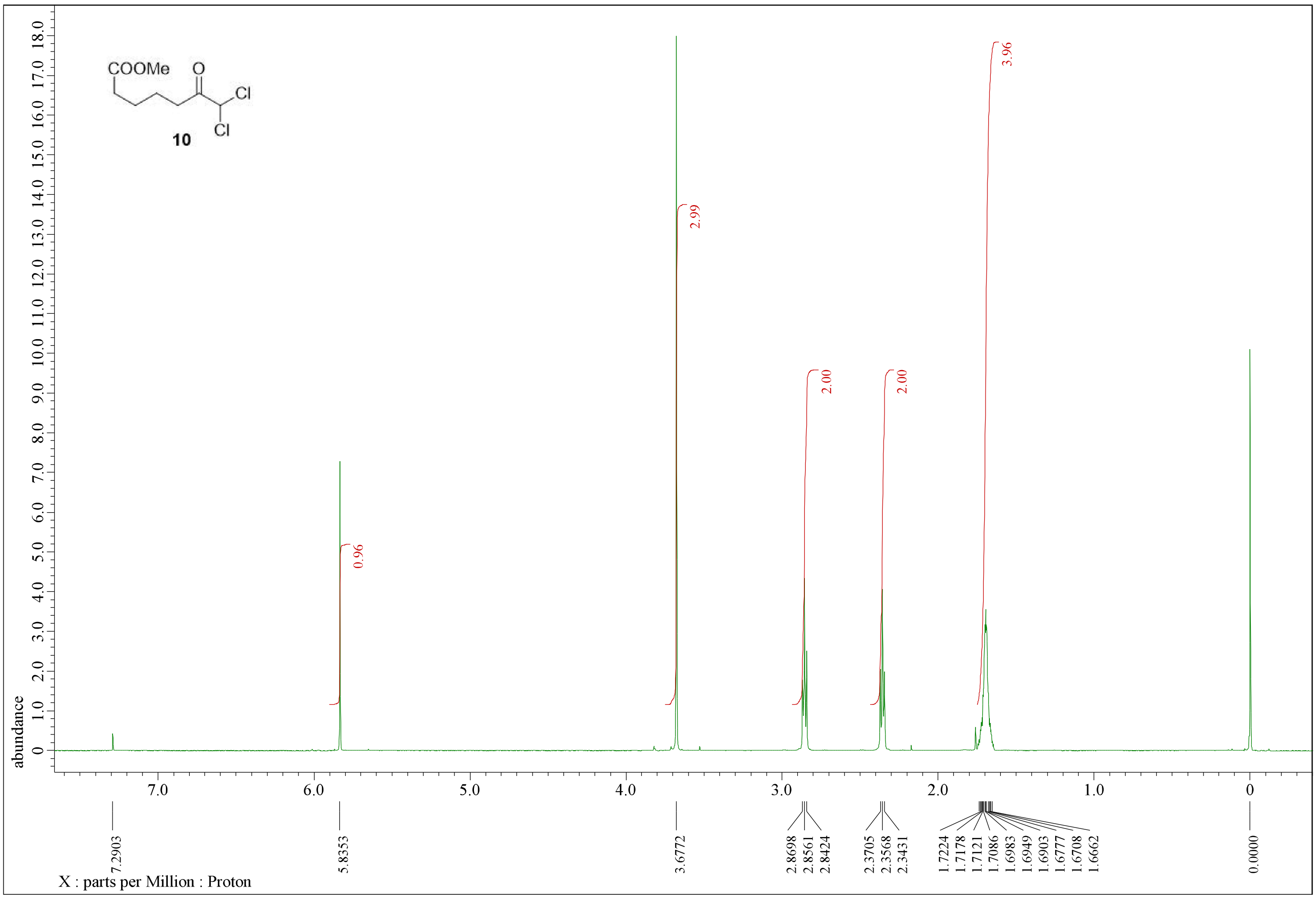
COOMe
O
Cl
Cl
10
abundance
0.96
2.99
2.00
2.00
3.96
7.2903
5.8353
3.6772
2.8698
2.8561
2.8424
2.3705
2.3568
2.3431
1.7224
1.7178
1.7121
1.7086
1.6983
1.6949
1.6903
1.6777
1.6708
1.6662
0.0000
X : parts per Million : Proton

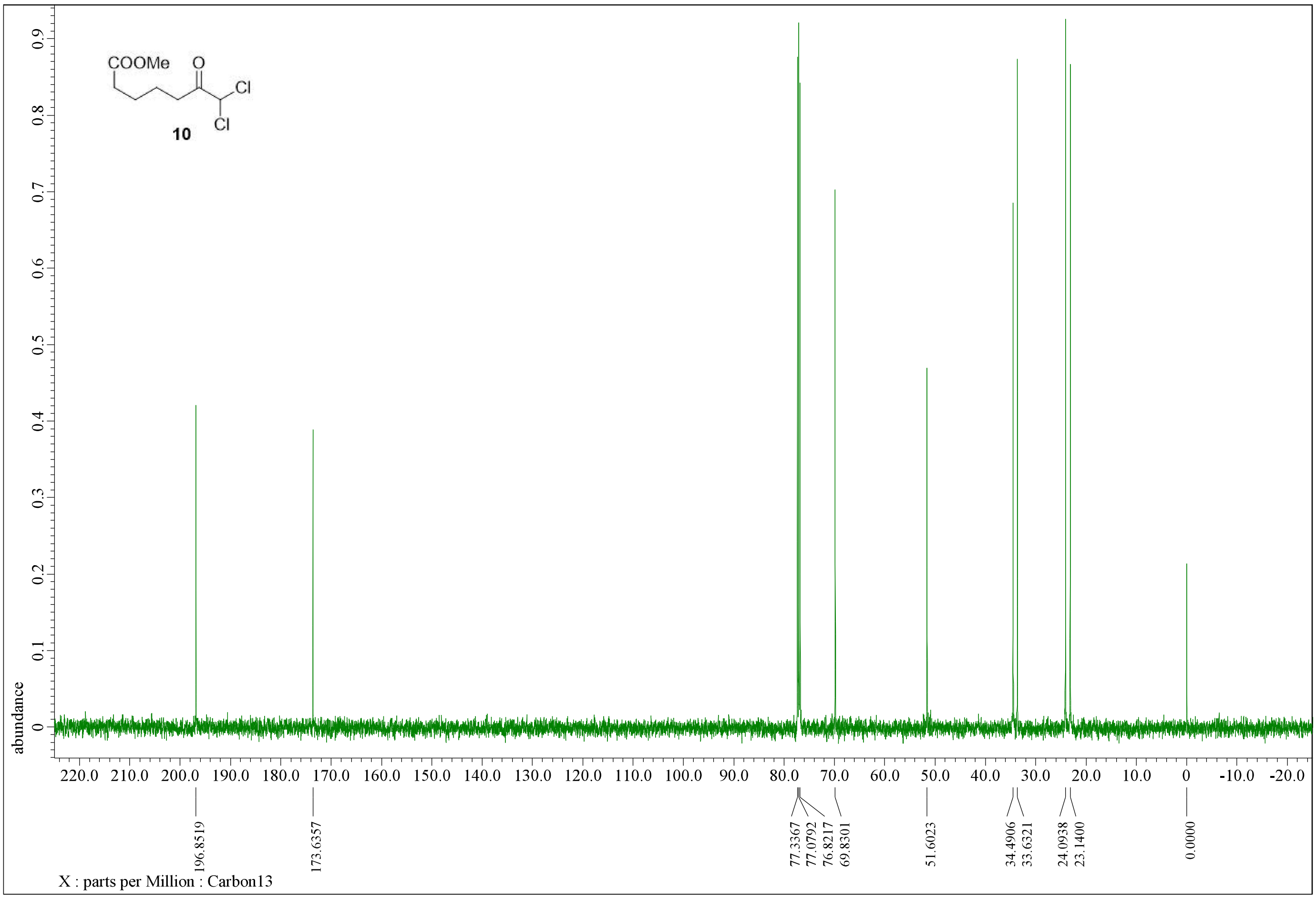
COOMe
O
Cl
Cl
10
abundance
196.8519
173.6357
77.3367
77.0792
76.8217
69.8301
51.6023
34.4906
33.6321
24.0938
23.1400
0.0000
X : parts per Million : Carbon13

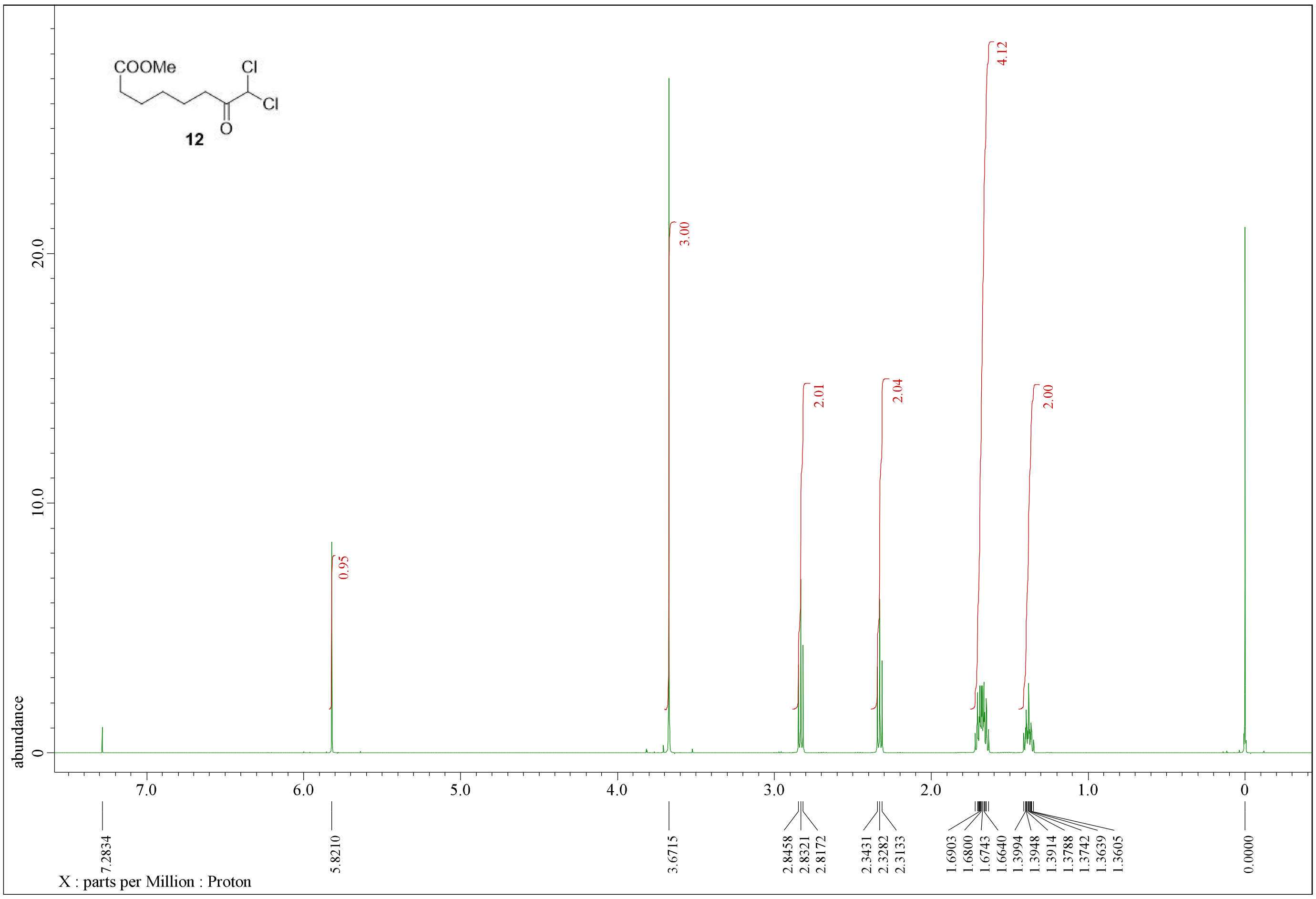
COOMe
Cl
Cl
O
12
abundance
20.0
10.0
0
0.95
3.00
2.01
2.04
4.12
2.00
7.0
6.0
5.0
4.0
3.0
2.0
1.0
0
7.2834
5.8210
3.6715
2.8458
2.8321
2.8172
2.3431
2.3282
2.3133
1.6903
1.6800
1.6743
1.6640
1.3994
1.3948
1.3914
1.3788
1.3742
1.3639
1.3605
0.0000
X : parts per Million : Proton

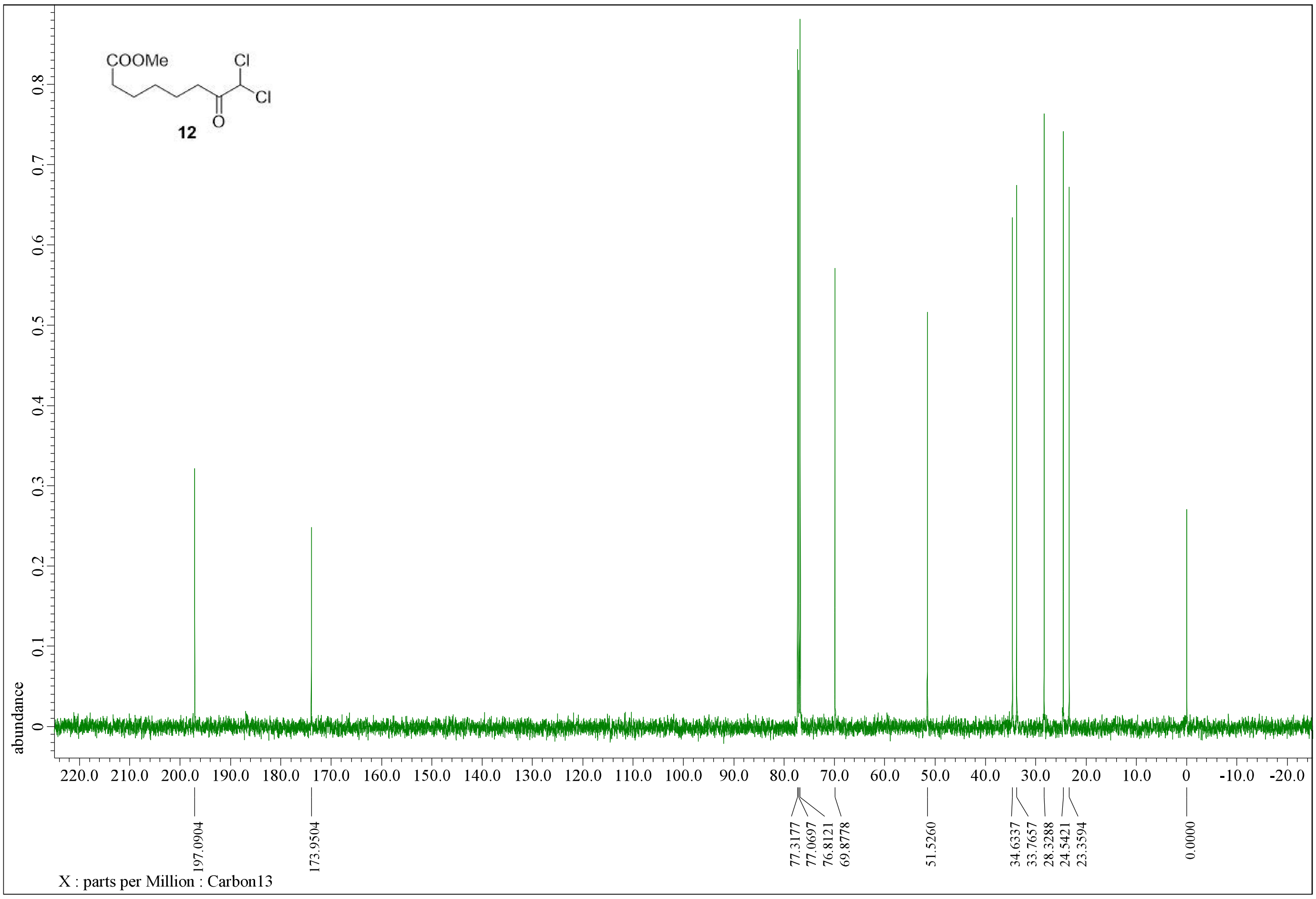
COOMe
Cl
Cl
O
12
abundance
0.8
0.7
0.6
0.5
0.4
0.3
0.2
0.1
0
220.0
210.0
200.0
190.0
180.0
170.0
160.0
150.0
140.0
130.0
120.0
110.0
100.0
90.0
80.0
70.0
60.0
50.0
40.0
30.0
20.0
10.0
0
-10.0
-20.0
197.0904
173.9504
77.3177
77.0697
76.8121
69.8778
51.5260
34.6337
33.7657
28.3288
24.5421
23.3594
0.0000
X : parts per Million : Carbon13

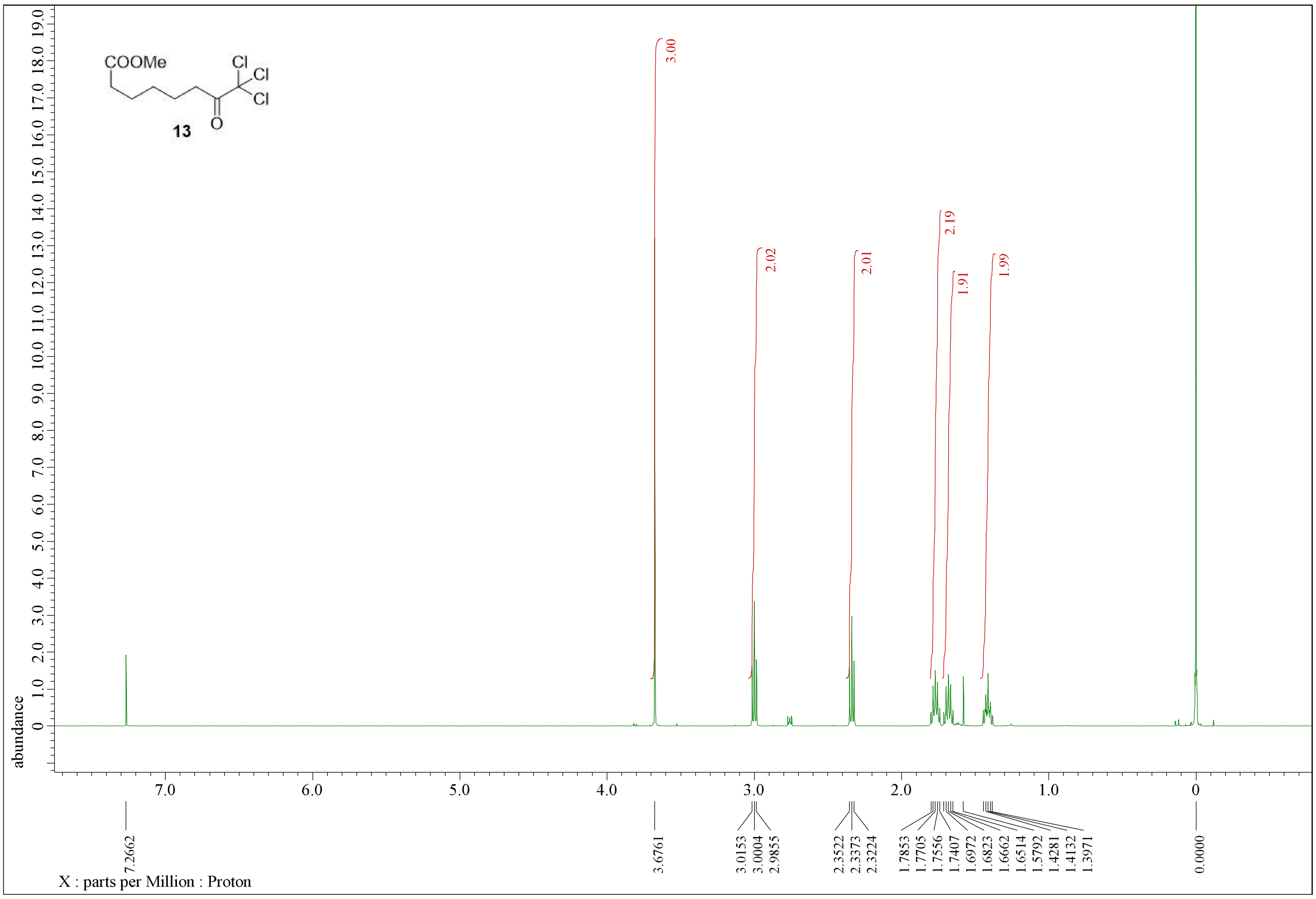
COOMe
Cl
Cl
Cl
O
13
3.00
2.02
2.01
2.19
1.91
1.99
abundance
7.2662
3.6761
3.0153
3.0004
2.9855
2.3522
2.3373
2.3224
1.7853
1.7705
1.7556
1.7407
1.6972
1.6823
1.6662
1.6514
1.5792
1.4281
1.4132
1.3971
0.0000
X : parts per Million : Proton

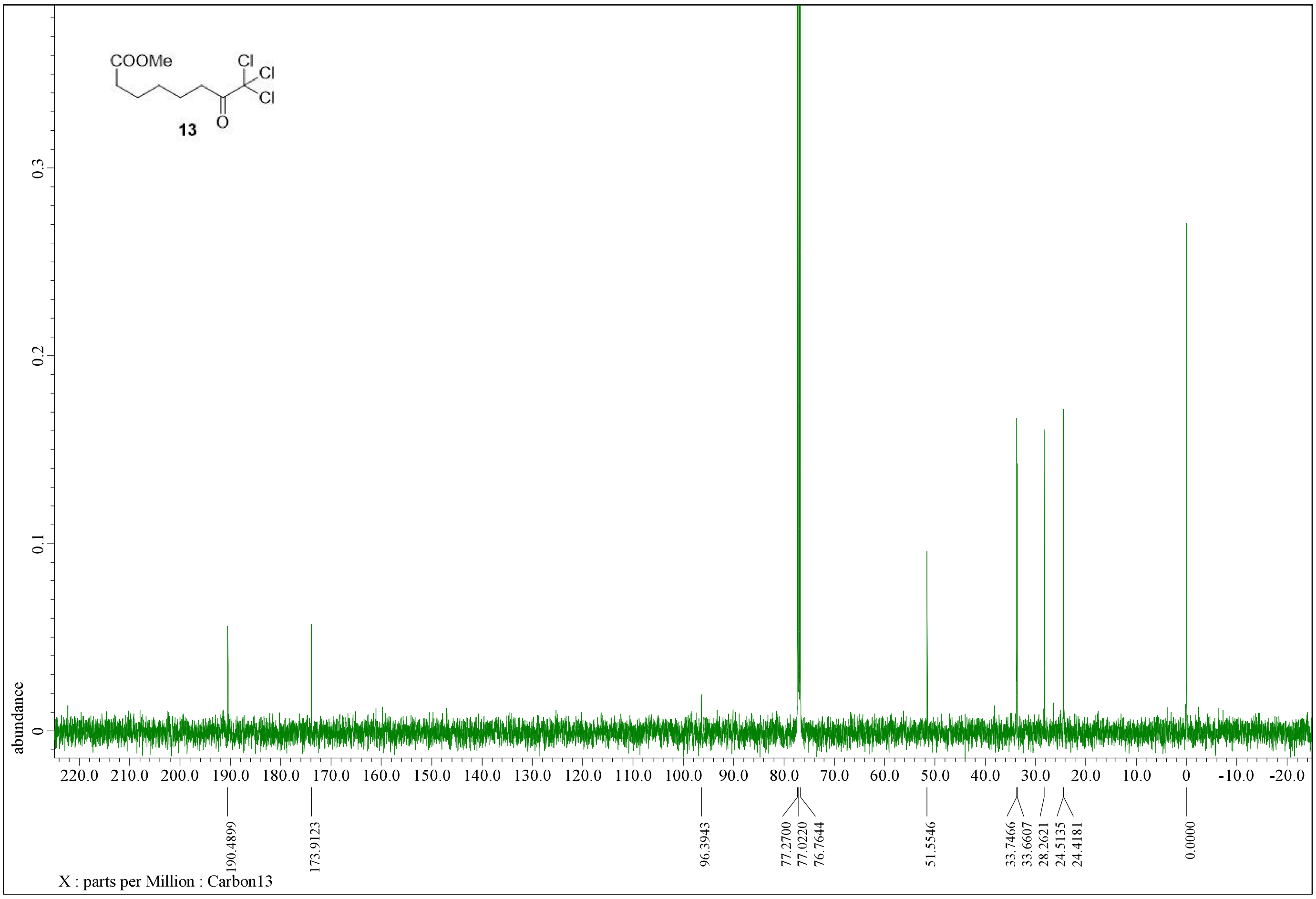
COOMe
Cl
Cl
Cl
O
13
abundance
190.4899
173.9123
96.3943
77.2700
77.0220
76.7644
51.5546
33.7466
33.6607
28.2621
24.5135
24.4181
0.0000
X : parts per Million : Carbon13

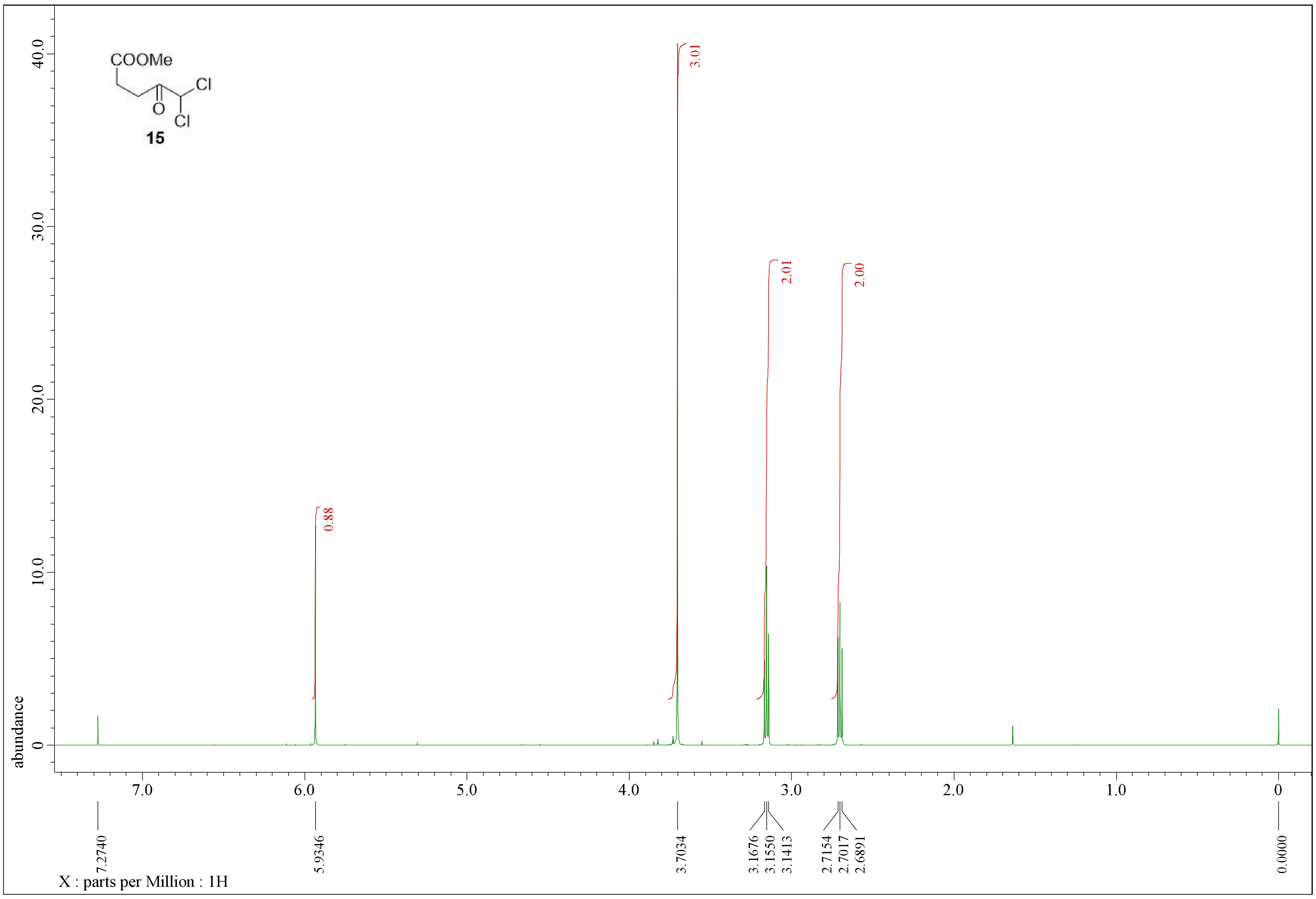
COOMe
Cl
O
Cl
15
3.01
2.01
2.00
0.88
abundance
7.0
6.0
5.0
4.0
3.0
2.0
1.0
0
7.2740
5.9346
3.7034
3.1676
3.1550
3.1413
2.7154
2.7017
2.6891
0.0000
X : parts per Million : 1H

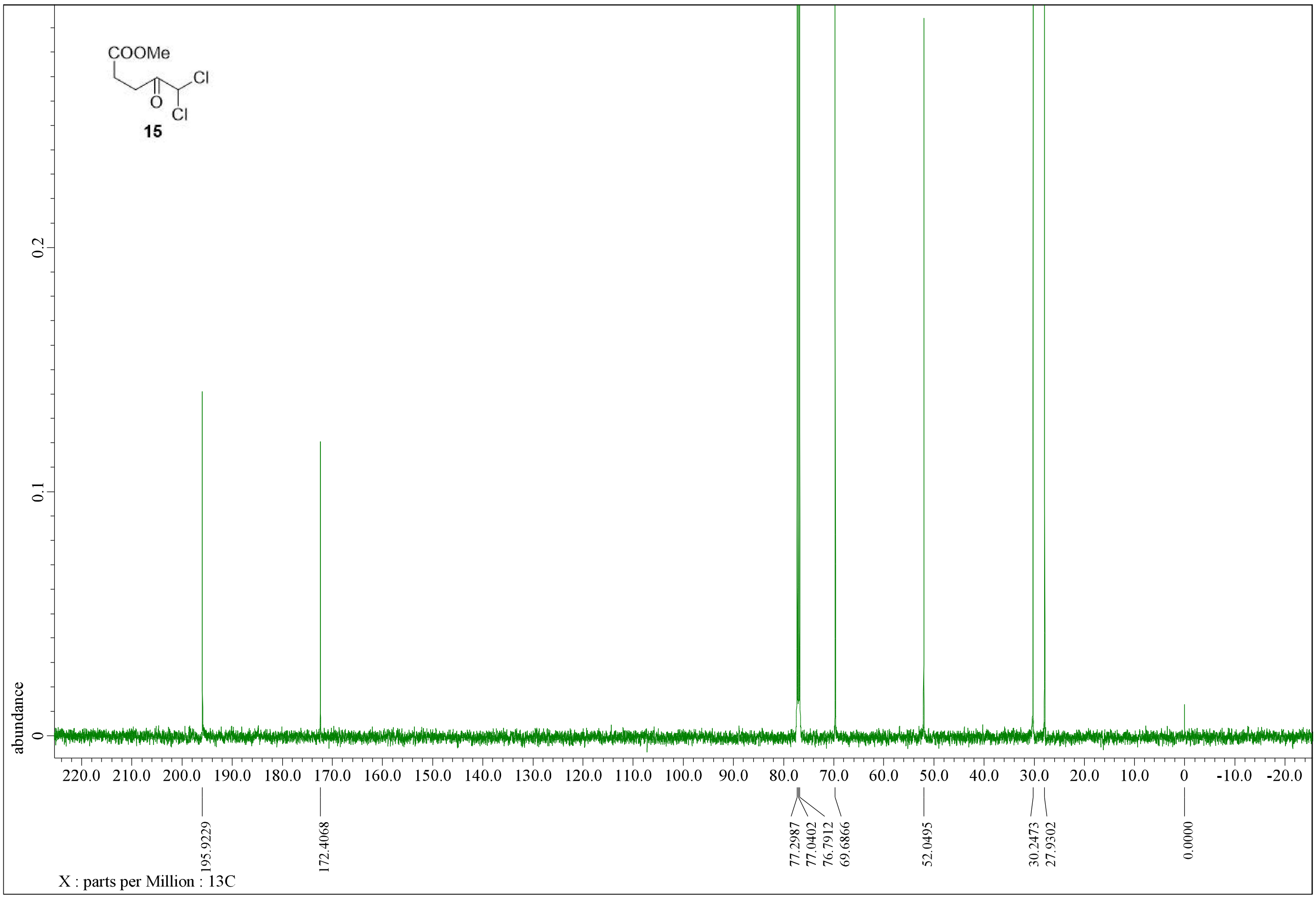
COOMe
Cl
O
Cl
15
abundance
195.9229
172.4068
77.2987
77.0402
76.7912
69.6866
52.0495
30.2473
27.9302
0.0000
X : parts per Million : 13C

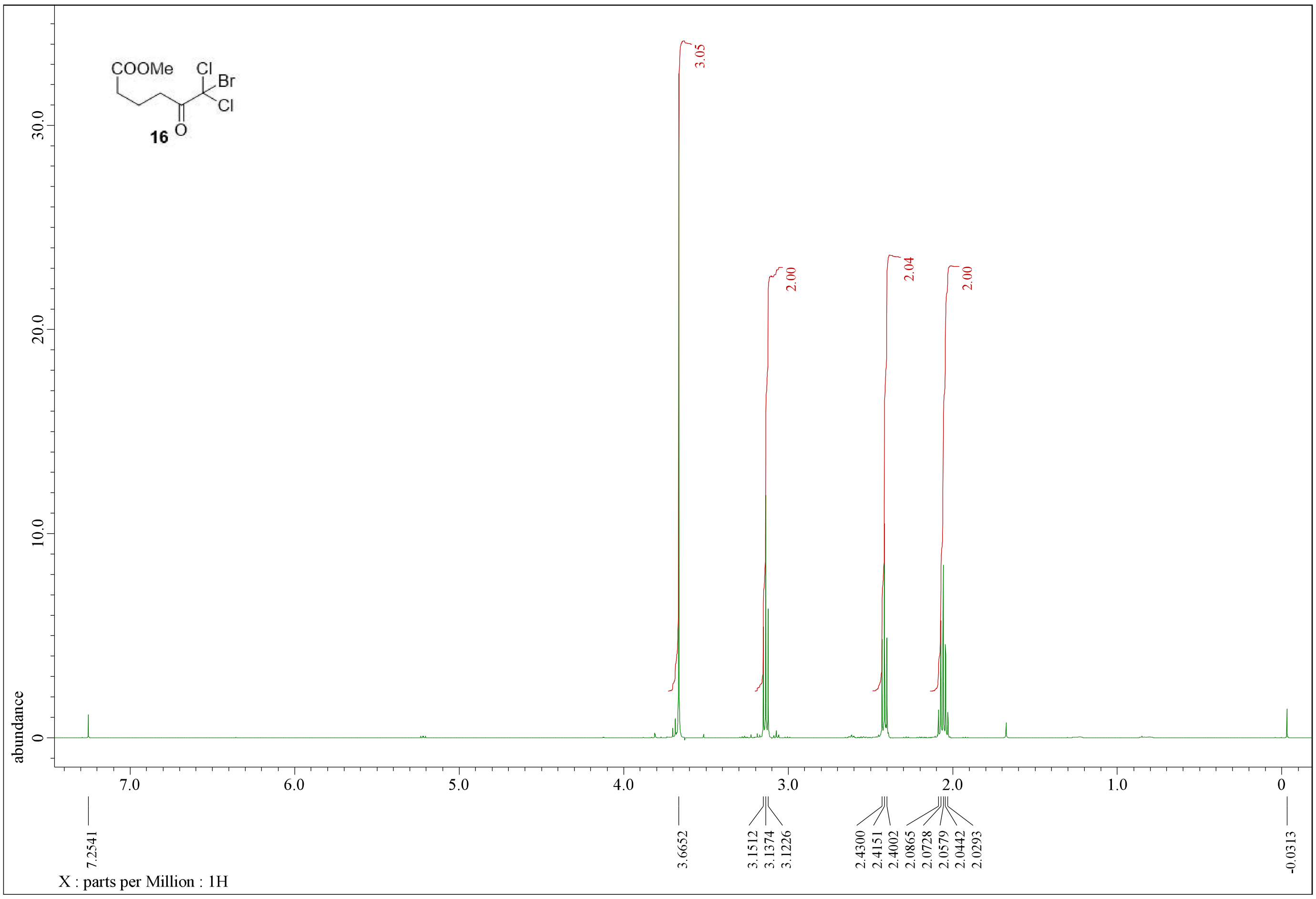
COOMe
Cl
Br
Cl
16
O
abundance
30.0
20.0
10.0
0
3.05
2.00
2.04
2.00
7.0
6.0
5.0
4.0
3.0
2.0
1.0
0
7.2541
3.6652
3.1512
3.1374
3.1226
2.4300
2.4151
2.4002
2.0865
2.0728
2.0579
2.0442
2.0293
-0.0313
X : parts per Million : 1H

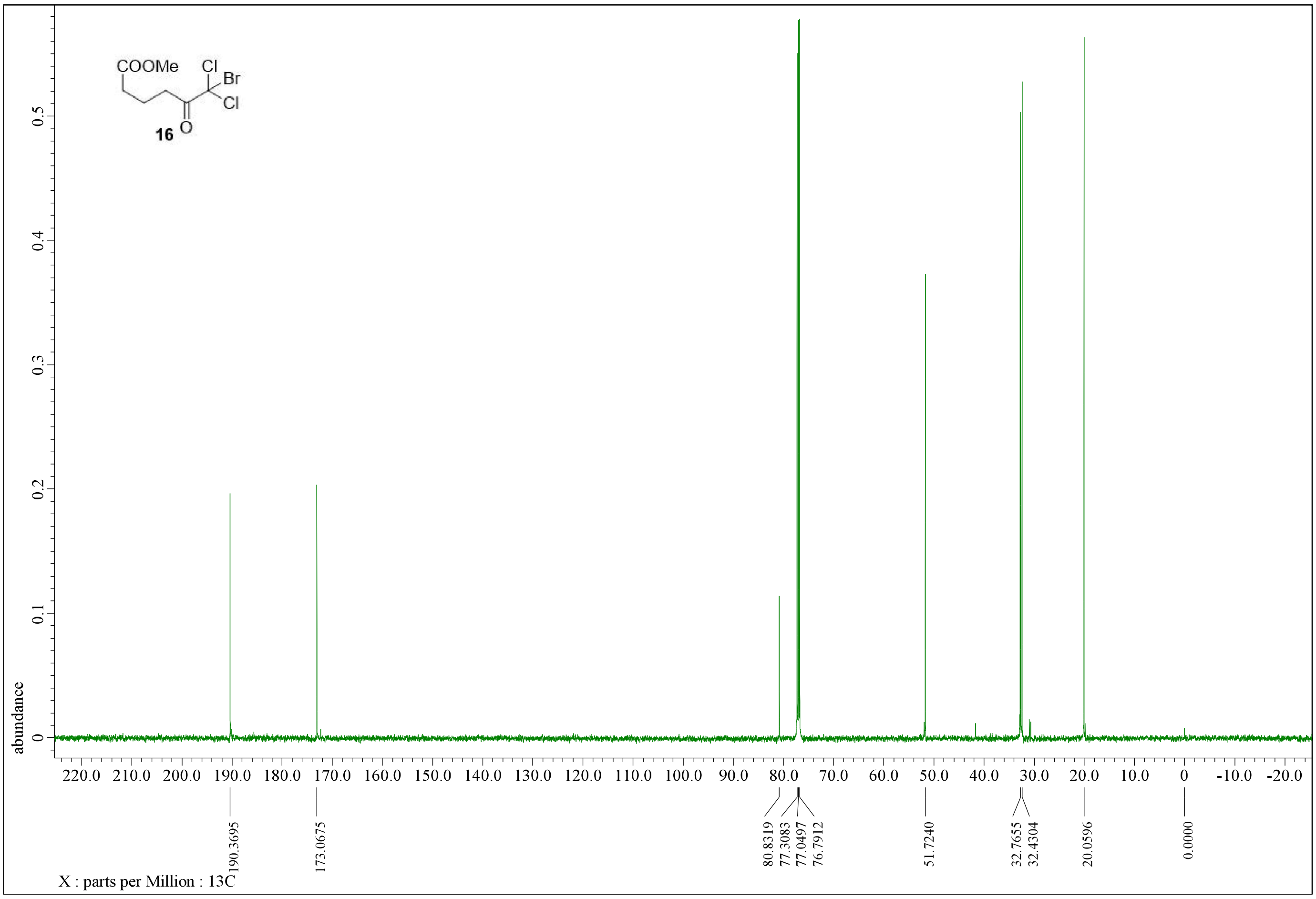
COOMe
Cl
Br
Cl
16
O
abundance
0.5
0.4
0.3
0.2
0.1
0
220.0
210.0
200.0
190.0
180.0
170.0
160.0
150.0
140.0
130.0
120.0
110.0
100.0
90.0
80.0
70.0
60.0
50.0
40.0
30.0
20.0
10.0
0
-10.0
-20.0
190.3695
173.0675
80.8319
77.3083
77.0497
76.7912
51.7240
32.7655
32.4304
20.0596
0.0000
X : parts per Million : 13C
